# A randomized multicenter trial comparing the XIENCE everolimus eluting stent with the CYPHER sirolimus eluting stent in the treatment of female patients with de novo coronary artery lesions: The SPIRIT WOMEN study

**DOI:** 10.1371/journal.pone.0182632

**Published:** 2017-08-10

**Authors:** Anna Franzone, Serge Zaugg, Raffaele Piccolo, Maria Grazia Modena, Ghada W. Mikhail, Josepa Mauri Ferré, Ruth Strasser, Liliana Grinfeld, Dik Heg, Peter Jüni, Stephan Windecker, Marie-Claude Morice

**Affiliations:** 1 Department of Cardiology, Bern University Hospital, Bern, Switzerland; 2 Clinical Trials Unit, Department of Clinical Research, University of Bern, Bern, Switzerland; 3 Institute of Cardiology, Policlinico Hospital, University of Modena and Reggio Emilia, Modena, Italy; 4 Imperial College Healthcare NHS Trust, London, United Kingdom; 5 Hospital Germans Trias I Pujol, Invasive Cardiology Unit, Badalona, Spain; 6 University of Dresden, Department of Internal Medicine and Cardiology, Heart Centre, University Hospital, Dresden, Germany; 7 Hospital Italiano, Invasive Cardiology Unit, Buenos Aires, Argentina; 8 Institute of Social and Preventive Medicine, University of Bern, Bern, Switzerland; 9 Applied Health Research Centre (AHRC), Li Ka Shing Knowledge Institute of St. Michael’s Hospital, Department of Medicine and Institute of Health Policy, Management and Evaluation, University of Toronto, Toronto, Canada; 10 Institut Cardiovasculaire Paris Sud, Paris, France; University of Messina, ITALY

## Abstract

**Background:**

The comparative performance of different drug-eluting stents (DES) among female patients has not been assessed in a randomized manner.

**Objectives:**

The SPIRIT Women Clinical Evaluation trial compared the durable polymer everolimus-eluting XIENCE stent (DP-EES) with the durable polymer sirolimus-eluting Cypher stent (DP-SES) in women undergoing percutaneous coronary intervention (PCI).

**Methods:**

A total of 455 female patients with stable CAD were randomly assigned to receive DP-EES (n = 304) or DP-SES (n = 151). The powered angiographic outcome of the trial was in-stent late lumen loss (LLL) at 9 months after the index procedure. Secondary angiographic end points included in-segment LLL, in-stent and in-segment binary restenosis and percent diameter stenosis. The primary clinical outcome was a composite of all-cause death, myocardial infarction (MI) or target vessel revascularization (TVR).

**Results:**

At 9-month follow-up, in-stent LLL was 0.19±0.38 mm and 0.11±0.37 mm in patients assigned to DP-EES and DP-SES, respectively. The one-sided upper 95% CI of the difference in in-stent LLL between the groups of 0.08 mm was 0.15 and therefore within the pre-specified non-inferiority margin of 0.17 mm (p for non-inferiority = 0.013). However, the test for superiority showed a borderline significant difference in terms of LLL between DP-EES and DP-SES (p for superiority = 0.044). There were no significant differences in binary restenosis (2.0% vs. 0.72%, p = 0.44) and percent diameter stenosis (14.97±12.17 vs. 13.36±10.82, p = 0.19). The rate of definite stent thrombosis at 12 months was lower in patients treated with DP-EES (0% vs. 2.0%, p = 0.036).

**Conclusions:**

Among women undergoing PCI, DP-EES was associated with a small but probably clinically relevant increase in in-stent LLL at 9 months as compared to DP-SES and with a lower risk of definite stent thrombosis at 12 months.

**Trial registration:**

ClinicalTrials.gov NCT01182428.

https://clinicaltrials.gov/

## Introduction

Early-generation drug-eluting stents (DES) reduced the need for repeat revascularization compared with bare-metal stents (BMS) at the expense of an increased risk of very late stent thrombosis.[1, 2] New-generation DES were conceived to improve vascular healing after stent implantation. Large scale randomized trials and meta-analyses showed better outcomes with new-generation DES compared with BMS and early-generation DES.[3–5] In these studies, however, female patients were typically underrepresented.[6, 7] Advanced age, atypical symptoms at presentation, higher burden of comorbidities and inadequate awareness of disease, indeed, are factors that usually prevent an adequate proportion of female patients with coronary artery disease (CAD) to participate into randomized trials.[8] Furthermore a tendency to use less effective treatment in female relative to male patients with CAD has been reported. [9] As consequence, treatment decisions are frequently based on overall outcomes. This is also the case for the evaluation of coronary stents. Although available evidence supports a similar performance of new-generation DES among female and male patients, [10, 11] no study has formally compared a new-generation DES with an early-generation DES in female patients.

In this context, the Clinical Evaluation of the XIENCE Everolimus Eluting Coronary Stent System in the Treatment of Women with de novo Coronary Artery Lesions (SPIRIT Women) represents a systematic attempt to evaluate the performance of the durable polymer everolimus-eluting (DP-EES) Xience stent in a population of female patients with CAD undergoing percutaneous coronary intervention (PCI).[12] The results of the principal non-randomized cohort have been previously reported.[13] Here, we reported the results of the randomized study comparing the angiographic outcomes of DP-EES with the durable polymer sirolimus eluting (DP-SES) Cypher stent.

## Material and methods

### Study population

The design of the SPIRIT Women study was reported elsewhere.[12, 13] The trial was conceived as a substudy of the main SPIRIT Women study that was registered in 2007 (NCT00496938). Subsequent changes in the original protocol were made and the randomized study was registered a separate one in 2010 (NCT01182428). Complete study protocol is available among Supporting Information files (**S1 Study Protocol**).

455 female patients admitted for PCI at 25 centers (22 in Europe and 3 in South America), between September 2008 and December 2009, were randomly assigned (2:1) to receive DP-EES (Xience V and Xience Prime, Abbott Vascular, Santa Clara, CA, USA) or DP-SES (Cypher Select, Cordis, Miami Lakes, Florida, USA). The trial was funded by Abbott Vascular. The study complied with the declaration of Helsinki and was approved by the institutional review board at each participating center (**S1 Table**). Eligible patients provided written, informed consent.

### Inclusion and exclusion criteria

Female patients, aged >18 years of age, with evidence of myocardial ischemia (e.g., stable or unstable angina, silent ischemia, positive functional study or a reversible change in the electrocardiogram consistent with ischemia) were eligible in presence of significant de novo coronary artery lesions. They had to be acceptable candidate for coronary artery bypass graft (CABG) surgery and agree to undergo all follow-up examinations. Angiographic inclusion criteria included: de novo coronary lesions (no prior stent implant, no prior brachytherapy) with reference diameter between 2.5 mm and 4.0 mm and ≤ 28 mm in length by visual estimate. There were no limitations in the number of involved vessels; however, interventions were limited to a maximum of 4 planned study stents. The most important exclusion criteria were the participation in another device or drug study or the completion of the follow-up phase of another study within the previous 30 days.

### Study devices

The XIENCE V (DP-EES) (Abbott Vascular, Santa Clara, CA, USA) is a cobalt chromium stent with durable polymeric coating consisting of acrylic and fluoropolymers eluting everolimus. At the time of this study, it was available in diameters of 2.25, 2.5, 2.75, 3.0, 3.5, 4.0 mm and lengths of 8, 12, 15, 18, 23, 28 mm. The XIENCE PRIME stent system (Abbott Vascular, Santa Clara, CA, USA) was also used where available. It featured longer cell length, taller non-linear links for improved flexibility, straighter bar arms to reduce strut interference and to better maintain coating integrity during the crimping process, and a modified proximal end ring to reduce strut lifting. The active control device was the durable polymer sirolimus-eluting Cypher stent (Cordis, Johnson & Johnson, Warren, NJ, USA)(DP-SES) consisting of sirolimus incorporated in an amalgam of two biostable polymers.

Both the DP-EES and DP-SES can have two workhorse designs, one ranging from 2.25 to 3.0mm and another covering 3.5 and 4.0 mm diameters. Furthermore, they showed comparable capacity to be overexpanded well above their labelled maximal diameter. Specifically, a maximal expansion of 4.4 mm and 4.7 mm for the Xience DP-EES and the Cypher DP-SES stents ranging from 2.25 to 3.00 mm of diameter were reported, respectively. For larger stents (3.50 to 4.0mm) the maximal capacity expansion was 5.6 mm and 5.8 mm for DP-EES and DP-SES, respectively.[14]

### Quantitative coronary angiography

Baseline and follow-up angiograms were collected in accordance with the Angiographic Core Laboratory requirements and analyzed by technicians blinded to treatment assignments and clinical outcomes.

Quantitative measurements included: reference vessel diameter, RVD, minimal luminal diameter, MLD, average of two orthogonal views of the narrowest point within the area of assessment–in lesion, in stent or in segment; percent diameter stenosis, calculated as 100 * (1—MLD/RVD) using the mean values from two orthogonal views; late lumen loss (LLL), calculated as difference between minimal lumen diameter after the procedure and minimal lumen diameter at angiographic follow-up at 9 months. Binary restenosis was defined as stenosis of ≥50% of the MLD in the target lesion at the time of angiographic follow-up. All measurements were obtained within the stented segment (in-stent) and over the entire segment comprising the stent and its 5 mm proximal and distal margins (in-segment).

### Procedures and follow-up

After confirmation of clinical and angiographic inclusion criteria and prior to PCI, patients were randomly allocated (2:1), achieving concealment of allocation by an interactive voice response system, to treatment with DP-EES or DP-SES. Randomization was stratified by diabetes mellitus, and lesion characteristics (complex vs non-complex, where complex was defined as treatment of three vessels, two lesions within one vessel, lesions involving aorto-ostial locations, bifurcations lesions with side branches ≥ 2 mm in diameter or stenosis involving the ostium of side branches). A target lesion was defined as any lesion to be treated at the time of the index procedure. The treatment strategy was determined by the investigator. If two or more target lesions had to be treated, all lesions received the device as assigned by randomization. Similarly, if any staged procedures were planned or bailout or additional stenting were needed, the same stent as allocated by randomization for the index procedure had to be used.

Peri-procedural antiplatelet therapy included a loading dose of clopidogrel (≥300 mg) and acetylsalicylic acid (≥75 mg). The choice of appropriate anticoagulants (unfractionated heparin or bivalirudin) as well as the use of glycoprotein IIb/IIIa inhibitors were left to the discretion of the investigator. Patients were maintained on dual antiplatelet therapy with clopidogrel bisulfate 75 mg daily for a minimum of six months after the procedure. Angiographic follow-up was performed at 270 days (9 months) ± 14 days. Clinical follow-up was planned at 30 days, 240 days, and 365 days after the procedure, and patients were questioned about the occurrence of angina or any adverse events. Adverse events were independently adjudicated by a clinical event committee consisting of blinded cardiologists.

### Study endpoints and definitions

The powered angiographic outcome of the trial was in-stent LLL at 9 months after the index procedure as assessed by quantitative coronary angiography. Secondary angiographic end points included in-segment LLL, in-stent and in-segment binary restenosis and percent diameter stenosis. The primary clinical outcome, which was descriptive in nature, was a composite of all-cause death, myocardial infarction (MI) or target vessel revascularization (TVR). Secondary clinical outcomes were the components of the primary outcome, cardiac death, target lesion revascularization (TLR), target lesion failure (TLF) defined as the composite of cardiac death, target-vessel MI, and ischemia-driven TLR and definite and probable stent thrombosis. According to Academic Research Consortium criteria, (1) all deaths were considered cardiac unless an unequivocal non-cardiac cause was established. Any death due to proximate cardiac cause, unwitnessed death and death of unknown cause, and all procedure-related deaths, including those related to concomitant treatment, were classified as cardiac death. Myocardial infarction (MI) was defined as follows: for non-procedural/spontaneous MI, troponin or creatine kinase muscle and brain (CK-MB) levels had to be >2 times the upper limit of normal; for peri-procedural MI, troponin or CK-MB levels had to be ≥3 times the upper limit of normal. The peri-procedural period included the first 48 hours and 72 hours after PCI and CABG, respectively. All late events that were not associated with revascularization procedures were considered spontaneous. TLR was defined as any repeat percutaneous intervention of the target lesion or bypass surgery of the target vessel performed for restenosis or other complications of the target lesion. The target lesion was defined as the treated segment from 5 mm proximal and 5 mm distal to the stent. TVR was defined as any repeat percutaneous intervention or surgical bypass of any segment of the target vessel. Stent thrombosis was defined on the basis of the Academic Research Consortium criteria. Clinical device success was defined as successful delivery and deployment of the study stent at the intended target lesion and successful withdrawal of the stent delivery system with attainment of final residual stenosis of less than 50% of the target lesion by visual estimation, without use of a device outside the assigned treatment strategy. Clinical procedure success was defined as successful delivery and deployment of the study stent or stents at the intended target lesion and successful withdrawal of the stent delivery system with attainment of final residual stenosis of less than 50% by visual estimation, using any adjunctive device without the occurrence of cardiac death, MI attributed to the target vessel or TLR during the hospital stay with a maximum of first seven days post index procedure.

### Statistical analysis

The study was designed as a non-inferiority trial powered for the pre-specified primary angiographic endpoint in-stent LLL at 9 months. Sample size calculation for the primary endpoint was based on the following assumptions: One-sided non-inferiority test with α = 5% and power = 90%, 2:1 randomization ratio (EES:SES), in-stent LLL equal in both arms with a common standard deviation of 0.53 mm, and a non-inferiority margin δ = +0.17 mm (difference defined as LLL_EES_ minus LLL_SES_). Given the above assumptions, a total of 378 subjects were required. To account for dropouts at angiographic follow-up, 450 subjects were enrolled.

The non-inferiority p-value for the angiographic primary outcome was one-sided and derived from a Z-test that compared the difference between groups against the non-inferiority margin. For ease of interpretation, all other p-values and 95% confidence intervals are two-sided, for superiority. Since the two-sided 95% confidence interval of the primary endpoint excluded the line of no difference, we also calculated two-sided p-values for superiority for the primary endpoint.[15]

Continuous variables were expressed as mean ± standard deviations (SD) and compared with t-test; categorical variables were summarized as frequencies (%) and compared with Chi-square test or Fisher’s exact test if the expected number of events was below 5 per cell. p-values were derived from general or generalized linear mixed models that account for the correlation of multiple lesions within patients. The Mantel–Cox method was used to compare outcomes between patients randomized to DP-SES or DP-EES, reporting rate ratios (RRs) with 95% confidence intervals (CI). In case of zero events in any comparator group, we reported continuity corrected risk ratios with p-values from Fisher’s exact tests. All patients who underwent randomization were included in the analysis of primary and secondary outcomes in the groups to which they were originally allocated to, regardless of the treatment actually received (intention-to-treat principle). No adjustments were made for multiple comparisons. Analyses were performed by a statistician of an academic clinical trials unit (CTU Bern, Bern University Hospital, Switzerland) with Stata 13 (Stata, Inc., College Station, TX, USA).

## Results

**Fig 1:**
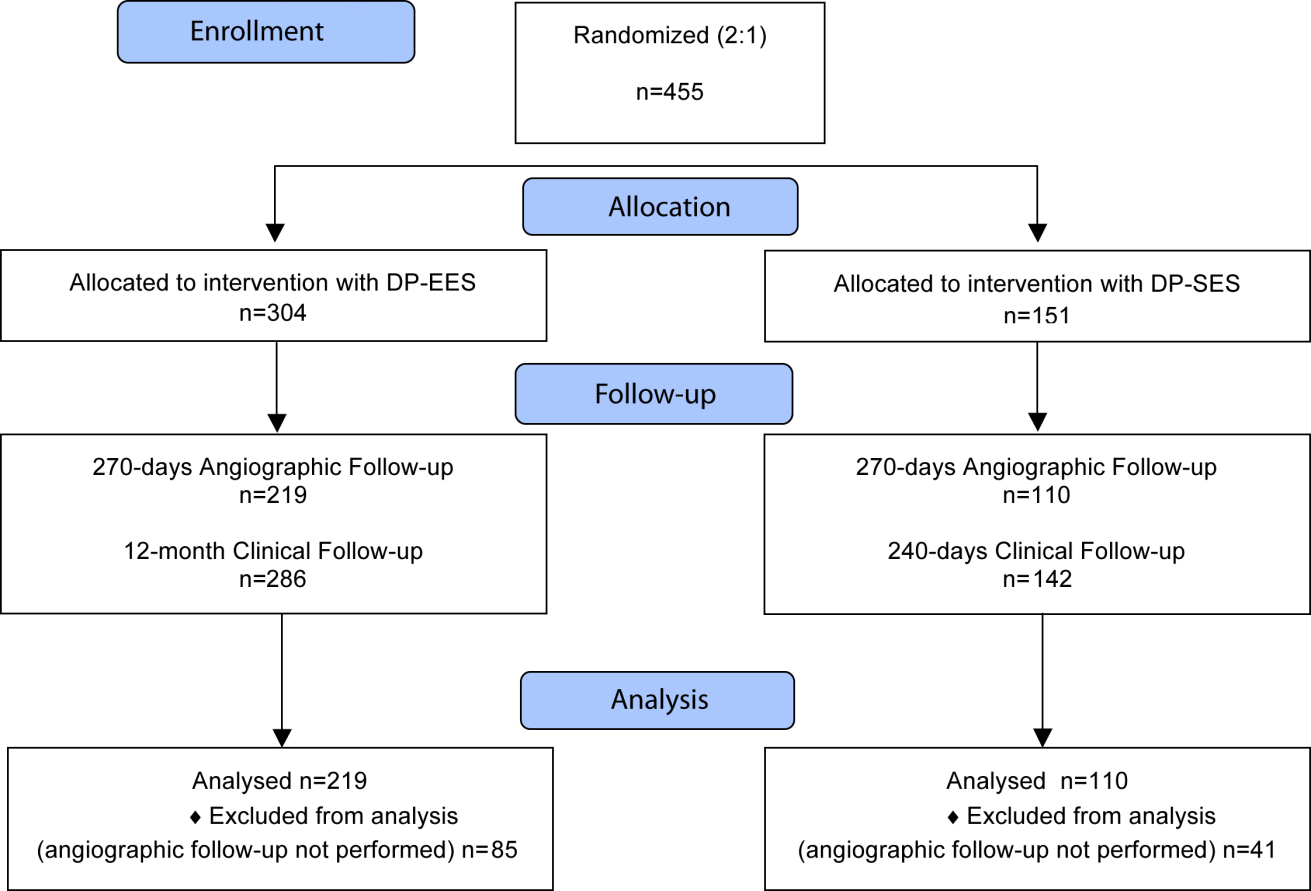
Flow diagram of patients included in the trial (according to CONSORT 2010). A total of 455 patients were randomly assigned to treatment with DP-EES (304 patients) or DP-SES (151 patients). Number of patients assessed for elegibility was not available. DP-EES, Durable polymer-everolimus eluting stent; DP-SES, Durable polymer-sirolimus eluting stent.

A total of 455 patients were randomly assigned to undergo treatment with DP-EES (304 patients, 423 lesions) or DP-SES (151 patients, 200 lesions). The flow diagram of patients included in the trial is shown in **Fig 1.** Baseline clinical characteristics are reported in **Table 1**. The majority of patients in both arms had stable CAD with a high prevalence of cardiovascular risk factors. Angiographic and procedural features are summarized in **S2 Table** and were similar for both groups with the exception of three-vessel treatment that was more common in patients assigned to DP-EES. Clinical procedure success (88.2% vs. 87.4%, p = 0.88) and device success (99.3% vs. 99.5%, p = 1.00) were not different between groups.

**Table 1:** Baseline clinical characteristics.

	DP-EES	DP-SES
	n = 304	n = 151
**Age**	68.3 ± 10.1	69.8 ± 10.6
**Waist circumference (cm)**	93.8 ± 15.2	93.8 ± 15.2
**BMI (kg/m**^**2**^**)**	27.5 ± 5.1	27.4 ± 5.5
**Current tobacco user**	52 (17.4%)	24 (16.3%)
**Postmenopausal status**	284 (94.4%)	144 (95.4%)
**Hypertension**	253 (83.5%)	130 (86.1%)
**Lipid disorder**	224 (73.7%)	111 (74.0%)
**Diabetes Type II**	74 (24.3%)	41 (27.2%)
**Oral hypoglycemic**	46 (62.2%)	21 (51.2%)
**Insulin**	19 (25.7%)	12 (29.3%)
**Previous PCI**	60 (19.8%)	31 (20.7%)
**Previous MI**	79 (26.1%)	31 (20.7%)
**MI within 2 months of screening date**	27 (34.2%)	10 (32.3%)
**Family history of premature CAD**	109 (35.9%)	55 (36.4%)
***Clinical presentation***		
**Stable angina**	152 (50.2%)	81 (54.0%)
**Unstable angina**	116 (38.3%)	48 (32.0%)
**Silent ischemia**	35 (11.6%)	21 (14.0%)
**Left ventricular dysfunction**	43 (15.6%)	19 (14.6%)
***Anginal symptoms***		
**Typical angina**	234 (77.5%)	119 (78.8%)
**Atypical chest pain**	31 (10.3%)	14 (9.3%)
**No chest pain**	37 (12.3%)	18 (11.9%)

Data expressed as n (%) or means ± standard deviations. BMI, Body mass index; CAD, Coronary artery disease; DP-EES, Durable polymer- everolimus eluting stents; DP-SES, Durable polymer- sirolimus eluting stents; MI, Myocardial infarction; PCI, Percutaneous coronary intervention

### Angiographic outcomes

**Fig 2:**
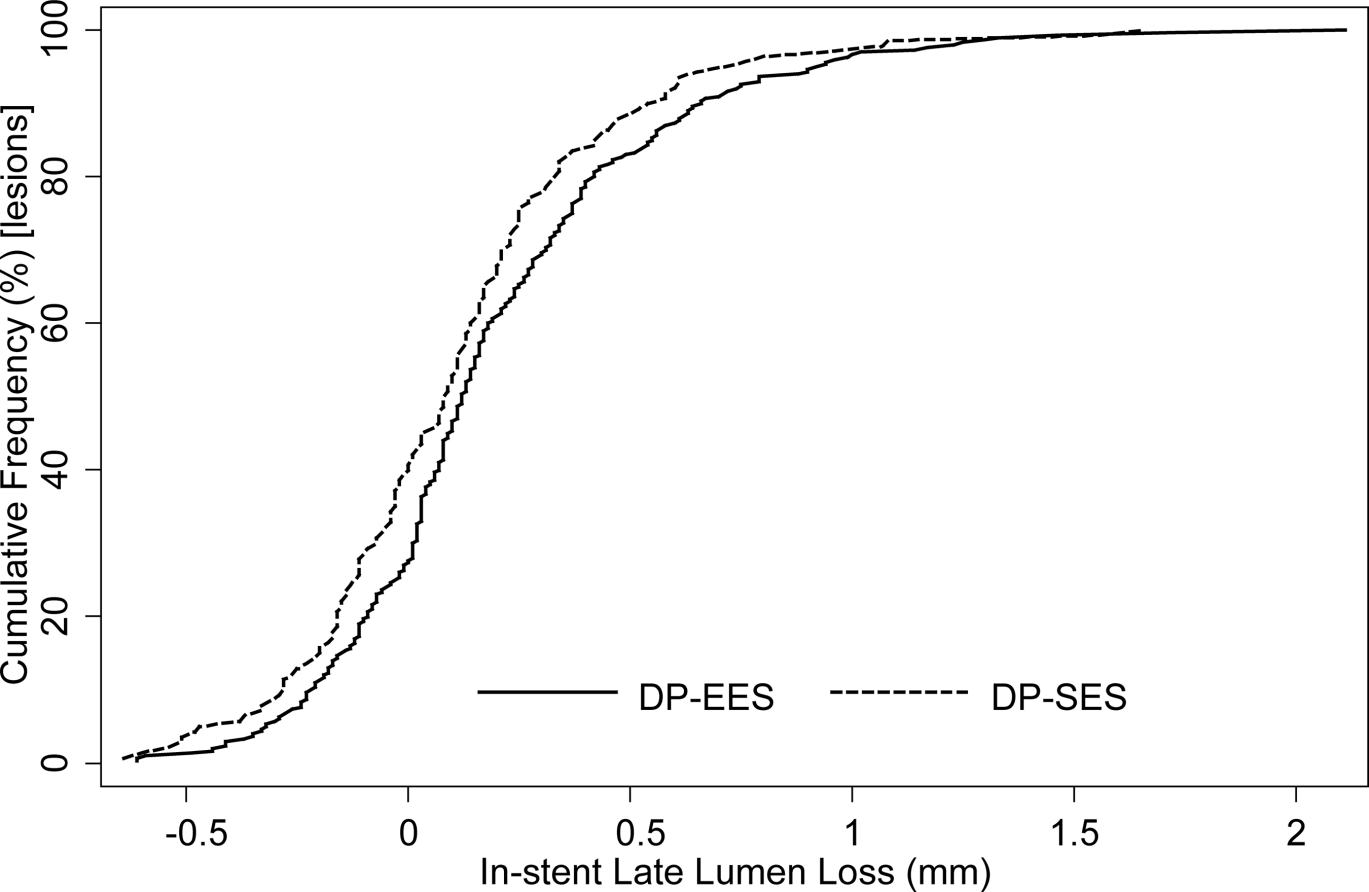
Cumulative distribution of the primary end point in-stent late lumen loss shown separately for the two stent types. DP-EES, Durable polymer-everolimus eluting stent = solid line; DP-SES, Durable polymer-sirolimus eluting stent = dashed line.

**Fig 3:**
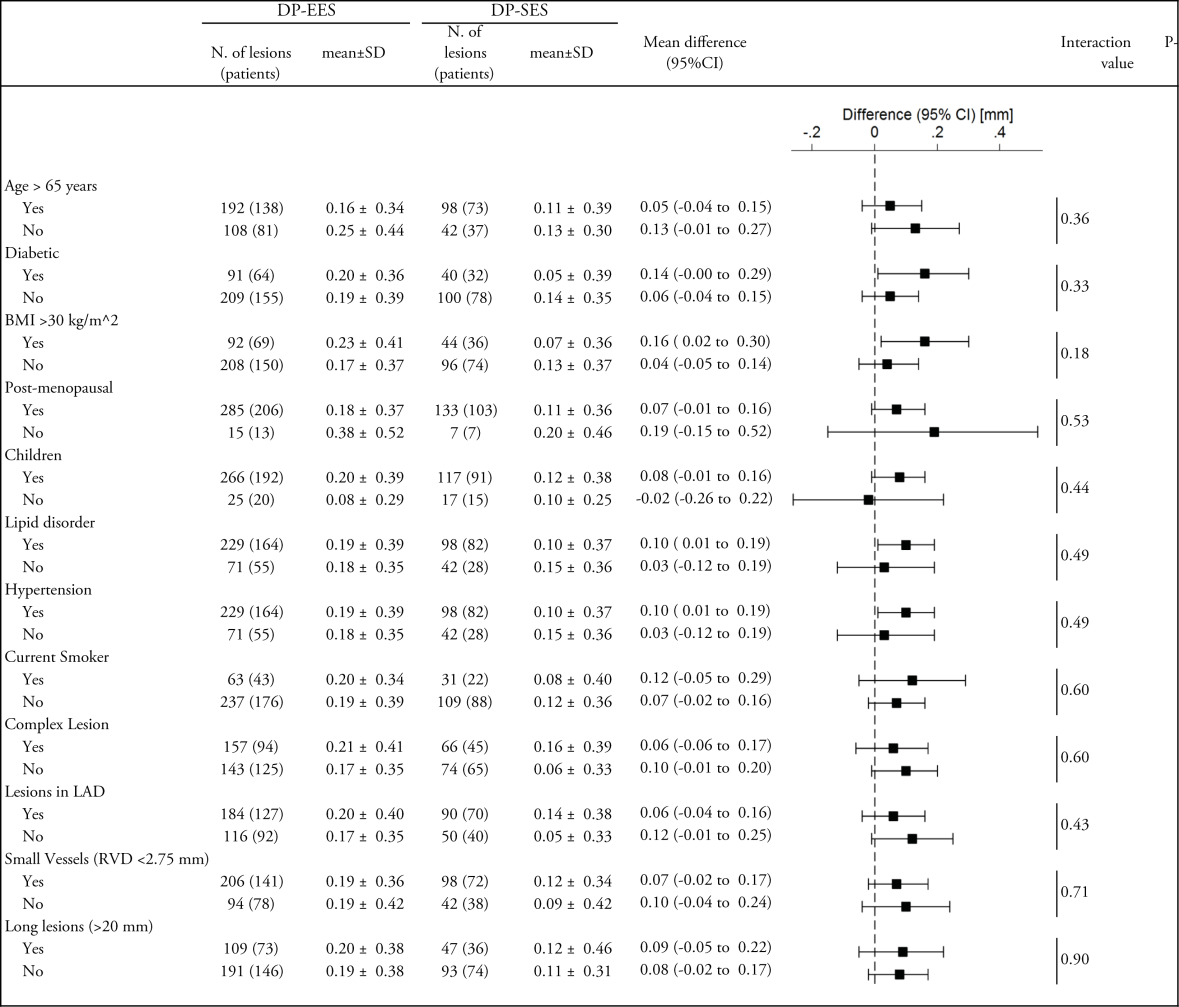
Stratified analysis of in-stent late lumen loss (LLL). Two-sided p-values and CIs from linear mixed models with random intercepts at level of patients. BMI, Body mass index; DP-EES, Durable polymer- everolimus eluting stents; DP-SES. Durable polymer-sirolimus eluting stent; LAD, Left anterior descending artery; RVD, Reference vessel diameter.

At 9 months, 219 patients in the DP-EES group (72%) and 110 patients in the DP-SES group (73%) underwent angiographic follow-up. As shown in **S3 Table**, baseline patient characteristics were comparable between arms for this subset of patients. There were not significant differences between both groups for angiographic measurements immediately after the index procedure (**Table 2**). At 9-month follow-up, in-stent LLL was 0.19±0.38 mm and 0.11±0.37 mm in patients assigned to DP-EES and DP-SES, respectively. The one-sided upper 95% CI of the difference in in-stent LLL between the groups of 0.08 mm was 0.15 and therefore within the pre-specified non-inferiority margin of 0.17 mm (p for non-inferiority = 0.013). However, the test for superiority showed a borderline significant difference in terms of LLL between DP-EES and DP-SES (p for superiority = 0.044). **Fig 2**reports the cumulative distribution of the primary endpoint separately for the study devices. In-stent binary restenosis (2.0% vs. 0.72%, p = 0.44) and percent diameter stenosis (14.97%±12.17 vs. 13.36%±10.82, p = 0.19) were similar between groups. In-segment LLL was 0.09±0.40 mm in the DP-EES group and 0.03±0.38 in the DP-SES group (p = 0.166). In-segment binary restenosis was 3.3% and 2.2%, respectively (p = 0.76). **Fig 3**shows the stratified analysis for the primary endpoint in several subgroups without significant differences in the pre-specified subsets of patients with diabetes and patients with complex lesions.

**Table 2:** Quantitative coronary angiography analysis after index procedure and at 9-month follow-up.

	DP-EES	DP-SES	Difference (95%-CI) [EES-SES]	p value
**N. Patients**	219	110		
**N. Lesions**	300	140		
After index procedure*				
**Stent length (mm)**	19.0 ± 9.29	18.3 ± 7.10	0.71 (-1.11 to 2.52)	0.44
**Segment length (mm)**	26.92 ± 9.50	26.65 ± 7.71	0.31 (-1.55 to 2.17)	0.74
**Diameter Stenosis (%)**				
**In-stent**	9.11 ± 6.57	10.12 ± 8.80	-1.02 (-2.54 to 0.49)	0.18
**In-segment**	17.99 ± 8.21	17.94 ± 9.50	-0.08 (-1.86 to 1.70)	0.92
**RVD (mm)**				
**In-stent**	2.76 ± 0.45	2.77 ± 0.45	-0.02 (-0.11 to 0.08)	0.72
**In-segment**	2.64 ± 0.49	2.67 ± 0.50	-0.02 (-0.13 to 0.08)	0.65
**MLD (mm)**				
**In-stent**	2.50 ± 0.41	2.50 ± 0.47	0.00 (-0.09 to 0.09)	0.96
**In-segment**	2.17 ± 0.48	2.19 ± 0.51	-0.02 (-0.12 to 0.08)	0.72
At 9-month follow-up*				
**Stent Length (mm)**	19.27 ± 9.56	18.92 ± 8.53	0.49 (-1.48 to 2.45)	0.62
**Segment Length (mm)**	27.24 ± 9.66	26.98 ± 8.43	0.36 (-1.60 to 2.31)	0.72
**Diameter Stenosis (%)**				
**In-stent**	14.97 ± 12.17	13.36 ± 10.82	1.63 (-0.82 to 4.08)	0.19
**In-segment**	21.73 ± 12.43	20.09 ± 12.60	1.65 (-0.89 to 4.19)	0.20
**RVD (mm)**				
**In-stent**	2.72 ± 0.44	2.75 ± 0.47	-0.03 (-0.13 to 0.06)	0.52
**In-segment**	2.66 ± 0.46	2.69 ± 0.50	-0.03 (-0.13 to 0.07	0.59
Binary Restenosis (%)**				
**In-stent**	6 (2.0%)	1 (0.72%)		0.44
**In-segment**	10 (3.3%)	3 (2.2%)		0.76
**MLD (mm)**				
**In-stent**	2.31 ± 0.53	2.39 ± 0.51	-0.08 (-0.19 to 0.03)	0.15
**In-segment**	2.08 ± 0.49	2.16 ± 0.56	-0.08 (-0.18 to 0.03)	0.16
**LLL (mm)**				
**In-stent**	0.19 ± 0.38	0.11 ± 0.37	0.08 (0.00 to 0.16)	0.044
**In-segment**	0.09 ± 0.40	0.03 ± 0.38	0.06 (-0.02 to 0.14)	0.16

* Analysis based on subset of serial lesions, i.e. with post-procedural and follow-up angiography available. Two-sided p-values and CIs from linear mixed models (LMM) with random intercepts at level of patients. Non-inferiority p-value is one-sided from a Z-test constructed from the LMM-based standard error of the difference.

**p-value from Fisher's exact test. LLL, Late lumen loss; MLD, Minimal luminal diameter; RVD, Reference vessel diameter.

### Clinical outcomes

At one year, clinical follow-up was available in 286 DP-EES (94%) and 142 DP-SES (94%) patients. As shown in **S1 Fig**, DAPT use was similar in both groups throughout the 1-year follow-up (p = 0.67). Clinical outcomes are reported in **Table 3**. The composite endpoint of death, MI or TVR occurred in 20.7% and 21.9% of patients in the DP-EES and DP-SES group, respectively (risk ratio 0.93; 95% CI 0.61–1.43, p = 0.75). Rates of TLF (15.3% vs. 17.3%, risk ratio 0.87, 95% CI 0.54–1.41, p = 0.57), MI (11.2% vs. 13.3%, risk ratio 0.83; 95% CI 0.48–1.45, p = 0.50), and clinically-indicated TLR (5.1% vs. 5.5%, risk ratio 0.93, 95% CI 0.40–2.20, p = 0.88) were similar in both groups (**S2 Fig**). Overall, three cases of definite ST were reported among patients allocated to DP-SES. One patient died for an acute ST; a second one died as consequence of subacute ST (6 days after the procedure) and the third one experienced a late, non-fatal ST after premature DAPT discontinuation. No definite ST was documented in patients allocated to DP-EES. Thus, the cumulative incidence of definite ST at 1-year was 2.0% and 0% in patients assigned to DP-SES and DP-EES, respectively (p = 0.036) (**S4 Table**).

**Table 3:** Clinical outcomes at 1 year follow-up.

	DP-EES	DP-SES	Rate Ratio	p value
	n = 304	n = 151	(95% CI)	
**Death**	3 (1.0%)	6 (4.0%)	0.25 (0.06 to 0.98)	0.06^§^
**Cardiac death**	2 (0.7%)	4 (2.7%)	0.25 (0.04 to 1.34)	0.09^§^
**MI**	39 (12.9%)	21 (13.9%)	0.91 (0.54 to 1.58)	0.73
**Target vessel-MI**	34 (11.2%)	20 (13.3%)	0.82 (0.46 to 1.48)	0.52
**Any revascularization**	51 (17.4%)	29 (19.9%)	0.87 (0.55 to 1.38)	0.56
**Clinically-indicated revascularization**	45 (15.3%)	23 (15.8%)	0.97 (0.59 to 1.61)	0.91
**Any TVR**	28 (9.6%)	17 (11.7%)	0.82 (0.45 to 1.49)	0.51
**Clinically-indicated TVR**	19 (6.5%)	14 (9.6%)	0.66 (0.33 to 1.33)	0.24
**Any TLR**	23 (7.8%)	12 (8.3%)	0.96 (0.48 to 1.93)	0.91
**Clinically-indicated TLR**	15 (5.1%)	8 (5.5%)	0.93 (0.40 to 2.21)	0.88
**Target lesion failure** *	46 (15.3%)	26 (17.3%)	0.87 (0.52 to 1.43)	0.57
Death, MI, TVR**	62 (20.7%)	33 (21.9%)	0.93 (0.60 to 1.44)	0.75
**Stroke or TIA**	3 (1.0%)	2 (1.4%)	0.74 (0.12 to 4.43)	0.66^§^
**Definite ST**	0 (0.0%)	3 (2.0%)	0.07 (0.00 to 1.35)	0.036^§^
**Probable ST**	0 (0.0%)	0 (0.0%)	-	-

Number of first events and percentages are reported. Rate ratios RR (95% CI) are estimated using the Mantel-Cox method with two-sided p-values from log-rank test. Clinical outcomes analyzed on patient level. All events were censored beyond 365 days.

^§^p-value from 2 sided Fisher's exact test.

*Cardiac death, TV-MI, clinically indicated TLR

**Primary endpoint.

MI, Myocardial infarction; ST, Stent thrombosis; TIA, Transient ischemic attack; TLR, Target lesion revascularization; TVR, Target vessel revascularization.

## Discussion

The main findings of this randomized trial comparing DP-EES with DP-SES in a female population undergoing PCI were as follows:

- Even though DP-EES was formally non-inferior to DP-SES with respect to the primary endpoint of LLL, superiority testing suggested a borderline significant advantage of DP-SES over DP-EES, with no significant differences between groups on other measures of angiographic performance.
- The clinical efficacy of DP-EES appeared similar to the DP-SES, but there was an improved safety due to a lower risk of stent thrombosis with DP-EES.

This study represents the first randomized comparison between DES among female patients. Potential mechanisms by which female sex could affect the outcome after PCI include different clinical and angiographic characteristics and a different distribution of cardiovascular risk factors. [16, 17]

Safety and efficacy profiles of DES were established largely independently of gender.[11, 18] However, evidence of the comparative performance of early- and new- generation DES is sparse and based on retrospective or post hoc analyses. In the female cohort of the SORT OUT IV trial (Randomized Clinical Comparison of the Xience V and the Cypher Coronary Stents in Non-selected Patients With Coronary Heart Disease), DP-EES had similar rates of major adverse cardiac events compared with DP-SES at 18 months (6.3% vs 7.9%, respectively, p = 0 .47).[19] In contrast, a retrospective registry including 1,649 women reported a significantly lower rate of major adverse cardiac events in DP-EES compared with DP-SES at 1-year (10.8% vs 14.7%, p = 0.04).[20] Against this background, the present study provides the first randomized evidence of angiographic performance of DP-EES compared with DP-SES in female patients. Our results corroborate the findings of previous studies not restricted to female patients. In the EXCELLENT (Efficacy of Xience/Promus Versus Cypher to Reduce Late Loss After Stenting) trial, DP-EES were formally found non inferior to DP-SES in terms of 9-month in-segment and in-stent LLL (0.11±0.38 mm vs. 0.06±0.36 mm, p for non-inferiority = 0.0382 and 0.19±0.35 mm vs. 0.15±0.34 mm, p for non-inferiority = 0.012, respectively), with trends towards a slightly higher LLL with DP-EES, concordant with our trial.[21] Similarly, the angiographic sub-study of the RESET trial (Randomized Evaluation of Sirolimus-eluting versus Everolimus eluting stent Trial) enrolling 571 patients demonstrated the non-inferiority of DP-EES relative to DP-SES in terms of in-segment LLL (0.06±0.37 mm vs. 0.02±0.46 mm, p for non-inferiority<0.001), again numerically with a slightly higher LLL for DP-EES.[22] **Table 4**lists other randomized trials that reported somewhat higher LLL with second-generation DES as compared with DP-SES.[21, 23, 24] These angiographic results may not reflect a lower angiographic efficacy of second generation DES, a decreased tendency for pathological remodeling and an increased propensity to have a small, yet homogeneous neointimal layer across the entire stent surface, which is likely to be protective against thrombotic processes.[25] The notion that lesser angiographic LLL is always associated with better clinical outcome, as found for bare-metal stents and early generation DES,[26] may therefore hold true no longer. Angiographic data for second generation DES contrast with the wealth of clinical data supporting an improved safety and efficacy of second generation DES over DP-SES.[27] Intravascular imaging has the potential to improve our understanding of vascular response to stent implantation. In this regard, optical coherence tomography-based studies showed that DP-EES have the best performance in terms of struts apposition among other early- and new-generation DES.[28]

**Table 4:** Angiographic performance of DP-SES and second generation DES across randomized studies.

		Late Lumen Loss		
Study (Author, year)	Nr. of Patients	Second generation DES	DP-SES	p value
**EXCELLENT (Park, 2011)**	DP-EES: 708	0.19±0.35	0.15± 0.34	0.09
	DP-SES: 216			
**LONG-DES-III (Park, 2011)**	DP-EES: 224	0.17±0.41*	0.09±0.30*	0.042
	DP-SES: 226			
**DiabeDES IV [23]**	DP-EES: 79	0.13±0.57**	-0.05±0.62**	0.06
	DP-SES: 77			
**ENDEAVOR III (Kandzari, 2006)**	DP-ZES: 323	0.34±0.44	0.13±0.32	< 0.001
	DP-SES: 113			
**ISAR-TEST-2 (Byrne, 2010)**	DP-ZES: 339	0.58 ± 0.55	0.24±0.51	< 0.001
	DP-SES: 335			

Number of first events and percentages are reported. Rate ratios RR (95% CI) are estimated using the Mantel-Cox method with two-sided p-values from log-rank test. All events were censored beyond 365 days. Continuity corrected RR with Fisher's exact test for zero outcomes.

*Cardiac death, TV-MI, clinically indicated TLR

**Primary endpoint.

MI, Myocardial infarction; TIA, Transient ischemic attack; TLR, Target lesion revascularization; TVR, Target vessel revascularization.

Even though the DP-SES is no longer used in clinical practice, results of our trial are still scientifically and clinically useful in that they can inform future network meta-analyses of both angiographic and clinical outcomes in women with stable coronary artery disease. Such analyses will typically include comparisons with first generation DP-DES to achieve best statistical precision and allow careful exploration of inconsistency.[27]

A lower rate of definite stent thrombosis as well numerically lower definite or probable stent thrombosis events were reported in the DP-EES arm of our study. These observations are in line with available randomized evidence. The SORT-OUT IV trial reported a significant reduction in the risk of overall and very late stent thrombosis in patients assigned to DP-EES compared with DP-SES at 3 years follow-up.[29] In a meta-analysis of 11 randomized trials, Park and colleagues found a 54% relative reduction in the risk of definite stent thrombosis and a 15% relative reduction in the risk of repeat revascularization among 12,869 patients treated with DP-EES compared with DP-SES.[30] Moreover, network meta-analyses reported a lower risk of stent thrombosis with the DP-EES compared with BMS and early-generation DES.[3, 31–33] In a pooled analysis including more than 11,000 female participants across 26 randomized trials, new-generation DES were associated with a significant reduction in the risk of stent thrombosis and TLR compared with early-generation DES during the 1-year of follow-up.[11]

The findings of the present study have to be interpreted in view of several limitations. 1.The comparison between new and early generation DES could be considered outdated as the Cypher DP-SES is no longer commercially available for clinical use. This limitation suggests that despite dedicated research efforts, appropriately designed trials in women occur at a later stage of clinical research. 2. There was a significant delay between the end of the trial in May 2012 and its publication, which is explained by delays in execution of contracts for the data analysis, which was outsourced by the sponsor to an academic clinical trials unit, two statisticians sequentially leaving the unit unexpectedly for new positions before completing the analyses, and a series of rejections of the submitted manuscript in view of the outdated nature of the control device and the ambiguous findings of the trial. 3. The study was not powered to identify differences in clinical outcomes since the sample size was estimated on the basis of the angiographic primary endpoint. 4. Patients with acute coronary syndromes were excluded from the trial, and therefore our results may not be extrapolated to this patient population. 5. A drop-out rate of approximately 30% for angiographic follow-up is relatively high compared to other trials with primary angiographic outcome. 6. The rates of TLR were somewhat increased compared with other randomized studies. Although female patients have generally a higher risk profile, the angiographic follow-up may inflate the rates new revascularization procedures. However, the rate of clinically–indicated TLR is in line with previous trials investigating the performance of DES in female patients. 7. Further iterations of the delivery system of the Xience DP-EES were done in the last years (Xience Xpedition, XIENCE Alpine), even though stent, drug coating formulation and drug dose density remained identical.

## Conclusions

In the first randomized trial comparing two different DES among female patients undergoing PCI, DP-EES was associated with a small increase in in-stent LLL at 9 months as compared to DP-SES, but a lower risk of definite stent thrombosis at 12 months.

## Supporting information

S1 CONSORT Checklist (DOC)

S1 Table List of sites participating into the SPIRIT WOMEN trial. (DOCX)

S2 Table Baseline clinical characteristics of patients receiving angiographic follow-up. (DOCX)

S3 Table Cumulative incidence of stent thrombosis up to 1 year follow-up. (DOCX)

S4 Table Baseline lesion and procedural characteristics. (DOCX)

S1 Fig Cumulative proportion of patients on dual antiplatelet therapy through 1-year follow-up in the DP-EES (blue line) and DP-SES (red line) arm. DAPT, Dual antiplatelet therapy; DP-EES, Durable polymer-everolimus eluting stent; DP-SES, Durable polymer-sirolimus eluting stent.
(TIF)

S2 Fig **Kaplan–Meier cumulative event curves for the composite of death, myocardial infarction, and target-vessel revascularization [TVR]) throughout 1 year (A), any death (B), myocardial infarction (C), and target-lesion revascularization [TLR] (D) for patients receiving DP-EES [blue lines] and DP-SES [red lines].** DP-EES, Durable polymer-everolimus eluting stent; DP-SES, Durable polymer-sirolimus eluting stent; FUP, Follow-up; ITT, Intention to treat; MI, Myocardial infarction; RR, Risk Ratio; TVR, Target vessel revascularization.
(TIF)

S1 Study Protocol Complete protocol of the SPIRIT WOMEN trial. (PDF)
