# Supplementary material for: A randomized multicenter trial comparing the XIENCE everolimus eluting stent with the CYPHER sirolimus eluting stent in the treatment of female patients with de novo coronary artery lesions: The SPIRIT WOMEN study
Source: PLoS One. 2017 Aug 10;12(8):e0182632. doi: 10.1371/journal.pone.0182632 (PMC5552121; doi:10.1371/journal.pone.0182632)
Supplement: S1 Table — (DOCX) [file pone.0182632.s002.docx]

| **Supplemental Table 1. List of Ethic Committees of Sites participating into the SPIRIT WOMEN trial** | | | |
| --- | --- | --- | --- |
| **Country** | **IRB Name** | **Address** | **City** |
| Argentina | Comité de Etica de Protocolos de Investigación | Gascón 450 1° Piso | Capital Federal |
| Argentina | Comité de Etica-Instituto Cardiovascular de Buenos Aires | Blanco Encalada 1543- C1418 DCO | Capital Federal |
| Austria | Ethikkommission der Medizinischen Universität Wien | Borschkegasse 8b/E06 | Wien |
| Belgium | Commissie Medische Ethiek UZ | Laarbeeklaan 101 | Brussels |
| Brazil | CEP do Instituto Dante Pazzanese de  Cardiologia | Av. Dr. Dante Pazzanese, 500 | São Paulo |
| Denmark | The Scientific-Ethical Committe C for  The Capital Region of Denmark | Kongens Vænge 2 | Hillerød |
| France | CPP Ile de France 3 | 89 rue d'Assas | Paris |
| Germany | EC LÄK Schleswig  Holstein | Bismarckallee 8-12 | Bad Segeberg |
| Germany | EC d. med. Fakultät d.  Ruhr-Uni  Bochum Sitz Bad Oey | Georgstrasse 11 | Bad  Oeynhausen |
| Germany | Ethikk. der  medizinischen Fakultät  der TU Dresden | Fetscherstrasse 74 | Dresden |
| Hungary | ETT TUKEB | Arany J. u.6-8. | Budapest |
| Italy | Comitato Etico ASL Città di Milano | Via statuto, 5 | Milan |
| Italy | Comitato Etico Provinciale di Modena | Via del Pozzo, 71 | Modena |
| Italy | CE ASL Mi 2 | Via VIII Giugno, 69 | Milan |
| Italy | CE Azienda Ospedaliero Universitaria  Pisana | VIA ROMA, 67 | Pisa |
| Italy | Comitato Etico del  Centro  Cardiologico Monzino | Via Carlo Parea 4 | Milan |
| Latvia | Ethics Committee for  Clinical Research of Medicines | Paula Stradina  Clinical  University Hospital | Riga |
| The Netherlands | Verenigde  commissies  mensgebonden onderzoek | Koekoekslaan 1 | EM Nieuwegein |
| The Netherlands | Medisch Ethische Commissie AMC | Meibergdreef 9 | Amsterdam |
| Norway | Regional komite for med. forskningsetikk | Det medisinske fakultet, Universitetet i Bergen | Bergen |
| Poland | Komisja Bioetyczna przy Instytucie  Kardiologii | Piętro II | Warsaw |
| Spain | EC of Clinical Trials of  Hospital  Germans Trias i Pujol | Carretera de Can Ruti Camí de les ecoles, s/n,  Campus Can Ruti, | Barcelona |
| Spain | EC of Clinical Trials of H. General Univ. de Alicante | Maestro Alonso 109 | Alicante |
| Switzerland | Comitato Etico Cantonale | Via Orico 5 | Bellinzona |
| Switzerland | Kantonale Ethikkommission Bern | Postfach 56 | Bern |
