# Supplementary material for: A randomized multicenter trial comparing the XIENCE everolimus eluting stent with the CYPHER sirolimus eluting stent in the treatment of female patients with de novo coronary artery lesions: The SPIRIT WOMEN study
Source: PLoS One. 2017 Aug 10;12(8):e0182632. doi: 10.1371/journal.pone.0182632 (PMC5552121; doi:10.1371/journal.pone.0182632)
Supplement: S2 Table — (DOCX) [file pone.0182632.s003.docx]

| **Supplemental Table 2. Baseline lesion and procedural characteristics** | | | | |
| --- | --- | --- | --- | --- |
|  |  |  |  |  |
|  | **Total** | **DP-EES** | **DP-SES** | **p value** |
| N. Patients | 455 | 304 | 151 |  |
| N. Lesions | 623 | 423 | 200 |  |
| ***Target lesion location*** |  |  |  | 0.12 |
| LM | 5 (0.8%) | 3 (0.7%) | 2 (1%) |  |
| LAD | 278 (44.6%) | 176 (41.7%) | 102 (51.%) |  |
| LCX | 158 (25.4%) | 109 (25.8%) | 49 (24.5%) |  |
| RCA | 181 (29.1%) | 134 (31.7%) | 47 (23.5%) |  |
| ***Type of stent implanted*** |  |  |  |  |
| No stent implanted | 5 (0.8%) | 3 (0.7%) | 2 (1%) |  |
| DP-EES | 402 (64.6%) | 397 (94.0%) | 5 (2.5%) |  |
| DP-SES | 194 (31.1%) | 2 (0.4%) | 192 (96%) |  |
| Multiple stent-types per patient | 12 (1.9%) | 11 (2.%) | 1 (0.5%) |  |
| Three-vessel intervention | 65 (10.4%) | 57 (13.4%) | 8 (4.0%) | 0.04 |
| Complex lesions | 314 (50.4%) | 217 (51.3%) | 97 (48.5%) | 0.61 |
| Lesion length (mm) | 15.34 ± 8.27 | 15.08 ± 8.74 | 15.86 ± 7.21 | 0.30 |
| Lesion length > 20 mm | 48 (8.2%) | 34 (8.5%) | 14 (7.6%) |  |
| RVD (mm) | 2.58 ± 0.46 | 2.58 ± 0.45 | 2.58 ± 0.47 | 0.83 |
| MLD (mm) | 1.01 ± 0.43 | 0.99 ± 0.42 | 1.04 ± 0.44 | 0.16 |
| Percent diameter stenosis (%) | 59.92 ± 12.11 | 60.62 ± 11.94 | 58.46 ± 12.35 | 0.06 |
| Calcification | 44 (7.0%) | 31 (7.3%) | 13 (6.5%) | 0.71 |
| ***Type of lesion*** |  |  |  | 0.64 |
| A | 77 (13.5%) | 55 (14.2%) | 22 (12.1%) |  |
| B1 | 209 (36.8%) | 136 (35.2%) | 73 (40.3%) |  |
| B2 | 234 (41.2%) | 162 (41.9%) | 72 (39.7%) |  |
| C | 47 (8.2%) | 33 (8.5%) | 14 (7.7%) |  |
|  |  |  |  |  |
| Data expressed as n (%) or means ± standard deviations. Variables are shown at lesion level Two-sided p-values from linear mixed models with random intercepts at level of patients (continuous outcomes) or from multinomial logistic regression with cluster-robust variance estimation (categorical outcomes). * Patient-level analysis. LAD, Left anterior descending; LCX, Left circumflex; LM, Left main; MLD, Minimal luminal diameter; RCA, Right coronary artery; RVD, Reference vessel diameter. | | | | |
