# Supplementary material for: A randomized multicenter trial comparing the XIENCE everolimus eluting stent with the CYPHER sirolimus eluting stent in the treatment of female patients with de novo coronary artery lesions: The SPIRIT WOMEN study
Source: PLoS One. 2017 Aug 10;12(8):e0182632. doi: 10.1371/journal.pone.0182632 (PMC5552121; doi:10.1371/journal.pone.0182632)
Supplement: S3 Table — (DOCX) [file pone.0182632.s004.docx]

| **Supplemental Table 3. Baseline clinical characteristics of patients receiving angiographic follow-up** | | |  |
| --- | --- | --- | --- |
|  |  |  |  |
|  | **DP-EES**  **n= 219** | **DP-SES**  **n= 110** | **p-value** |
|  |  |  |  |
| Age at randomization | 67.8 ± 9.9 | 68.3 ± 11.0 | 0.68 |
| Waist circumference (cm) | 94.3 ± 15.6 | 94.5 ± 17.3 | 0.93 |
| Body mass index (kg/m^2^) | 27.8 ± 5.3 | 28.0 ± 5.4 | 0.84 |
| ***Cardiac risk factors*** |  |  |  |
| Current tobacco user | 43 (20%) | 22 (20.6%) | 1.00 |
| Postmenopausal status | 206 (94.5%) | 103 (93.6%) | 0.80 |
| Hypertension requiring medication | 180 (82.2%) | 93 (84.5%) | 0.64 |
| Lipid disorder requiring medication | 164 (74.9%) | 82 (75.2%) | 1.00 |
| Diabetes Type II | 60 (27.4%) | 29 (26.4%) | 0.90 |
| Oral hypoglycemics | 37 (61.7%) | 17 (58.6%) | 0.82 |
| Insulin | 16 (26.7%) | 5 (17.2%) | 0.43 |
| Previous coronary intervention | 41 (18.7%) | 22 (20.2%) | 0.77 |
| Previous MI | 53 (24.3%) | 19 (17.4%) | 0.20 |
| MI within 2 months of screening date | 17 (32.1%) | 6 (31.6%) | 1.00 |
| Family history of premature CAD | 81 (37.0%) | 41 (37.3%) | 1.00 |
| ***Clinical presentation*** |  |  |  |
| Current anginal status |  |  |  |
| Stable angina | 106 (48.4%) | 60 (54.5%) | 0.35 |
| Unstable angina | 86 (39.3%) | 35 (31.8%) | 0.23 |
| Silent ischemia | 27 (12.3%) | 15 (13.6%) | 0.73 |
| Left ventricular dysfunction | 28 (14.2%) | 14 (14.9%) | 0.86 |
| Anginal symptoms |  |  |  |
| Typical angina | 168 (77.1%) | 90 (81.8%) | 0.39 |
| Atypical chest pain | 23 (10.6%) | 8 (7.3%) | 0.43 |
| No chest pain | 27 (12.4%) | 12 (10.9%) | 0.86 |
|  |  |  |  |
| Data expressed as n (%) or means ± standard deviations.  CAD, Coronary artery disease; DP-EES, Durable polymer- everolimus eluting stents; DP-SES, Durable polymer- sirolimus eluting stents; MI, Myocardial infarction. | | |  |
