# Supplementary material for: A randomized multicenter trial comparing the XIENCE everolimus eluting stent with the CYPHER sirolimus eluting stent in the treatment of female patients with de novo coronary artery lesions: The SPIRIT WOMEN study
Source: PLoS One. 2017 Aug 10;12(8):e0182632. doi: 10.1371/journal.pone.0182632 (PMC5552121; doi:10.1371/journal.pone.0182632)
Supplement: S4 Table — (DOCX) [file pone.0182632.s005.docx]

| **Supplemental Table 4. Cumulative incidence of stent thrombosis up to 1 year follow-up** | | | |
| --- | --- | --- | --- |
|  |  |  |  |
|  | **DP-EES**  **n= 304** | **DP-SES**  **n= 151** | **p-value** |
|  |  |  |  |
| **Definite ST** | 0 (0.0%) | 3 (2.0%) | 0.036 |
| Acute | 0 (0.0%) | 1 (0.7%) | 0.32 |
| Subacute | 0 (0.0%) | 1 (0.7%) | 0.33 |
| Late | 0 (0.0%) | 1 (0.7%) | 0.33 |
| **Probable ST** | 0 (0.0%) | 0 (0.0%) | . |
| Acute | 0 (0.0%) | 0 (0.0%) | . |
| Subacute | 0 (0.0%) | 0 (0.0%) | . |
| Late | 0 (0.0%) | 0 (0.0%) | . |
| **Possible ST** | 1 (0.3%) | 1 (0.7%) | 0.55 |
| Acute | 0 (0.0%) | 0 (0.0%) | . |
| Subacute | 0 (0.0%) | 0 (0.0%) | . |
| Late | 1 (0.3%) | 1 (0.7%) | 0.55 |
|  |  |  |  |
| Number of first events and percentages are reported. P-values from Fisher's exact test. DP-EES, Durable polymer- everolimus eluting stents; DP-SES, Durable polymer- sirolimus eluting stents; ST, Stent thrombosis. | | | |
