# Supplementary figures and images for: A randomized multicenter trial comparing the XIENCE everolimus eluting stent with the CYPHER sirolimus eluting stent in the treatment of female patients with de novo coronary artery lesions: The SPIRIT WOMEN study
Source: PLoS One. 2017 Aug 10;12(8):e0182632. doi: 10.1371/journal.pone.0182632 (PMC5552121; doi:10.1371/journal.pone.0182632)

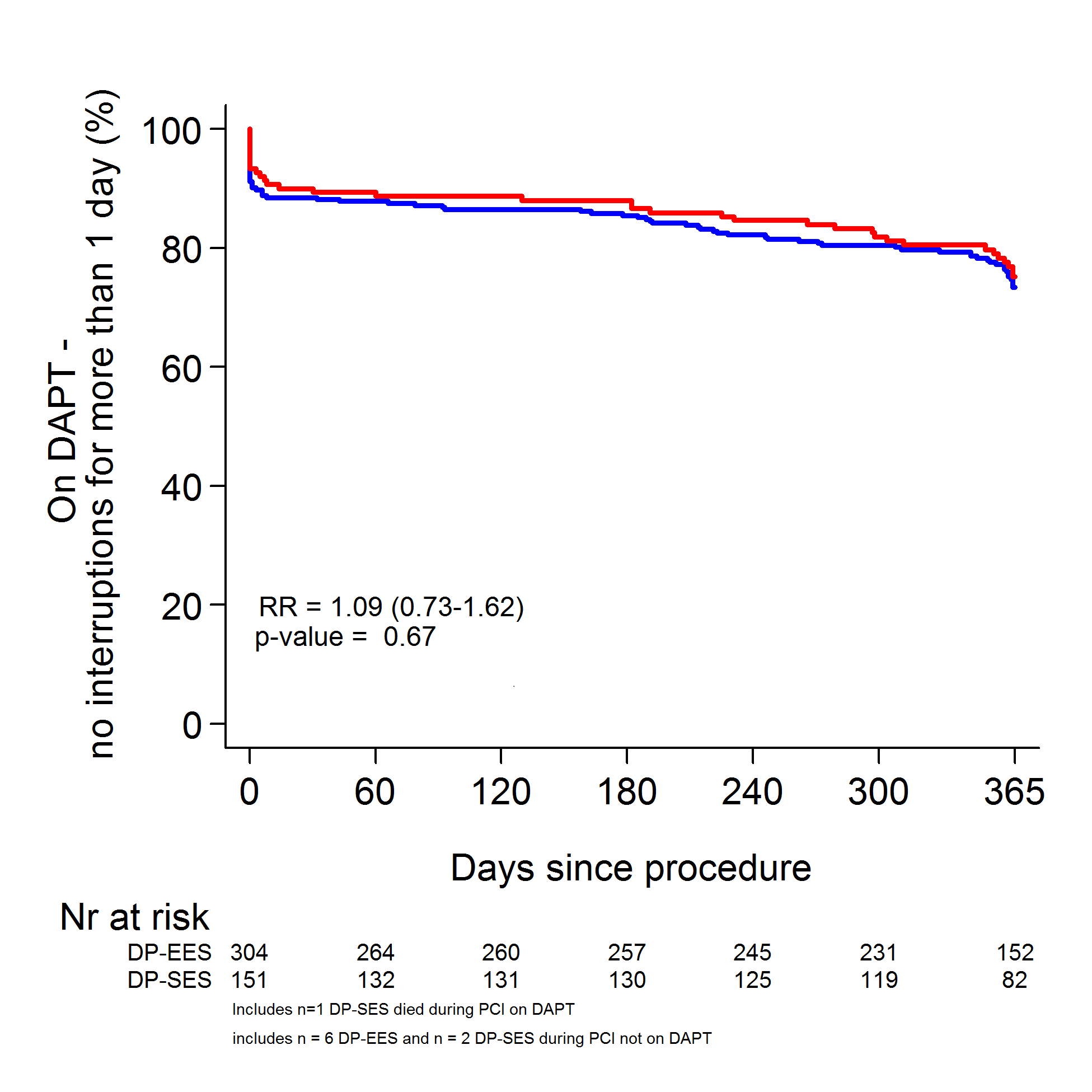

Supplement: S1 Fig — DAPT, Dual antiplatelet therapy; DP-EES, Durable polymer-everolimus eluting stent; DP-SES, Durable polymer-sirolimus eluting stent. (TIF) [file pone.0182632.s006.tif]

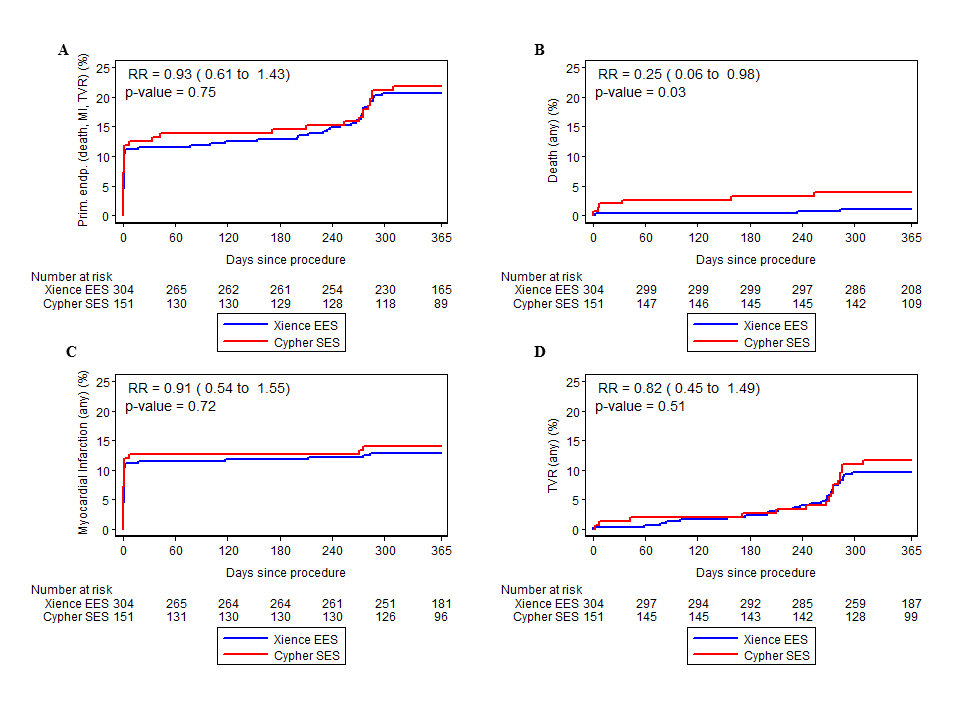

Supplement: S2 Fig — Kaplan–Meier cumulative event curves for the composite of death, myocardial infarction, and target-vessel revascularization [TVR]) throughout 1 year (A), any death (B), myocardial infarction (C), and target-lesion revascularization [TLR] (D) for patients receiving DP-EES [blue lines] and DP-SES [red lines]. DP-EES, Durable polymer-everolimus eluting stent; DP-SES, Durable polymer-sirolimus eluting stent; FUP, Follow-up; ITT, Intention to treat; MI, Myocardial infarction; RR, Risk Ratio; TVR, Target vessel revascularization. (TIF) [file pone.0182632.s007.tif]
