# Supplementary material for: A randomized multicenter trial comparing the XIENCE everolimus eluting stent with the CYPHER sirolimus eluting stent in the treatment of female patients with de novo coronary artery lesions: The SPIRIT WOMEN study
Source: PLoS One. 2017 Aug 10;12(8):e0182632. doi: 10.1371/journal.pone.0182632 (PMC5552121; doi:10.1371/journal.pone.0182632)
Supplement: S1 Study Protocol — (PDF) [file pone.0182632.s008.pdf]

Confidential and Proprietary
Nothing herein is to be disclosed without the expressed written consent of the Sponsor

CLINICAL INVESTIGATION PLAN 07-377

SPIRIT WOMEN Clinical Evaluation

A Clinical Evaluation of the XIENCE Everolimus Eluting Coronary Stent System in the Treatment of Women with *de novo* Coronary Artery Lesions (Combined Study Protocol)

Version Number:	Version 4.0
Date:	25 August 2009
Principal Investigator:	Dr. Marie-Claude Morice
Co Principal Investigator:	Prof. Stephan Windecker
Sponsor:	Abbott Cardiovascular Systems, Inc.<br>Affiliate of Abbott Vascular Inc ("Sponsor")<br>3200 Lakeside Drive<br>Santa Clara, California 95054<br>USA
Authorized Representative in the EU:	Abbott Vascular International BVBA<br>Park Lane<br>2B Culliganlaan<br>1831 Diegem (Belgium)<br>BE 0886.537.933
Study Monitoring:	Quintiles Limited,<br>Station House, Market Street, Bracknell,<br>Berkshire<br>RG12 1HX United Kingdom
Central Laboratory:	ICON Central Laboratories<br>South County Business Park<br>Leopardstown, Dublin 18, Ireland
Contact:	Bridget Hurley<br>Director, Clinical Operations<br>Abbott Vascular International BVBA<br>Park Lane - Culliganlaan 2B<br>1831 Diegem, Belgium<br>Tel: +32 2 416 9223<br>Fax:+ 32 2 416 9101
Steering Committee:	Prof. Maria Grazia Modena<br>Dr. Ghada Mikhail<br>Dr. Fina Mauri i Ferré<br>Prof. Ruth Strasser<br>Dr. Liliana Grinfeld

Amendment to Version 1.0 Dated 19 March 2007

The purpose of this amendment to the SPIRIT Women Clinical Investigational Plan (CIP) is to provide general clarification and updated information regarding the study. The major modifications included in the amendment are as follows:

The name of the study has been changed to XIENCE V SPIRIT Women.

FDA approval of the XIENCE V EECSS on 02 July 2008 has been added as appropriate.

Mandated treatment strategy has been modified. The treatment strategy will be determined by the Investigator; however it is recommended that each enrolling Investigator review the most recent IFU to assess contraindications, warnings, and precautions for treating potential patients. This is to allow a more pragmatic approach to the treatment of patients enrolled in the study.

Data regarding the Spirit ll and Spirit lll studies have been updated with the most current information.

Clarification of the Intent to Treat (ITT) analysis for the Single Arm Study has been provided.

Adverse event collection following the 2 year follow up visit will be limited to cardiac adverse events to better reflect the focus of the study,

Blood work requirements have been updated to ensure alignment with the published ARC definitions.

Study endpoint definitions have been updated to ensure alignment with the published ARC definitions.

Removal of Unanticipated Adverse Device Effect reporting as not required for non-FDA studies.

Clarification provided for the timing of Adverse Event reporting requirements.

Changes in study administration have been updated.

Amendment to Version 2.0 Dated 19 August 2008

The major modifications included in the amendment are as follows:

Adverse event collection following the 2 year follow up visit will be limited to serious adverse events, cardiac adverse events and device related adverse events to better reflect the focus of the study,

Clarifications in the Informed Consent Process.

Clarification in the Screening Process

Updated Declaration of Helsinki

Amendment to Version 3.0 Dated 25 August 2009

The XIENCE PRIME Everolimus Eluting Coronary Stent System (EECSS) received CE Mark in June 2009. The CE Mark for the XIENCE PRIME EECSS was obtained based upon the consideration that the clinical data of the SPIRIT trials are also applicable to the XIENCE PRIME EECSS. The SPIRIT trials have established the safety and performance of the XIENCE V EECSS and therefore no additional clinical study data were required for the XIENCE PRIME EECSS.

The purpose of this amendment is to allow the use of both XIENCE Everolimus Eluting Coronary Stent Systems (XIENCE V and XIENCE PRIME EECSS) in the XIENCE V SPIRIT WOMEN Randomized Sub-study.

The major modifications included in the amendment are as follows:

The name of the study has been changed to SPIRIT Women.

XIENCE V EECCS has been replaced with XIENCE EECCS (XIENCE V EECCS and XIENCE PRIME EECCS)

The device description has been added for the XIENCE PRIME EECSS.

Data regarding the Spirit V study has been updated with the most current information.

Revision History can be found in Appendix VIII.

TABLE OF CONTENTS

1 INTRODUCTION 11

2 BACKGROUND INFORMATION 11

2.1 BACKGROUND AND RATIONALE 11
2.1.1 Literature Review 11
2.2 DESCRIPTION OF XIENCE V EECSS 13
2.2.1 The XIENCE V EECSS Delivery System 14
2.2.2 The XIENCE V EECSS Polymer Coating 15
2.2.3 The Anti-proliferative Drug: Everolimus 15
2.3 DESCRIPTION OF XIENCE PRIME EECSS 15
2.4 DESCRIPTION OF THE CYPHER SELECT™ PLUS 16

3 CLINICAL EVALUATION OBJECTIVE 16

4 CLINICAL EVALUATION DESIGN 16

4.1 PATIENT FOLLOW-UP 16
4.2 TREATMENT STRATEGY 17
4.3 MEASURES TO BE TAKEN TO AVOID/MINIMIZE BIAS 18

5 ENDPOINTS 18

5.1 PRIMARY ENDPOINT 18
5.2 SECONDARY ENDPOINTS 18

6 SELECTION AND WITHDRAWAL OF PATIENTS 19

6.1 PATIENT POPULATION 19
6.2 PATIENT SCREENING 19
6.2.1 Informed Consent 19
6.2.2 Ethical Considerations 19
6.3 ALLOCATION OF PATIENTS TO THE SPIRIT WOMEN SINGLE ARM STUDY AND RANDOMIZED SUB-STUDY 20
6.4 ELIGIBILITY CRITERIA 20
6.4.1 General Inclusion Criteria 20
6.4.2 Angiographic Inclusion Criteria 20
6.4.3 General Exclusion Criteria 21
6.5 PATIENT DISCONTINUATION 21

7 TREATMENT OF PATIENTS 22

7.1 PRE-PROCEDURE (BASELINE) 22
7.1.1 Patient History 22
7.1.2 Laboratory Assessments 22
7.1.3 Other Assessments 23
7.1.4 Medications 23
7.2 ENROLLMENT 23
7.3 PROCEDURE 24
7.3.1 Medications 24
7.3.2 Baseline Angiography 24
7.3.3 Stenting Procedure 24

7.3.3.1 SPIRIT WOMEN Single Arm Study 24
7.3.3.2 Randomized Sub-study 24
7.3.4 Treatment Rules for the XIENCE EECS *25*
7.3.5 Angiography Following Stent Placement *25*
7.3.6 Bailout Procedures *25*
7.4 POST-PROCEDURAL TESTING AND MEDICATIONS 25
7.4.1 ECG *25*
7.4.2 Cardiac Enzymes and Creatinine *25*
7.4.2.1 XIENCE V SPIRIT WOMEN Single Arm Study (Local Laboratory) 26
7.4.2.2 Randomized Sub-study (Central Laboratory) 26
7.4.3 Medications *26*
7.4.3.1 SPIRIT WOMEN Single Arm Study 26
7.4.3.2 Randomized Sub-study 26

8 ASSESSMENT OF SAFETY AND PERFORMANCE 27

8.1 CLINICAL FOLLOW-UP FOR ALL PATIENTS 27
8.1.1 Additional Follow-up Visits for All Patients *27*
8.2 ANGIOGRAPHIC FOLLOW-UP FOR RANDOMIZED SUB-STUDY 28
8.3 ADVERSE EVENTS 29
8.3.1 Definitions *29*
8.3.2 Adverse Event Reporting *30*
8.4 SERIOUS ADVERSE EVENTS 31
8.5 SAFETY MONITORING 31
8.5.1 Data Safety Monitoring Board (DSMB) *31*
8.6 ADJUDICATION OF EVENTS 31
8.6.1 Clinical Events Committee (CEC) *31*

9 STATISTICAL ANALYSIS 32

9.1 STATISTICAL OVERVIEW 32
9.2 SAMPLE SIZE JUSTIFICATION 33
9.3 ANALYSIS POPULATIONS 33
9.3.1 Intent to Treat Population *33*
9.3.2 Per-Treatment Evaluable Population *34*
9.4 STATISTICAL ANALYSES 34
9.5 CRITERIA FOR EARLY TERMINATION OF THE TRIAL FOR EFFICACY 35
9.6 PROCEDURES FOR ACCOUNTING FOR MISSING DATA 35
9.7 DOCUMENTATION OF CHANGES TO STATISTICAL PLAN 35

10 DIRECT ACCESS TO SOURCE DATA/DOCUMENTS 35

11 QUALITY CONTROL AND QUALITY ASSURANCE 36

11.1 SELECTION OF CLINICAL SITES AND INVESTIGATORS 36
11.2 MEC APPROVAL OF CLINICAL INVESTIGATION PLAN AND INFORMED CONSENT 36
11.2.1 Clinical Investigation Plan Amendments *36*
11.2.2 Clinical Investigation Plan Deviations *36*
11.2.3 Premature Termination of the Clinical Evaluation *37*
11.2.4 Control of Study Devices and Equipment *37*
11.3 TRAINING 37
11.3.1 Site Training *37*

11.3.2 Compliance to Standards and Regulations ... *37*
11.3.3 Monitoring ... *37*
11.3.4 Designated Monitors ... *37*
11.3.5 Visits ... *38*
11.3.6 Securing Compliance ... *38*
11.3.7 Quality Assurance Audit ... *39*
11.4 THE STEERING COMMITTEE ... 39

12 RISK ANALYSIS ... 39

12.1 POTENTIAL RISKS (RISKS FROM CARDIAC CATHETER, STENTING AND PERCUTANEOUS TRANSCATHETER CORONARY ANGIOPLASTY) ... 39
12.2 ASSOCIATED RISKS OF EVEROLIMUS ... 40
12.3 RISK MANAGEMENT PROCEDURES ... 40
12.4 POTENTIAL BENEFITS ... 41

13 DATA HANDLING AND RECORD KEEPING ... 41

13.1 SOURCE DOCUMENTATION ... 42
13.2 CASE REPORT FORM COMPLETION ... 42
13.3 RECORD RETENTION ... 42

14 PUBLICATION POLICY ... 43

15 APPENDIX 1: DECLARATION OF HELSINKI ... 44

16 APPENDIX II DEFINITIONS ... 49

17 APPENDIX III ACRONYMS AND ABBREVIATION ... 60

18 APPENDIX IV STUDY FLOW / FOLLOW-UP SCHEDULE ... 62

19 APPENDIX V SCHEDULE OF EVENTS ... 63

20 APPENDIX VI SCREENING PROCESS ... 64

21 APPENDIX VII CLINICAL INVESTIGATION CONTACTS ... 66

22 APPENDIX VIII REVISION HISTORY SPIRIT WOMEN PROTOCOL ... 67

23 APPENDIX IX REFERENCES ... 102

CLINICAL INVESTIGATION PLAN SUMMARY

**Trial Name and Number**	SPIRIT WOMEN Clinical Evaluation: 07-377
**Title**	A Clinical Evaluation of the XIENCE Everolimus Eluting Coronary Stent System in the Treatment of Women with *de novo* Coronary Artery Lesions
**Objectives**	• Continued assessment of the XIENCE Everolimus Eluting Coronary Stent System (XIENCE V EECSS and XIENCE PRIME EECSS) with the primary focus on clinical outcomes in the treatment of female patients with de novo coronary artery lesions<br>• Characterization of the female population undergoing stent implantation with XIENCE EECSS
**Patient enrollment**	Approximately 2000 female patients derived from the general interventional cardiology population will be enrolled from up to 130 sites outside the United States.<br>SPIRIT WOMEN Clinical Evaluation patients are 'real world' patients, screened in accordance with the commercial Instruction for Use (IFU) of XIENCE..
**Clinical Investigation Design**	The SPIRIT WOMEN Clinical Evaluation is a prospective, open label, single arm, multi-center study evaluating performance of the XIENCE EECSS in the treatment of female patients with coronary artery lesions, per the Instructions for Use (IFU). Additionally, this study will include a prospective, single-blind, double arm, randomized multi-center sub-study comparing the XIENCE EECSS to the CYPHER SELECT™ Plus Sirolimus-eluting Coronary Stent in the treatment of female patients with coronary artery lesions.<br>• Approximately 1550 patients will be enrolled in the SPIRIT WOMEN single arm study<br>• Approximately 450 patients will be randomized 2:1 (XIENCE EECSS vs. CYPHER SELECT™ Plus Sirolimus-eluting Coronary Stent) from up to 35 selected sites (randomized sub-study).<br>The test devices used are the XIENCE V Everolimus Eluting Coronary Stent System (XIENCE V EECSS) and the XIENCE PRIME Everolimus Eluting Coronary Stent System (XIENCE PRIME EECSS (randomized sub-study only)). The XIENCE V EECSS has received CE mark approval on 30 January 2006 and FDA approval on 02 July 2008. The XIENCE PRIME EECSS has received CE mark on 23 June 2009.<br>The control device used in the randomized sub-study is CYPHER SELECT™ Plus Sirolimus-eluting Coronary Stent. In the event a subsequent generation of CYPHER SELECT™ Plus Sirolimus-eluting Coronary Stent is released during the course of the study, it may replace the CYPHER SELECT™ Plus Sirolimus-eluting Coronary Stent.<br>Evaluation of specific female demographics such as menopausal, hysterectomy and oophorectomy status, past and current use of hormonal contraceptives, and use of weak estrogens will occur for all patients.<br>Assessment of anginal status, collection of data regarding adverse events, details of any subsequent coronary interventions, CIP medications and use and changes in concomitant medications will be collected at 30 days, 240 days and 1, 2, 3, 4 and 5

	years post-procedure for all patients.<br>Angiographic follow-up at 270 days will be carried out in the randomized sub-study.
**Primary Endpoint**	Adjudicated Composite rate of all Death, all Myocardial Infarction (MI) and Target Vessel Revascularization (TVR) at 1 year
**Secondary Endpoints**	Acute Success (Clinical Device Success and Clinical Procedure Success)<br>Adjudicated Stent Thrombosis at 30 days, 240 days and at 1, 2, 3, 4 and 5 years (Definite, Probable, Possible)<br>Adjudicated revascularization (TLR/TVR/all revascularizations) at 30 days, 240 days and at 1, 2, 3, 4 and 5 years<br>Adjudicated Composite rate of Cardiac Death, MI attributed to the target vessel and CI-TLR at 30 days, 240 days and at 1, 2, 3, 4 and 5 years.<br>Adjudicated Composite rate of all Death, all MI and Target Vessel Revascularization (TVR) at 30 days, 240 days and at 2, 3, 4 and 5 years.<br>Adjudicated Composite rate of all Death, all MI and all Revascularization (TLR/TVR/non TVR) at 30 days, 240 days and at 1, 2, 3, 4 and 5 years.<br>Adjudicated Cardiac Death, Non-Cardiovascular Death, Vascular Death, Q-wave MI and Non Q-wave MI (Peri-Procedural, Unrelated to PCI) at 30 days, 240 days and at 1, 2, 3, 4 and 5 years.<br>In-stent Late Loss (LL) at 270 days (*main* secondary endpoint for the randomized sub-study)<br>In-segment Late Loss (LL) at 270 days (for the randomized sub-study)<br>In-stent and in-segment Angiographic Binary Restenosis rates at 270 days (for the randomized sub-study)<br>In-stent and in-segment percent Diameter Stenosis at 270 days (for the randomized sub-study)<br>Aneurysm, thrombus and persisting dissection at 270 days (for the randomized sub-study)
**Patient Follow-up**	Clinical F/U at 30 days, at 240 days and at 1, 2, 3, 4 and 5 years.<br>Angiographic F/U at 270 days for a subset of approximately 450 patients enrolled at selected sites (randomized sub-study).

**Treatment Strategy**	**All Patients:**<br>The treatment strategy will be determined by the investigator. It is recommended that each enrolling investigator review the most recent IFU to assess contraindications, warnings, and precautions for treating potential patients.<br><br>No non-study percutaneous procedures can be performed at the time of the study procedure.<br>**SPIRIT WOMEN Single Arm Study (SAS):**<br>After meeting the general and angiographic inclusion and exclusion criteria, the target lesion(s) should be treated by stenting with the XIENCE V EECSS according to the IFU<br>Registration should only be done after confirmation of the angiographic inclusion criteria and before implantation of first stent<br>All lesions should be planned to be treated with the XIENCE V<br>If any staged procedures are planned at baseline, it is necessary that each investigator treats the enrolled patients with only XIENCE V EECSS during the planned staged procedure.<br>If bailout stenting is necessary, a XIENCE V EECSS of appropriate length should be used<br>It is recommended that treatment with clopidogrel should be continued at 75 mg daily for a period of at least 6 months. Ticlopidine at a dose standard with hospital practice may be substituted for clopidogrel, as required by hospital practice or if the patient develops hypersensitivity to clopidogrel.<br>It is recommended that all patients should receive ≥ 75 mg of aspirin daily for a minimum of five years following the procedure<br>**Randomized Sub-study (RSS):**<br>The test devices used are the XIENCE V Everolimus Eluting Coronary Stent System (XIENCE V EECSS and the XIENCE PRIME Everolimus Eluting Coronary Stent System (XIENCE PRIME EECSS.<br>After meeting the general and angiographic inclusion and exclusion criteria, the target lesion(s) should be treated according to the same randomization schedule. If two or more target lesions are to be treated, all lesions must receive the treatment that has been assigned as per the randomization<br>If two or more target lesions are to be treated and the patient is randomized to the XIENCE EECSS, all lesions must receive either the XIENCE V EECSS or the XIENCE PRIME EECSS.<br>Randomization should be done only after confirmation of the angiographic inclusion criteria and before implantation of the first stent. Once randomization is completed and a treatment is assigned, crossover is not permitted.<br>If bailout stenting is necessary, the stent used should be the same as allocated by randomization for the index procedure, and should be used according to the Instructions for Use (IFU)<br>If any staged procedures are planned at baseline, it is necessary that each investigator treats the enrolled patients with the same study stent during the planned staged

	procedure as was used at baseline<br><br>All patients will receive clopidogrel at 75 mg daily for a period of at least 6 months. Ticlopidine at a dose standard with hospital practice may be substituted for clopidogrel, as required by hospital practice or if the patient develops hypersensitivity to clopidogrel.<br><br>All patients will receive ≥ 75 mg of aspirin daily for a minimum of five years following the procedure
**Key Inclusion Criteria**	• Females, at least 18 years of age and able to provide informed consent<br>• Patient must have evidence of myocardial ischemia<br>• Patient must be an acceptable candidate for coronary artery bypass graft (CABG) surgery<br>• Patient must agree to undergo all CIP-required follow-up examinations<br>• Patients' artery morphology and disease is suitable to be optimally treated with a maximum of 4 planned study stents<br>• Target lesions must be de novo lesions (no prior stent implant, no prior brachytherapy)<br>• Target vessel reference diameter must be between 2.5 mm and 4.0 mm by visual estimate. The diameter range will be expanded to 2.25 mm when the 2.25 mm stent is available.<br>• Target lesion ≤ 28 mm in length by visual estimate
**Key Exclusion Criteria**	• Participation in another device or drug study or has completed the follow-up phase of another study within the last 30 days<br>• Patient has had a previous stent implant, either BMS or DES within the target vessel(s)
**Primary Analytical Population**	Intent to treat population for all endpoints.

1 INTRODUCTION

The SPIRIT WOMEN Clinical Evaluation is a prospective, open label, single arm, multi-center study evaluating performance of the XIENCE Everolimus Eluting Coronary Stent System (XIENCE V EECSS) in the treatment of female patients with coronary artery lesions, per its Instructions for Use (IFU). Additionally, this study will include a prospective, single-blind, double arm, randomized multi-center sub-study comparing the XIENCE EECSS (XIENCE V EECSS and XIENCE PRIME EECSS) to the CYPHER SELECT™ Plus Sirolimus-eluting Coronary Stent in the treatment of female patients with coronary artery lesions.

- Approximately 1550 patients will be enrolled in the single arm study (SPIRIT WOMEN Single Arm Study / SAS).
- Approximately 450 patients will be randomized 2:1 (XIENCE EECSS vs. CYPHER SELECT™ Plus Sirolimus-eluting Coronary Stent) from up to 35 selected sites (randomized sub-study). In the event a subsequent generation of CYPHER SELECT™ Plus Sirolimus-eluting Coronary Stent is released during the course of the study, it may replace the CYPHER SELECT™ Plus Sirolimus-eluting Coronary Stent. (Randomized Sub-study / RSS)

The purpose of this clinical evaluation is to characterize the female population undergoing stent implantation and to continue the evaluation of the performance of XIENCE EECSS in this specific population in real-world use. Following initial stent implantation, all patients will have clinical follow-up at 30 days, 240 days and at 1, 2, 3, 4 and 5 years to assess the long term outcome of the stenting procedure. A sub group of patients (N = approximately 450) will have angiographic follow-up at 270 days (randomized sub-study).

2 BACKGROUND INFORMATION

2.1 Background and Rationale

2.1.1 Literature Review

Coronary heart disease has been shown to be the leading cause of death in Western countries with approximately equal incidence in men and women[i]. Due to increased life expectancy, particularly in women, the burden of the disease is increasing. General awareness is poor and women are likely to be diagnosed less often and at a later stage than men[ii, iii]. As this is also demonstrated by a smaller representation of women in clinical studies[iii], one of the recommendations of the Policy Conference on Cardiovascular Diseases in Women of the European Society of Cardiology was to conduct clinical trials enrolling only female patients[i].

Stenting of *de novo* lesions in native coronary arteries has been shown to be a safe and effective treatment of occlusion due to atherosclerosis[iv,v,vi,vii,viii,ix,x,xi,xii] and use of drug-eluting stents is currently the standard procedure in the USA and Europe.

Studies evaluating the local application of anti-proliferative drugs (e.g., sirolimus, paclitaxel derivatives) for prevention of restenosis via a stent delivery system show that these therapies successfully inhibit or reduce restenosis and associated clinical events[xiii,xiv,xv,xvi]. The local application of anti-proliferative drugs may decrease the need for patients to undergo repeat percutaneous transluminal coronary angioplasty (PTCA) or coronary artery bypass graft (CABG). It may also aid in decreasing the high cost of medical care involved when these repeat interventions are required. Everolimus, a sirolimus analogue, is a drug found to be an effective anti-proliferative agent. It inhibits growth factor-stimulated cell proliferation by causing cell cycle arrest in the late G1 stage in the cell cycle[xvii].

The feasibility of using everolimus on a drug eluting stent was demonstrated in the SPIRIT FIRST Study using the XIENCE V EECSS compared to the metallic, uncoated MULTI LINK VISION® RX Coronary Stent System (VISION® RX CSS) (N=60; N=28 for the everolimus cohort, and N=32 for the metallic stent cohort) in the treatment of patients with *de novo*, native coronary artery lesions. The primary endpoint, angiographic in-stent late loss, was observed at 0.10 mm for the XIENCE V EECSS, a reduction of 88% relative to the metallic control (p< 0.001). This was matched by excellent proximal and distal late loss values of 0.10 mm and 0.07 mm, respectively, which indicated no edge effects. These observations were consistent with IVUS measurements, which showed the 180-day in-stent % Volume Obstruction in the XIENCE V EECSS arm to be 8.0 % while the control arm value was 28.1%. This represented a reduction of neointimal volume of 72% relative to the metallic stent control.

The SPIRIT FIRST study showed clinical safety of the everolimus-eluting stent delivered from a durable polymer coating at 30 days and 180 days: No stent thrombosis events, no instances of late acquired stent malapposition and a six-month hierarchical MACE (Major Adverse Cardiac Event) rate of 7.7%, compared to a MACE rate of 21.4% for the VISION® RX CSS arm.

The SPIRIT FIRST one year and two year results demonstrate a continuation of the treatment effect of the XIENCE V EECSS observed at 6 months, and a reduced rate of cell proliferation compared to the uncoated control. At 1 year, the superiority in efficacy, as measured by in-stent late loss, of XIENCE V as compared to VISION® RX was sustained (71% reduction in late loss). The XIENCE V arm also maintained at 1 year its superiority to the VISION® RX arm in the secondary IVUS endpoints, % volume obstruction and neointimal volume (64% and 65% reduction respectively). The overall MACE rate was 15.4% in the XIENCE V arm and 21.4% in the VISION® RX arm. No late stent thrombosis events were observed in either group up to the 1 year follow-up time point. Two years follow-up demonstrated that the treatment effect that was observed at 1-year was sustained: there were no observations of late thrombotic events, and no additional Target Vessel Failures between 1 year and 2 years in the XIENCE V arm.

The SPIRIT II[xviii] Trial is a multi-center randomized controlled trial conducted in Europe to continue to assess the feasibility and performance of the XIENCE V EECSS in the treatment of subjects with a maximum of two de novo native coronary artery lesions each in a different epicardial vessel, compared to TAXUS® EXPRESS2 PECS. The trial enrolled 300 subjects at 28 sites; 223 subjects in the XIENCE V group, and 77 subjects in the TAXUS® group. Enrollment was completed in November, 2005. The SPIRIT II clinical trial includes clinical follow up at 30, 180, 270 days and 1- 5 years All patients were to have an angiographic follow-up at 180 days and a subset of 152 patients was to have an IVUS follow-up at 180 days and an angiographic and IVUS follow-up at 2 years.

The primary endpoint was met with an in-stent LL of 0.11 ± 0.27 mm for the XIENCE V group compared to 0.36 mm for TAXUS® PECSS at 180 days, which represents a 72% reduction in late loss. As such, XIENCE V EECSS clearly demonstrated both non-inferiority (p<0.0001) and superiority (p<0.0001) to the TAXUS® PECSS in terms of the primary endpoint of in-stent late loss. The IVUS results support the angiographic endpoint results. There was a 66% reduction in %VO in the XIENCE V group as compared to the TAXUS® group (2.5% vs. 7.4%) and a 73% reduction in NIH volume (3.8 vs. 14.4) in XIENCE V group as compared to TAXUS® group. In addition, for other key clinical endpoints the XIENCE V group also tend to have lower observed event rates than the TAXUS® group, e.g. ID-TLR (1.8% vs. 3.9%), ID-TVR (2.7% vs. 5.2%), ID-MACE (2.7% vs. 6.5%). There was 0.5% Late Stent Thrombosis (1/223) in the XIENCE V group versus 1.3% (1/77) in the TAXUS® group. There were no additional MACE events or Stent Thrombosis between 180 days and 270 days follow-up. Therefore, the angiographic, IVUS results at 6 months, and clinical endpoint results through 9 months have demonstrated the safety and efficacy of the XIENCE V EECSS.

SPIRIT III, a pivotal clinical trial conducted in the USA and Japan, was designed to compare XIENCE V EECSS performance to the TAXUS PECSS. The SPIRIT III USA randomized clinical trial indicates that XIENCE V EECSS efficacy is statistically non-inferior to the TAXUS PECSS in single and multiple de novo native coronary artery lesions.[18] Moreover, the primary endpoint was met with an in-segment late loss of 0.14 ± 0.41 mm for the XIENCE V EECSS arm and 0.28 ± 0.48 mm for the TAXUS PECSS arm at 240 days (non-inferiority $p < 0.0001$, delta = 0.195 mm).

The XIENCE V EECSS has received CE mark approval on 30 January 2006 and FDA approval 02 July 2008. In light of that, the SPIRIT V study has completed enrollment in approximately 100 sites outside the United States, and enrolled 3025 patients. It consists of two concurrent studies: a prospective, randomized, active-controlled, single blind, parallel two-arm multicenter study comparing XIENCE V to the Taxus Liberté™ in the treatment of diabetic patients with coronary artery lesions and a prospective, single arm, multi-center registry evaluating performance of XIENCE V in real-world use. In the registry, 30 day rates of in-hospital non-Q wave MI (1.9%), acute stent thrombosis (0.15%) and sub-acute stent thrombosis (0.3%) supported the acute safety of the XIENCE V EECSS. At 1 year the composite of Cardiac Death, MI attributed to the target vessel and TLR was 5.1% and the late definite and probable stent thrombosis was 0.23% with a cumulative rate of definite and probable stent thrombosis of 0.66% thus demonstrating the continued safety and efficacy of the XIENCE V EECSS.

In the Reality Study[xix], a large multi-centre study with a 'real world' patient population, the CYPHER™ arm MACE rate was reported at 240 days as 9.2% vs. 10.6% (p=ns) in the TAXUS® arm, with an in-stent late loss of 0.09 mm vs. 0.31 mm (p<0.0001) and an in-stent restenosis rate of 7.0% vs. 8.3% (p=ns) respectively.

In another trial with similar randomization arms, but from a single centre; the Sirtax Trial[xx], the reported MACE rates were 6.2% for the CYPHER™ arm at 270 days vs. 10.8% for the TAXUS® arm (p 0.009) with in-stent late loss of 0.13 mm vs. 0.25 mm (p<0.001) and in-stent restenosis rates of 3.2% vs. 7.5% respectively (p 0.013) [xx].

A third study including only diabetic patients; the ISAR-Diabetes Trial[xxi] had a smaller sample size population (125 in each arm) and reported no differences in MACE rates at 9 months but an in-stent late loss of 0.19 mm in the CYPHER™ arm vs. 0.46 mm (p<0.001) in the TAXUS® arm and restenosis rates of 6.9% vs. 16.5% respectively (p=0.03) [xxi]. This data is corroborated by IVUS data[xxii] and suggest that CYPHER™ may perform better than TAXUS®. in terms of late loss. However there has been no randomized control trial with a clinical primary endpoint that has shown superiority of one DES over the other[xxiii]. As SPIRIT II has demonstrated that XIENCE V is superior to TAXUS in terms of late loss, this study will compare XIENCE V to CYPHER™.

In light of the recommendations from the Policy Conference on Cardiovascular Diseases in Women of the European Society of Cardiology, SPIRIT WOMEN is designed to focus on specific aspects of women's health in relation to coronary artery disease, such as menopausal status, use of hormonal contraceptives or their surrogates, the referral path and symptoms at presentation, to better characterize the female population undergoing stent implantation and to continue the evaluation of safety and performance of XIENCE EECSS in real-world use in this specific population.

2.2 Description of XIENCE V EECSS

One of the test devices used in the SPIRIT WOMEN Clinical Evaluation is XIENCE V Everolimus Eluting Coronary Stent System (XIENCE V EECSS). The XIENCE V EECSS has received CE mark approval on 30 January 2006 and FDA approval 02 July 2008. In this Clinical Investigation Plan (CIP), the following abbreviations are used with regards to the XIENCE V.

Table 2.2: Abbreviations of XIENCE V Everolimus Eluting Coronary Stent System

XIENCE V EECSS	System (stent and delivery system)
XIENCE V EECS	Stent
XIENCE V	Product in General

The XIENCE V EECSS is manufactured by Abbott Cardiovascular Systems, Inc., California, an affiliate of Abbott Vascular Inc. The XIENCE V EECSS is comprised of the ML VISION® Stent (2.75, 3.0 mm, 3.5, 4.0 mm) or the ML MINI VISION® Stent (2.25, 2.5 mm) (EC 71619) on a delivery system (EC 71619) and a drug eluting coating.

The XIENCE V EECSS is a balloon expandable stent, fabricated from a single piece of medical grade L-605 Cobalt Chromium (CoCr) alloy and is similar in strut design to the ACS ML VISION® Stent and ML MINI VISION® Stent. The ML VISION® and the ML MINI VISION® stents are based on the same design principle as the ACS ML TETRA™ Stent (EC 54315). The ML VISION® and the ML MINI VISION® stents are balloon expandable stents that consist of serpentine rings connected by links.

Figure 2.2.1 Xience V Stent

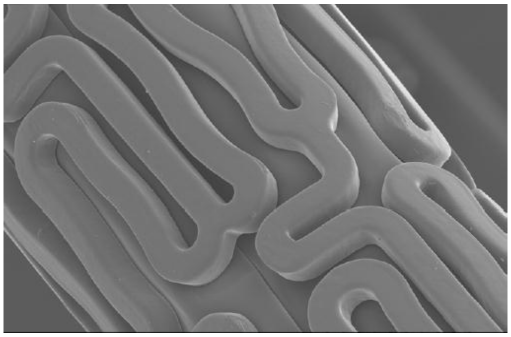

To maintain stent radiopacity with a thinner strut thickness, the ML VISION® and the ML MINI VISION® stents are made of L-605 CoCr alloy instead of the 316L Stainless Steel material used for the ACS ML TETRA™ Stent. The doses of everolimus on the XIENCE V EECSS are presented in the IFU of XIENCE V.

2.2.1 The XIENCE V EECSS Delivery System

The XIENCE V EECSS Delivery System is similar to the ML VISION® RX CSS and the ACS ML RX ZETA™ CSS (EC 60607) in delivery system design, performance specifications and material.

Like other ACS RX Coronary Stent Systems and Coronary Dilatation Catheters, the XIENCE V EECSS combines a single lumen proximal shaft with a dual lumen mid-shaft and a co-axial lumen distal shaft to create the rapid exchange capability. The single lumen proximal shaft connects the intermediate/distal shaft with the inflation port of the catheter. The guide wire exit notch is located at the proximal end of the junction between the intermediate shaft and the mid-shaft support. The overall length of the catheter is 143 cm.

Two radiopaque balloon markers are located on the distal segment of the inner member and are positioned to mark the working length of the balloon. The stent is mounted such that the markers reflect the expanded stent length. The radiopaque markers aid in positioning the stent fluoroscopically and also aid in accurately positioning the delivery system for post-deployment dilation, if necessary.

There are two non-radiopaque markers on the proximal shaft of the XIENCE V EECSS. These two markers, located 95 cm and 105 cm proximal to the distal tip, indicate when the distal tip of the catheter

exits the tip of a brachial or femoral guiding catheter, respectively. These markers can be visually tracked on the shaft as the catheter enters the sheath.

A single arm adapter is attached to the proximal end of the catheter and accesses the inflation/deflation lumen. The proximal shaft is thermally bonded to a nylon adaption cup, and the adaption cup is mechanically sealed to the single arm adapter with a polycarbonate nosepiece, which is threaded onto the single arm adapter and bonded together with methylene chloride.

A 0.014-inch or smaller diameter guide wire can be used in the guide wire lumen. The guide wire exits the guide wire lumen at the guide wire exit notch, which is formed at the junction of the mid-shaft and the intermediate shafts. Proximal to this point, the guide wire runs externally alongside the proximal shaft of the catheter.

2.2.2 The XIENCE V EECSS Polymer Coating

The XIENCE V EECSS has a drug eluting coating consisting of two polymers and the drug everolimus. The coating is composed of acrylic and fluoro polymers, both previously approved for use in blood contacting applications. These materials are components of vascular sutures and other drug eluting coronary stents.

2.2.3 The Anti-proliferative Drug: Everolimus

Everolimus [40-O-(2-hydroxyethyl)-rapamycin], which is provided to Abbott Cardiovascular Systems, Inc. by Novartis Pharmaceuticals Corporation, is a semisynthetic macrolide immunosuppressant, from the same family as Rapamycin (INN: sirolimus). Everolimus is approved in the EU and marketed as Certican.

Further information regarding everolimus pre-clinical and clinical data can be found in the investigator's brochure.

2.3 Description of XIENCE PRIME EECSS

The second test device used in the SPIRIT WOMEN Clinical Evaluation (RSS only) is XIENCE PRIME Everolimus Eluting Coronary Stent System (XIENCE PRIME EECSS). The XIENCE PRIME EECSS has received CE mark approval on 23 June 2009. The XIENCE PRIME EECSS is manufactured by Abbott Cardiovascular Systems, Inc., California, an affiliate of Abbott Vascular Inc.

The XIENCE PRIME stent system is similar to that of the Abbott Vascular XIENCE V Everolimus Eluting Coronary Stent System (EECSS) (CE Mark, January 2006). The XIENCE PRIME stent uses the identical stent, balloon material, and drug coating formulation as the XIENCE V stent. However, the XIENCE PRIME stent design has been slightly modified from that of the XIENCE V stent in order to accommodate design improvements while not affecting the overall structural integrity of the design. These modifications include longer cell length for optimized stent retention, taller non-linear links for improved flexibility, straighter bar arms to reduce strut interference and to better maintain coating integrity during the crimping process, and a modified proximal end ring to reduce strut lifting. The delivery system utilizes the same principle of operation as other Abbott Vascular Rapid Exchange (RX) coronary stent systems and coronary dilatation catheters.

There are two stent designs for the XIENCE PRIME stent. The small XIENCE PRIME stent design is intended for the 2.25, 2.5 and 3.0 mm expansion diameters while the medium XIENCE PRIME stent design is intended for the 3.5 and 4.0 mm expansion diameters.

2.4 Description of the CYPHER SELECT™ Plus

The CYPHER SELECT™ Plus Sirolimus-eluting Coronary Stent (Cordis Corporation) is to be used as an active-control device in the randomized sub-study. CYPHER uses Sirolimus as an anti-proliferative drug and obtained FDA approval in 2003 and is commercially available throughout Europe and international markets including the United States. In the event a subsequent generation of CYPHER SELECT™ Plus Sirolimus-eluting Coronary Stent is released during the course of the study, it may replace the CYPHER SELECT™ Plus Sirolimus-eluting Coronary Stent.

3 CLINICAL EVALUATION OBJECTIVE

The objective of this clinical evaluation is a continuation in the assessment of the performance of the XIENCE EECSS with the primary focus on clinical outcomes in the treatment of female patients with de novo coronary artery lesions. The study will also provide characterization of the female population undergoing stent implantation with the XIENCE EECSS.

4 CLINICAL EVALUATION DESIGN

The SPIRIT WOMEN Clinical Evaluation is a prospective, open label, single arm, multi-center study evaluating performance of the XIENCE V EECSS in the treatment of female patients with coronary artery lesions, per its Instructions for Use (IFU). Additionally, this study will include a prospective, single-blind, double arm, randomized multi-center sub-study comparing the XIENCE EECSS (XIENCE V EECSS and XIENCE PRIME EECSS) to the CYPHER SELECT™ Plus Sirolimus-eluting Coronary Stent in the treatment of female patients with coronary artery lesions.

- Approximately 1550 patients will be enrolled in the single arm study (SPIRIT WOMEN Single Arm Study).
- Approximately 450 patients will be randomized 2:1 (XIENCE EECSS vs. CYPHER SELECT™ Plus Sirolimus-eluting Coronary Stent) from up to 35 selected sites (randomized sub-study).

The test device used is XIENCE Everolimus Eluting Coronary Stent System (XIENCE V EECSS and XIENCE PRIME EECSS (randomized sub-study only)). The XIENCE V EECSS has received CE mark approval on 30 January 2006 and FDA approval 02 July 2008. The XIENCE PRIME EECSS has received CE mark approval on 23 June 2009.

The control device used in the randomized sub-study is CYPHER SELECT™ Plus Sirolimus-eluting Coronary Stent. In case a subsequent generation of CYPHER SELECT™ Plus Sirolimus-eluting Coronary Stent is released during the course of the study, it may replace the CYPHER SELECT™ Plus Sirolimus-eluting Coronary Stent.

Assessment of anginal status, collection of data regarding adverse events, details of any subsequent coronary interventions, Clinical Investigation Plan (CIP) medications use and changes in concomitant medications will be collected at 30 days, 240 days and 1, 2, 3, 4 and 5 years post-procedure for all patients.

Evaluation of specific female demographics such as menopausal, hysterectomy and oophorectomy status, past and current use of hormonal contraceptives, and use of weak estrogens will occur for all patients.

4.1 Patient Follow-up

All patients will have clinical follow-up at 30 days, 240 days and at 1, 2, 3, 4 and 5 years.

A subgroup of approximately 450 patients will have angiographic follow-up at 270 days (patients in the randomized sub-study).

4.2 Treatment Strategy

The treatment strategy for all patients enrolled in the clinical evaluation is as follows:

The treatment strategy will be determined by the Investigator. It is recommended that each enrolling investigator review the most recent IFU to assess contraindications, warnings, and precautions for treating potential patients.

Loading dose of clopidogrel of at least 300 mg prior to the start of the procedure. This dose may be adjusted if the patient is already taking clopidogrel. Recommended prior to the start of the procedure, however no later than 1 hour post procedure.

No non-study percutaneous procedures can be performed at the time of the study procedure

Additional treatment strategy for patients enrolled in the Single Arm Study is as follows:
After meeting the general and angiographic inclusion and exclusion criteria, the target lesions should be treated by stenting with the XIENCE V EECSS according to the IFU.

Registration should only be done after confirmation of the angiographic inclusion criteria and before implantation of the first stent.

All lesions should be planned to be treated with the XIENCE V.

If any staged procedures are planned at baseline, it is necessary that the investigator treats the enrolled patients with only XIENCE V EECSS during the planned staged procedure.

Bailout stenting should be performed with XIENCE V EECSS of appropriate length.

It is recommended that all patients should receive ≥ 75 mg of aspirin daily for a minimum of five years following the procedure.

It is recommended that treatment with clopidogrel should be continued at 75 mg daily for a period of at least 6 months. Ticlopidine at a dose standard with hospital practice may be substituted for clopidogrel, as required by hospital practice or if the patient develops hypersensitivity to clopidogrel.

Additional treatment strategy for patients enrolled in the randomized sub-study is as follows:
The test devices used are the XIENCE V Everolimus Eluting Coronary Stent System (XIENCE V EECSS and the XIENCE PRIME Everolimus Eluting Coronary Stent System (XIENCE PRIME EECSS.

After meeting the general and angiographic inclusion and exclusion criteria, the target lesion(s) should be treated according to the same randomization schedule. If two or more target lesions are to be treated, all lesions must receive the treatment that has been assigned as per the randomization.

If two or more target lesions are to be treated and the patient is randomized to the XIENCE EECSS, all lesions must receive either the XIENCE V EECSS or the XIENCE PRIME EECSS.

Randomization should be done only after confirmation of the angiographic inclusion criteria and before implantation of the first stent. Once randomization is completed and a treatment is assigned, crossover is not permitted.

If it is not possible to implant the stent allocated by randomization, it is recommended that any other commercially available stent, either BMS or DES be implanted, rather than the XIENCE or the CYPHER SELECT™ Plus Sirolimus-eluting Coronary Stent.

If bailout stenting is necessary, the stent used should be the same as allocated by randomization for the index procedure, and should be used according to the Instructions for Use (IFU).

If any staged procedures are planned at baseline, it is necessary that the investigator treats the enrolled patients with the same study stent during the planned staged procedure as was used at baseline (as allocated at randomization).

All patients should receive ≥ 75 mg of aspirin daily for a minimum of five years following the procedure.

All patients should receive clopidogrel 75 mg daily for a period of at least 6 months Ticlopidine at a dose standard with hospital practice may be substituted for clopidogrel, as required by hospital practice or if the patient develops hypersensitivity to clopidogrel.

Treatment strategy for the CYPHER SELECT™ Plus Sirolimus-eluting Coronary Stent

Use of the CYPHER SELECT™ Plus Sirolimus-eluting Coronary Stent should be according to its Instructions for Use.

4.3 Measures to be taken to Avoid/Minimize Bias

A centralized randomization/registration service will be used. Randomization/registration should be done only after confirmation of angiographic inclusion criteria and before implantation of the first stent. Once randomized/registered the patient is considered enrolled in the study.

A subset of approximately 450 patients will be randomized in a 2:1 ratio in the randomized sub-study. Randomization will be stratified by diabetes mellitus (diabetic vs. non-diabetic), and lesion characteristics (complex vs. non-complex). Complex lesion characteristics include triple vessel treatment, or dual lesion per vessel treatment, or lesions involving RCA-aorto-ostial locations, or bifurcations lesions in which the side branch is ≥ 2 mm in diameter, or bifurcation lesions in which the ostium of the side branch is > 50% stenosed. For patients enrolled in the randomized sub-study, once randomization is completed and a treatment is assigned, crossover is not permitted.

5 ENDPOINTS

5.1 Primary Endpoint

The following primary endpoint will be examined:

Adjudicated Composite rate of all Death, all Myocardial Infarction (MI) and Target Vessel Revascularization (TVR) at 1 year.

5.2 Secondary Endpoints

The following secondary endpoints will be examined:

Acute Success (Clinical Device Success and Clinical Procedure Success)

Adjudicated Stent Thrombosis at 30 days, 240 days and at 1, 2, 3, 4 and 5 years (Definite, Probable, Possible)

Adjudicated revascularization (TLR/TVR/all revascularizations) at 30 days, 240 days and at 1, 2, 3, 4 and 5 years

Adjudicated Composite rate of Cardiac Death, MI attributed to the target vessel and CI-TLR at 30 days, 240 days and at 1, 2, 3, 4 and 5 years.

Adjudicated Composite rate of all Death, all MI and Target Vessel Revascularization (TVR) at 30 days, 240 days and at 2, 3, 4 and 5 years.

Adjudicated Composite rate of all Death, all MI and any Revascularization (TLR/TVR/non TVR) at 30 days, 240 days and at 1, 2, 3, 4 and 5 years.

Adjudicated Cardiac Death, Non-Cardiovascular Death, Vascular Death, Q-wave MI and Non Q-wave MI (Peri-Procedural, Unrelated to PCI) at 30 days, 240 days and at 1, 2, 3, 4 and 5 years.

In-stent Late Loss (LL) at 270 days (*main* secondary endpoint for the randomized sub-study)

In-segment Late Loss (LL) at 270 days (for the randomized sub-study)

In-stent and in-segment Angiographic Binary Restenosis rates at 270 days (randomized sub-study)

In-stent and in-segment percent Diameter Stenosis at 270 days (randomized sub-study)

Aneurysm, thrombus and persisting dissection at 270 days (for the randomized sub-study)

Additional analysis of specific female demographics such as menopausal, hysterectomy and oophorectomy status, past and current use of hormonal contraceptives, and use of weak estrogens will be outlined in the Statistical Analysis Plan (SAP).

6 SELECTION AND WITHDRAWAL OF PATIENTS

6.1 Patient Population

Patients enrolled into this Clinical Evaluation will be comprised of female patients derived from the general interventional cardiology population. The Clinical Evaluation will enroll a total of approximately 2000 'real world' patients who are screened in accordance with the commercial IFU of the XIENCE EECSS and who meet all eligibility criteria and have provided written informed consent.

6.2 Patient Screening

Patients planned to be admitted for a percutaneous coronary artery revascularization procedure should be screened for Clinical Evaluation eligibility by a member of the research team previously trained to the clinical investigation plan. Patients who do not satisfy inclusion and exclusion criteria will not be enrolled in this clinical evaluation. After meeting the general inclusion and exclusion criteria, patients will be asked to sign an informed consent. All patients who have signed an informed consent will be entered into the Screening log in InForm**.** (Refer to Appendix VI, Screening Process)

6.2.1 Informed Consent

Informed Consent needs to be signed and dated by the investigator and the patient or by her legal representative before any study-related procedure and before the pre-procedure angiographic screening procedure. The investigator or a person designated by the investigator who has been trained on the clinical investigation plan, will explain the nature and scope of the clinical evaluation, potential risks and benefits of participation, and answer the patient's questions. If the patient agrees to participate, the informed consent form must be completed, signed and personally dated by the patient or legally authorized representative. A copy of the completed informed consent form must be provided to the patient. The obtaining of the consent, provision of a copy to the subject, along with the date and time should be documented in the subject's medical records and signed by the investigator.

All patients must provide informed consent in accordance with the local Medical Ethics Committee (MEC) requirements, using an MEC-approved informed consent form. Appendix VI outlines the screening process and illustrates the point where informed consent should be obtained.

6.2.2 Ethical Considerations

This study will be conducted in accordance with the Declaration of Helsinki, BS EN ISO 14155-1:2003 and BS EN ISO 14155-2:2003 standards provided these do not oppose the applicable law. The conduct of the study will be approved by the appropriate MEC of the respective clinical site and as specified by local regulations.

6.3 Allocation of Patients to the SPIRIT WOMEN Single Arm Study and Randomized Sub-study

Up to 35 selected sites will participate in both the randomized sub-study and the Single Arm Study. In order to avoid bias in the population of the SPIRIT WOMEN Clinical Evaluation, these 35 sites will only recruit patients into the randomized sub-study, and not into the SPIRIT WOMEN Single Arm Study, during the recruitment period of the randomized sub-study. These sites can enroll all eligible patients into the SPIRIT WOMEN Single Arm Study outside of recruitment period of the randomized sub-study.

Figure 6.3: Allocation of Patients

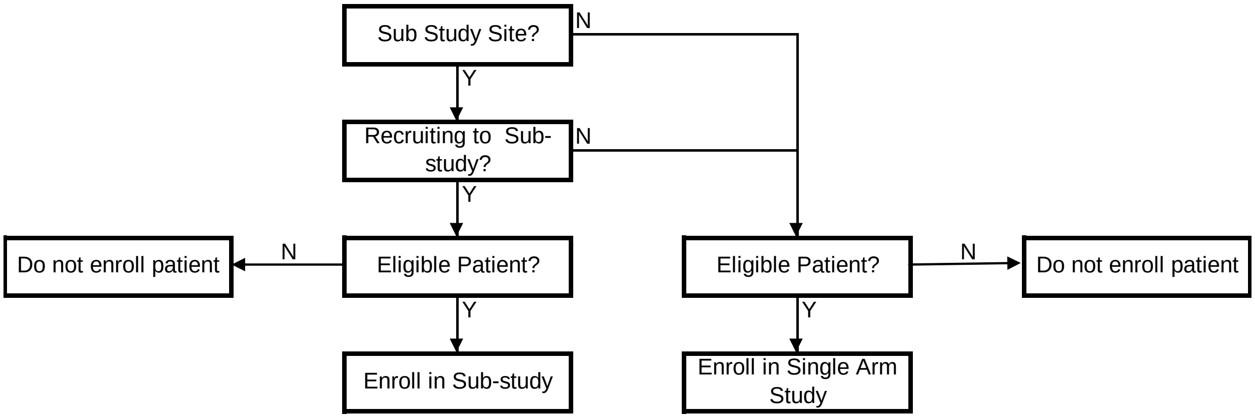

6.4 Eligibility Criteria

Patients must meet **ALL** of the inclusion criteria to be considered for the clinical evaluation. If **ANY** of the exclusion criteria are met, the patient is excluded from the clinical evaluation and cannot be enrolled.

6.4.1 General Inclusion Criteria

- Patient must be female.
- Patient must be at least 18 years of age.
- Patient is able to verbally confirm understanding of risks, benefits and treatment alternatives and she or her legally authorized representative provides written informed consent prior to any study related procedure, as approved by the appropriate Medical Ethics Committee of the respective clinical site.
- Patient must have evidence of myocardial ischemia (e.g., stable or unstable angina, silent ischemia, positive functional study or a reversible change in the electrocardiogram (ECG) consistent with ischemia).
- Patient must be an acceptable candidate for coronary artery bypass graft (CABG) surgery.
- Patient must agree to undergo all CIP-required follow-up examinations.
- Patients of childbearing potential must have had a negative pregnancy test within 7 days before treatment, and must not be nursing at the time of treatment.

6.4.2 Angiographic Inclusion Criteria

- Patients' artery morphology and disease is suitable to be optimally treated with a maximum of 4 planned study stents.

- Target lesions must be de novo lesions (no prior stent implant, no prior brachytherapy).
- Target vessel reference diameter must be between 2.5 mm and 4.0 mm by visual estimate. The diameter range will be expanded to 2.25 mm when the 2.25 mm stent is available.
- Target lesion ≤ 28 mm in length by visual estimate.

6.4.3 General Exclusion Criteria

- Patient has other medical illness (e.g., cancer or congestive heart failure) or known history of substance abuse (alcohol, cocaine, heroin etc.) that may cause non-compliance with the clinical investigation plan, confound the data interpretation or is associated with a limited life expectancy (i.e., less than one year).
- Patient has a known hypersensitivity or contraindication to aspirin, either heparin or bivalirudin, both clopidogrel and ticlopidine, sirolimus, everolimus, cobalt, chromium, nickel, tungsten, acrylic and fluoro polymers or contrast sensitivity that cannot be adequately pre-medicated.
- Participation in another device or drug study or has completed the follow-up phase of another study within the last 30 days.
- Patient who is judged to have a lesion that prevents complete inflation of an angioplasty balloon.
- Patient has had a previous stent implant, either Bare Metal Stent (BMS) or Drug Eluting Stent (DES) within the target vessel(s)

6.5 Patient Discontinuation

Patients are considered enrolled in the study from the time of Registration or Randomization. Every patient should remain in the clinical evaluation until completion of the required follow-up period, however, a patient's participation in any clinical evaluation is voluntary and the patient has the right to withdraw at any time without penalty or loss of benefit. Should this occur, the reason for withdrawal must be documented in the source documentation. Conceivable reasons for discontinuation may include, but not be limited to, the following:

Patient Withdrawal: Patient participation in a clinical investigation is voluntary and the patient may discontinue participation (refuse all subsequent testing/follow-up) at any time without loss of benefits or penalty.

Investigator Termination: Investigator may terminate the patient's participation without regard to the patient's consent if the Investigator believes it is medically necessary.

Lost-to-Follow-up: Patient does not complete the scheduled follow-up visits but has not 'officially' withdrawn from the clinical evaluation. (Does not apply to missed visits). Site personnel should make all reasonable efforts to locate and communicate with the patient, including the following, at each contact time point:

- A minimum of two telephone calls to contact the patient should be recorded in the source documentation, including date, time, and initials of site personnel trying to make contact.
- If these attempts are unsuccessful, a certified letter should be sent to the patient and/or the patient's General Practitioner/Family Doctor. (this should be done only when the patient has consented to direct contact with General Practitioner/Family Doctor)
- If a patient misses one or more of the follow-up contact time points it will be considered a missed visit. Patient may then return for subsequent visits. If the patient misses two

consecutive time points and the above-mentioned attempts at communicating with the patient are unsuccessful, the patient will be considered lost-to-follow-up.

A Study Completion form must be completed when,

- the patient is considered lost to follow-up per the above definition or
- the patient withdraws from the Clinical Evaluation or
- the investigator withdraws the patient from the Clinical Evaluation or
- the patient has died or
- upon Clinical Evaluation completion (5 year follow-up time point has been reached)

Sponsor must be notified of the reason for patient discontinuation. The site will provide this information on the eCRF. Investigators must also report this to their MEC as defined by their institution's procedure. Patients will not be replaced.

7 TREATMENT OF PATIENTS

The schedule of events for this clinical evaluation is located in Appendix V.

7.1 Pre-Procedure (Baseline)

7.1.1 Patient History

Patient history will include demographics (e.g., age), symptoms at presentation and referral path, cardiac history including but not limited to Canadian Cardiovascular Society (CCS) and Braunwald classification of angina, history of myocardial infarction, diabetes mellitus, renal insufficiency, left ventricular dysfunction, hypertension, hypercholesterolemia, previous CABG and PCI. Data recorded will also include menopausal, status/years since onset of menopause, hysterectomy status, oophorectomy status, use of weak estrogens (black cohosh, soy, red clover), past and present use of hormonal contraceptives/hormone replacement therapy (including mode of administration), smoking history, educational/professional/social status such as number of children, cohabitation, employment and educational degree, body surface area and chronic concomitant medications (Refer to Appendix II for Definition).

7.1.2 Laboratory Assessments

Routine hospital blood-work should be taken for all patients per hospital standards for patient management. Additional samples for local (all patients) and central laboratory measurements (for patients enrolled in the randomized sub-study) must be taken as described below.

The following laboratory assessments must be obtained within 7 days prior to the index procedure:

Local laboratory for all patients	Central laboratory for patients enrolled in randomized sub-study
Creatine kinase myocardial-band isoenzyme (CK-MB) or Troponin I/T	Creatine kinase myocardial-band isoenzyme (CK-MB)
Pregnancy test (if applicable)	Total Cholesterol, HDL, LDL, triglycerides
	High-sensitivity C-reactive protein (hs-CRP)
	Glycosylated hemoglobin (HbA_{1C})
	17ß-estradiol
	Estrone
	FSH level
	Free Testosterone
	Dehydroepiandrosterone (DHEA)
	Androstenedione
	Sex Hormone Binding Globulin (SHBG)
	Homocysteine
	Creatinine

7.1.3 Other Assessments

The following parameters must be measured within 7 days prior to the index procedure:

- ECG
- Height (cm)
- Weight (kg)
- Waist circumference at umbilical level (cm)
- Blood pressure

7.1.4 Medications

Patients selected for coronary stenting should receive a loading dose of antiplatelet medications (unless patient is under chronic antiplatelet medications) prior to the start of the procedure, but no later than one hour post procedure. Medications administered will include loading dose of clopidogrel bisulfate (≥ 300 mg) and aspirin (≥ 75 mg). Ticlopidine at a dose standard with hospital practice may be substituted for clopidogrel, as required by hospital practice or if the patient develops hypersensitivity to clopidogrel. If patient is already receiving clopidogrel bisulfate (or ticlopidine hydrochloride), loading dose can be adjusted per physician's discretion, but not omitted.

7.2 Enrollment

After meeting the general inclusion and exclusion criteria, written informed consent must be obtained before the pre-procedure angiography. The angiograms acquired just before the intended procedure (i.e. the pre-procedure angiography) will be used to assure by visual assessment that the reference diameter(s) is ≥ 2.5* mm and ≤ 4.0 mm and the lesion length ≤28mm.

*expanded to 2.25 in diameter when 2.25 mm stent available

Once the angiographic inclusion criteria have been confirmed, the patient should be registered / randomized via the IVRS system.

Due to phased start-up of the clinical sites and to allow all sites to have the opportunity to enroll patients in the study, sites may be asked to delay further recruitment once they have enrolled 50 patients in the SPIRIT Women Single Arm Study and 45 patients in the randomized sub-study.

7.3 Procedure

7.3.1 Medications

During the procedure, patients will receive appropriate anticoagulation and other therapy according to standard hospital practice. Either unfractionated heparin or bivalirudin may be used for procedural anticoagulation. The use of Glycoprotein IIb/IIIa inhibitors is left to the discretion of the investigator.

7.3.2 Baseline Angiography

The angiogram acquired during the index procedure (i.e. the baseline angiography) will be assessed visually and parameters will be documented in the eCRF.

7.3.3 Stenting Procedure

7.3.3.1 SPIRIT WOMEN Single Arm Study

Once the general and angiographic inclusion and exclusion criteria have been confirmed, the patient should be registered via the IVRS registration system before the placement of the first stent.

Treatment of target lesion(s) should be with either a single stent or planned overlapping stents.

Prior to use, the XIENCE V EECSS should be inspected and prepared according to the Instructions for Use (IFU). All XIENCE V EECSS should be delivered and deployed per the IFU.

All the target lesion(s) should be treated by stenting with the XIENCE V EECSS.

7.3.3.2 Randomized Sub-study

Baseline angiography of the target vessel will be completed as per the Angiographic Core Laboratory Guidelines.

Once the general and angiographic inclusion and exclusion criteria have been confirmed, the patient should be randomized via IVRS randomization service prior to the placement of the first stent.

Prior to use, the XIENCE EECSS should be inspected and prepared according to the Instructions for Use (IFU). All XIENCE EECSS should be delivered and deployed per the IFU.

All CYPHER SELECT™ Plus Sirolimus-eluting Coronary Stent use should be according to its IFU.

All target lesion(s) should be treated by stenting with the stent assigned per randomization, either XIENCE EECSS or CYPHER SELECT™ Plus Sirolimus-eluting Coronary Stent.

Treatment of target lesion(s) should be with either a single stent or planned overlapping stents.

7.3.4 Treatment Rules for the XIENCE EECS

The treatment strategy will be determined by the investigator. It is recommended that each enrolling investigator review the most recent XIENCE IFU to assess contraindications, warnings, and precautions for treating potential patients.

7.3.5 Angiography Following Stent Placement

In all patients, an angiographic imaging will be performed immediately following stent placement. Post-stenting images should be captured in the same manner as was used for the pre-procedure images.

For patients enrolled in the randomized sub-study, the procedure should be performed according to the angiographic core laboratory guidelines.

7.3.6 Bailout Procedures

Bailout procedures may be performed if the patient experiences any of the following during the index procedure:

Major dissection (type C or greater)

Occlusive complication as evidenced by a decrease in target vessel flow

Chest pain or ischemic ECG changes that do not respond to repeat balloon inflations, medical therapy or lytic agents

Unplanned additional stent is required to cover the target lesion

SPIRIT WOMEN Single Arm Study: If bailout stenting is necessary, a XIENCE V EECSS of appropriate length should be used.

Randomized sub-study: If bailout stenting is necessary, the stent used should be the same as allocated by randomization for the index procedure and should be used according to the Instructions for Use (IFU).

IMPORTANT: For the XIENCE EECSS it is recommended that bailout stent should be placed so that there is no visible gap between the stents. In such a case, at least 1 mm (minimum) to 4 mm (maximum) overlap is recommended.

A bailout procedure is not considered an endpoint event unless the patient sustains death, emergent CABG or MI. Bailout procedures should be avoided unless required for safe patient management. If the bailout procedure was a result of an adverse event/serious adverse event, an appropriate electronic Case Report Form (eCRF) must be completed for this event.

7.4 Post-Procedural Testing and Medications

Following the procedure, all patients will be treated in accordance with the hospital standard of care.

7.4.1 ECG

.An ECG must be taken within the post-procedure hospitalization period for all patients.

7.4.2 Cardiac Enzymes and Creatinine

Routine hospital blood-work and testing should be taken / performed per hospital standards for patient management and must include at least the following:

7.4.2.1 XIENCE V SPIRIT WOMEN Single Arm Study (Local Laboratory)

One sample for CKMB or Troponin I/T must be taken in the post-procedural hospitalization period, but no later than 24 hours after the procedure.

7.4.2.2 Randomized Sub-study (Central Laboratory)

One sample for CKMB or Troponin I/T must be taken in the post-procedural hospitalization period. A second sample should be taken within 3 to 6 hours after the first post-procedure sample, but no later than 24 hours after the procedure.

One sample for Creatinine must be taken in the post-procedural hospitalization period.

7.4.3 Medications

7.4.3.1 SPIRIT WOMEN Single Arm Study

It is recommended that patients enrolled in the SPIRIT WOMEN Single Arm Study should be maintained on 75 mg of clopidogrel bisulfate daily for a minimum of 6 months following the procedure. Ticlopidine at a dose standard with hospital practice may be substituted for clopidogrel, as required by hospital practice or if the patient develops hypersensitivity to clopidogrel.

It is also recommended that all patients should receive ≥ 75 mg of aspirin daily for a minimum of 5 years following the index procedure.

Start of these treatments will be documented in the eCRF as well as any changes to and termination of treatment.

7.4.3.2 Randomized Sub-study

Patients enrolled in the randomized sub-study will be maintained on 75 mg of clopidogrel bisulfate daily for a minimum of 6 months following the procedure. Ticlopidine at a dose standard with hospital practice may be substituted for clopidogrel, as required by hospital practice or if the patient develops hypersensitivity to clopidogrel.

All patients will receive ≥ 75 mg of aspirin daily for a minimum of five years following the procedure.

Start of these treatments will be documented in the eCRF as well as any changes to and termination of treatment.

8 ASSESSMENT OF SAFETY and PERFORMANCE

Safety and performance will be evaluated at several time points throughout the clinical evaluation. See Appendix IV, Clinical Investigation Flow and Appendix V, Schedule of Events.

8.1 Clinical Follow-up for All Patients

Clinical follow-up will be performed periodically at the following intervals for all patients:

30 days ± 7days follow-up telephone contact/office visit

240 days ± 14 days follow-up telephone contact/office visit

1 year ± 28 days follow-up telephone contact/office visit

2 year ± 28 days follow-up telephone contact/office visit

3 year ± 28 days follow-up telephone contact/office visit

4 year ± 28 days follow-up telephone contact/office visit

5 year ± 28 days follow-up telephone contact/office visit

The following information will be collected at each of the time points:

Assessment of anginal status

Data regarding adverse events*, including related laboratory tests, medications and/or ECGs

* Following the 2 year follow-up, it is mandatory only to report and collect information on serious adverse events, cardiac adverse events and device related events.

Details of any subsequent repeat coronary angiography and results of such, if applicable

Details of any subsequent coronary interventions (e.g., repeat PCI or CABG)**

Use and compliance of medications to clinical investigation plan (Aspirin and Clopidogrel)

Use and changes in chronic concomitant medications

**All TLR in the Single Arm Study will be clinically indicated.

8.1.1 Additional Follow-up Visits for All Patients

Additional patient visits may occur as clinically warranted. The following information will be collected:

Assessment of anginal status

Details of any subsequent coronary interventions (e.g., repeat PCI or CABG)*

Use and compliance to Clinical Investigational Plan medications (Aspirin and clopidogrel)

Use and changes in chronic concomitant medications (Refer to Appendix II for Definition).

*All TLR in the Single Arm Study will be clinically indicated.

All efforts must be made to obtain follow-up information on patients who have visited, undergone procedures or have been treated for adverse events in a non-study-related hospital(s).

8.2 *Angiographic Follow-Up for Randomized Sub-study*

In addition to the above follow-up visits, imaging (angiography) follow-up will be performed at the following interval on the approximately 450 patients enrolled in the randomized sub-study:

270 days (9 months) ± 14 days

Information will be collected in accordance with the Angiographic Core Laboratory requirements. Images for all target lesions identified at index procedure must be captured during the 9-month follow-up angiography.

* All revascularization in the Randomized Sub-study should be classified prospectively as clinically indicated or not clinically indicated by the investigator prior to the reintervention.

**Single target lesion at baseline**	**Is 9-Month angiographic follow-up required?**
Revascularization on target lesion before 270 days	No (Unless clinically indicated. Films of target lesion need to be made with the correct angulations and following core lab guidelines before the revascularization)
Angiography without target lesion revascularization post-index procedure and prior to 120 days	Yes
Angiography without target lesion revascularization post 120 days and prior to 270 days	No (Unless clinically indicated. Films of target lesion need to be made with the correct angulations and following core lab guidelines before the revascularization)
All patients are to continue their clinical follow-up visits as described per CIP	

**Multiple target lesions at baseline**	**Is 9-Month angiographic follow-up required?**
Revascularization on all target lesions before 270 days	No (Unless clinically indicated. Films of target lesion need to be made with the correct angulations and following core lab guidelines before the revascularization)
Revascularization on one target lesion prior to 120 days	Yes (angiography of all target lesions)
Angiography without target lesion revascularization post-index procedure and prior to 120 days	Yes (angiography of all target lesions)

Angiography with or without target lesion revascularization post 120 days and prior to 270 days (images for all target lesions captured)	No (Unless clinically indicated or some target lesions were not captured in previous angiography. Films of target lesion need to be made with the correct angulations and following core lab guidelines before the revascularization)
All patients are to continue their clinical follow-up visits as described per CIP	

Follow-up angiography must be performed only for patients who have received at least one study stent.

Collection of angiographic data must be according to the Angiographic Core Laboratory guidelines.

8.3 Adverse Events

In order to comply with applicable regulations and Vigilance requirements:

All adverse events and adverse device effects will, at a minimum, be classified in accordance with ISO 14155: 2003 Part 1 and Part 2 and reported according to the Sponsor's internal procedures to investigators, Ethics Committees and Regulatory Authorities.

8.3.1 Definitions

Adverse Event: Any untoward medical occurrence in a patient. This definition does not imply that there is a relationship between the adverse event and the device under investigation.

Adverse Device Effect: Any untoward and unintended response to a medical device. This definition includes any event resulting from insufficiencies or inadequacies in the instructions for use or the deployment of the device. It also includes any event that is a result of a user error.

Serious Adverse Event: Adverse event that:

- led to a death,
- led to a serious deterioration in the health of the patient that:
  - resulted in a life-threatening illness or injury,
  - resulted in a permanent impairment of a body structure or a body function,
  - required in-patient hospitalization or prolongation of existing hospitalization
  - resulted in medical or surgical intervention to prevent permanent impairment to body structure or a body function
- led to fetal distress, fetal death or a congenital abnormality or birth defect

Serious Adverse Device Effect: Adverse device effect that has resulted in any of the consequences characteristic of a serious adverse event or that might have led to any of these consequences if suitable action had not been taken or intervention had not been made or if circumstances had been less opportune.

8.3.2 Adverse Event Reporting

The Investigator will monitor the occurrence of adverse events for each patient during the course of the Clinical Evaluation. All adverse events (AEs) reported by the patient, observed by the Investigator, or documented in medical records will be listed on the adverse event case report forms, whether believed by the Investigator to be related or unrelated to the investigational device implant. Beginning with the start of the index procedure, which is defined as the time of the arterial puncture, any new event/experience that was not present at baseline, or worsening of an event present at baseline, is considered an adverse event and should be reported as such.

Note: Unchanged, chronic conditions are not adverse events and should not be recorded on the adverse event form of the eCRF.

An adverse event may be expected, (however should still be reported). Expected AEs may include:

Abrupt closure, Acute myocardial infarction, Allergic reaction to contrast, Aneurysm, Arterial perforation, Arterial rupture, Arteriovenous fistula, Arrhythmias (including atrial and ventricular), Bleeding complications (which may require transfusion), Coronary artery spasm, Coronary or stent embolism, Coronary or stent thrombosis, Death, Dissection of the coronary artery, Distal emboli (air, tissue or thrombotic), Drug reactions to anti-platelet agents/contrast medium, Emergent or non-emergent Coronary Artery Bypass Graft Surgery, Fever, Hypotension/Hypertension, Hypersensitivity reactions, Infection and pain at insertion site, Injury to the coronary artery, Ischemia, myocardial, Myelosuppression, Nausea and vomiting, Palpitations, Peripheral ischemia (due to vascular or nerve injury), Pseudoaneurysm, Restenosis of stented segment, Cerebrovascular accident (CVA), Total occlusion of coronary artery, Unstable or stable angina pectoris, Vascular complications (including entry site, which may require vessel repair), Ventricular arrhythmias (including ventricular fibrillation and ventricular tachycardia), Vessel dissection.

Adverse events will be monitored until they are adequately resolved or explained and reported to the DSMB on an ongoing basis throughout the clinical evaluation.

Determination of whether there is a reasonable possibility that an investigational product or device caused or contributed to an AE is to be determined by the Investigator and recorded on the CRFs as **not related, related or unknown.** Determination should be based on assessment of temporal relationships, biologic plausibility, association (or lack of association) with underlying disease, and presence (or absence) of a more-likely cause.

Definitions for determination of the relationship include:

Not Related: Exposure to the device has not occurred, or the occurrence of the adverse event is not reasonably related in time, or the adverse event is considered unlikely to be related to the use of the device (biologically implausible).

Related: Exposure to the device and the adverse event are reasonably related in time and the device is more likely than other causes to be responsible for the adverse event, or is the most likely cause of the adverse event.

Unknown: Exposure to the device and the occurrence of adverse event cannot be reasonably determined to be unrelated to the device. If the relationship is identified as unknown it will be treated as related to the device.

If any adverse event is considered related to the administration of the device, that event will be followed until resolution or the Investigator judges the event to be chronic or stable.

8.4 Serious Adverse Events

Each adverse event or complication meeting the definition for serious adverse event **whether or not considered device-related** should be considered as such and reported to Sponsor **within 24 hours of knowledge of event by entering the event into the electronic database**. The completion of an Adverse Event / Serious Adverse Event (AE/SAE) form in the eCRF will trigger an e-mail notification to Sponsor containing the event information. The investigator will report the event to the EC according to the institution's reporting requirements.

Reports relating to the patient's subsequent medical course must be submitted to Sponsor until the event has subsided or, in case of permanent impairment, until the event stabilizes and the overall clinical outcome has been ascertained.

Sponsor will ensure that all SAEs are reported to the relevant authorities and Principal Investigators according to relevant regulations and that product experience handling reporting requirements are followed.

Note: All device malfunctions should be reported to the Sponsor within 24 hours of knowledge.

8.5 Safety Monitoring

8.5.1 Data Safety Monitoring Board (DSMB)

All adverse events will be reported to the DSMB (Data and Safety Monitoring Board) and reviewed on a regular basis. The DSMB is an independent committee that will be comprised of at least three members, typically composed of at least two interventional cardiologists and a biostatistician. The DSMB members will not have any affiliation with the Sponsor, the Investigator, the Core lab or the clinical evaluation sites. The DSMB may request additional information as needed. Based on safety data, the DSMB may recommend that the Steering Committee modify or stop the study. All final decisions, however, regarding study modifications, rest with the Steering Committee. No formal statistical rule for stopping the study will be defined.

8.6 Adjudication of Events

8.6.1 Clinical Events Committee (CEC)

The Clinical Events Committee (CEC) is an independent adjudication body comprised of interventional and/or non-interventional cardiologists who are not participants in the clinical evaluation.

The CEC will review and adjudicate all endpoint related and major bleeding and vascular events according to the definitions provided in Appendix II. These definitions are based on the definitions developed by the Academic Research Consortium (ARC), as published in Circulation (Cutlip, D.E., et al., Clinical End Points in Coronary Stent Trials: A Case for Standardized Definitions. Circulation, 2007. 115: p. 2344-2351.)

- **Deaths**

The Clinical Events Committee (CEC) will adjudicate all patient deaths.

- **Myocardial Infarctions**

The CEC will adjudicate all possible cases of Q wave or non-Q wave myocardial infarctions for confirmation and outcomes/treatment.

- **Revascularizations**

For the SPIRIT WOMEN Single Arm Study, the CEC will be responsible for reviewing all angiograms to determine if a revascularization was performed, and if so, whether or not the revascularization was related to the target lesion, target vessel or non-target vessel. All revascularizations will be considered clinically indicated. For the randomized sub-study, the Angiographic Core Laboratory will evaluate whether the revascularization was performed and related to the target lesion, target vessel or non-target vessel and will provide QCA assessment of diameter stenosis. Based on that and clinical information the CEC will adjudicate whether the revascularization was clinically indicated or not.

- **Stent Thrombosis**

The CEC will adjudicate all possible cases of stent thromboses for confirmation and outcomes/treatment.

- **Vascular and Bleeding Complications**

The CEC will adjudicate all vascular and bleeding complications for confirmation and outcomes/treatment.

- **Other Adverse Events**

Adverse events other than the above will not be provided to the CEC for adjudication and will be reviewed by the Data Safety Monitoring Board (DSMB).

9 STATISTICAL ANALYSIS

9.1 Statistical Overview

This trial is composed of two parts:

SPIRIT WOMEN Single Arm Study:

A prospective, open-label, single arm, multi-center study evaluating performance of the XIENCE EECSS in the treatment of female patients according to IFU.

Randomized sub-study:

A prospective, single-blind, double arm, randomized, multi-center study comparing the XIENCE EECSS (XIENCE V EECSS and XIENCE PRIME EECSS) to the CYPHER SELECT™ Plus Sirolimus-eluting Coronary Stent.

The primary endpoint of this clinical evaluation is Adjudicated Composite rate of all Death, all Myocardial Infarction (MI) and Target Vessel Revascularization (TVR) at 1 year.

Descriptive analysis will be provided for the primary endpoint.

Hypothesis testing will be performed on the secondary endpoint in-stent Late Loss at 270 days. The null (H_0) and alternative (H_A) hypotheses are:

H_0: In-stent LL $_{XIENCE}$ - In-stent LL $_{CYPHER}$ $\geq \delta$
H_A: In-stent LL $_{XIENCE}$ - In-stent LL $_{CYPHER}$ $< \delta$

where δ is the non-inferiority margin.

9.2 Sample Size Justification

The SPIRIT WOMEN Clinical Evaluation aims to characterize the female population undergoing stent implantation and to precisely estimate the performance of XIENCE EECSS in this specific population. The primary endpoint is Adjudicated Composite rate of all Death, all Myocardial Infarction (MI) and Target Vessel Revascularization (TVR) at 1 year.

A sample size of 1550 patients enrolled in the SPIRIT WOMEN Single Arm Study will produce a narrow two-sided 95% confidence interval around the clinical endpoint estimates. The ½ width of the 2-sided confidence interval around the 1 year primary endpoint estimate will be between 1.5% and 1.7% assuming the true rate between 10% and 14%.

Moreover the pooled analysis including patients from the Single Arm Study and patients from the randomized sub-study who were randomized to XIENCE EECSS will report on approximately 1850 patients.

A drop-out rate of 3% is expected at 1-year.

Randomized Sub-study:

The overall sample size for the randomized sub-study is based on the main secondary endpoint of in-stent Late Loss at 270 days.

The sample size calculation is based on the following assumptions:

One-tailed non-inferiority test

$\alpha = 0.05$

Power = 90 %

Randomization ratio is 2 (XIENCE EECSS arm) : 1 (CYPHER arm)

True mean in-stent Late Loss is assumed to be equal for both treatment arms

Standard deviation is assumed to be 0.53 mm in both XIENCE EECSS arm and CYPHER arm

Non-inferiority margin delta (δ) of 0.17 mm

Given the above assumptions, approximately 252 subjects in the XIENCE EECSS arm and 126 subjects in the CYPHER arm will be required. In order to account for dropouts and to ensure enough angiographic data, approximately 450 subjects will be enrolled, of which approximately 300 will be in the XIENCE EECSS arm and approximately 150 in the CYPHER arm.

9.3 Analysis Populations

9.3.1 Intent to Treat Population

The intent-to-treat population for the randomized sub-study will consist of all patients (all lesions) randomized, regardless of the treatment actually received. Subjects will be analyzed in the treatment arm to which they were randomized.

Analysis for the Single Arm Study will be performed on the intent-to-treat population which will include all patients

1. who have signed the Patient Informed Consent
2. who have been registered in the ICON IVRS system

AND

3. who comply to at least one of the following:
   a. at least one XIENCE V stent has been implanted

b. a non-XIENCE V stent has been implanted after an intention to or an attempt made to implant a XIENCE V
c. no stent was implanted after an intention to or an attempt made to implant a XIENCE V

Patients not meeting the above criteria will not be required to undergo follow up as mandated by the CIP

Adverse event reported for patients who comply to criteria numbers 1 and 2 as mentioned above will be adjudicated but not included in the analysis even if criterion number 3 is not met.

Lesions for which there was no attempt to implant a XIENCE V EECS and lesions located in a bypass graft will not be considered in the analysis for the Single Arm Study.

For the pooled analysis the intent-to-treat population will consist of the intent-to-treat population in the SPIRIT WOMEN Single Arm Study and patients from the intent-to-treat population in the randomized sub-study who were randomized to XIENCE EECSS.

9.3.2 Per-Treatment Evaluable Population

In the randomized sub-study, the per-treatment evaluable population will consist of patients who have received the study device at the target lesion(s), who have no major procedural protocol deviations (e.g. stent implanted in a non-native coronary artery) other than those relating to treatment arm (randomized versus actually received) and for whom follow-up data is available. For example, if a patient had a stent placed in a saphenous vein graft, she would be excluded from the per-treatment evaluable population.

Analyses based on the per-treatment evaluable population will be "as treated". Patients will be included in the treatment arm corresponding to the study stent actually received.

Patients receiving a non-study stent on one or more of the target lesions or receiving several types of stents at target vessels will be excluded from this population.

No per-treatment evaluable population is defined for the SPIRIT WOMEN Single Arm Study.

9.4 Statistical Analyses

All analyses for SPIRIT WOMEN Single Arm Study and randomized sub-study will be performed on the intent-to-treat population. Additionally for the randomized sub-study the primary and secondary endpoints will be analyzed on the per-treatment evaluable population.

Primary Endpoint Analysis

Descriptive analysis will be provided for the primary endpoint Adjudicated Composite rate of all Death, all Myocardial Infarction (MI) and Target Vessel Revascularization (TVR) at 1 year. Counts, percentages and exact 95% confidence intervals will be calculated.

The primary endpoint will be evaluated on the SPIRIT WOMEN Single Arm Study intent-to-treat population, on the randomized sub-study intent-to-treat population and on the Single Arm Study intent-to-treat population pooled with the patients from the intent-to-treat population in the randomized sub-study who were randomized to XIENCE EECSS.

Randomized Sub-study – Analysis of In-stent Late Loss at 270 days

Statistical testing of the in-stent Late Loss endpoint will be one-tailed and performed at the 0.05 significance level for the comparison of XIENCE arm to CYPHER arm. The hypothesis testing will be based on all target lesions in the intent-to-treat population. The null hypothesis will be evaluated using a

non-inferiority test with asymptotic test statistic. If the correlation between lesions within patients is large, then repeated measures analysis will be considered.

If non-inferiority is shown and the in-stent Late Loss is lower in the XIENCE arm, then superiority analysis might be performed using a two-sided t-test at the 5% level.

In case the use of parametric tests is inappropriate due to the distribution assumptions violation, non-parametric tests will be considered.

The success of the randomized sub-study will be based on the non-inferiority testing of the in-stent Late Loss endpoint.

Analysis of Other Endpoints

Analysis of other study endpoints, descriptive in nature, will use adequate statistical methods.

For binary variables, counts, percentages, and exact 95% confidence intervals using Clopper-Pearson's method will be calculated. In addition, logistic regression might be performed to determine whether the data exhibit any trends in predicting angiographic binary restenosis and Adjudicated Composite rate of all Death, all Myocardial Infarction (MI) and Target Vessel Revascularization (TVR).

For continuous variables, means, standard deviations, and 95% confidence intervals for the mean using the Gaussian approximation will be calculated.

For time-to-event variables, survival curves will be constructed using Kaplan-Meier estimates, and log rank test results will be displayed for descriptive purposes only.

Additional analysis comparing different methods to achieve homeostasis will be performed.

Additional subgroup analyses will be described in the Statistical Analysis Plan.

9.5 Criteria for Early Termination of the Trial for Efficacy

No formal statistical rule for early termination of the trial for efficacy is defined.

9.6 Procedures for Accounting for Missing Data

Analyses will be based on available data, missing data excluded. Any unused or spurious data will be noted as appropriate in the final report.

9.7 Documentation of Changes to Statistical Plan

Any major changes to the statistical plan will be documented in an amendment to the clinical investigation plan. Less significant changes to the planned analyses will be documented in the final report.

10 DIRECT ACCESS TO SOURCE DATA/DOCUMENTS

The investigator/institution will permit direct access to source data/documents in order for clinical evaluation-related monitoring, audits, MEC review and regulatory inspections to be performed.

Patients providing informed consent are agreeing to allow Sponsor access and copying rights to pertinent information in their medical records concerning their participation in this clinical evaluation. The investigator will obtain, as part of the informed consent, permission for clinical evaluation monitors or regulatory authorities to review, in confidence, any records identifying the patients in this clinical

evaluation. This information may be shared with regulatory agencies; however, Sponsor undertakes not to otherwise release the patient's personal and private information.

11 QUALITY CONTROL AND QUALITY ASSURANCE

11.1 Selection of Clinical Sites and Investigators

Sponsor will select investigators qualified by training and experience, to participate in the investigation of the Clinical Investigation device. Sites will be selected based upon review of a recent site assessment and the qualifications of the principal investigator at the site. A listing of sites participating in this Clinical Evaluation is available upon request.

11.2 MEC Approval of Clinical Investigation Plan and Informed Consent

Medical Ethics Committee (MEC) approval for the clinical investigation plan and informed consent form will be obtained by the Investigator prior to participation in this clinical evaluation. The approval letter must be signed by the MEC chairman or authorized representative prior to the start of this clinical evaluation and must be provided to Sponsor. No changes will be made to the clinical investigation plan or informed consent form without Sponsor and MEC approval. Additionally, the investigator or representative will provide an MEC membership list to Sponsor.

Until the clinical evaluation is completed, the investigator will advise his/her MEC of the progress of this clinical evaluation, on a regular basis, according to MEC reporting requirements. If required, notification or submission to local Competent Authorities will be made through Sponsor local Regulatory contacts.

No investigative procedures other than those defined in this clinical investigation plan will be undertaken on the enrolled patients without the written agreement of the MEC and Sponsor.

11.2.1 Clinical Investigation Plan Amendments

Approved clinical investigation plan amendments will be provided to all investigators by Sponsor prior to implementing the amendment. The investigator will be responsible for notifying the MEC of the clinical investigation plan amendment (administrative changes) or obtaining MEC approval of the clinical investigation plan amendment (changes in patient care or safety), according to the instructions provided with the clinical investigation plan amendment by Sponsor. In the event an MEC approval is required, an MEC acknowledgement/approval of the clinical investigation plan amendment must be documented in writing prior to implementation of the clinical investigation plan amendment. Copies of this documentation must also be provided to Sponsor.

11.2.2 Clinical Investigation Plan Deviations

It is the investigator's responsibility to ensure that there are no deviations from the clinical investigation plan without notification and approval of Sponsor and full compliance with all established procedures of the MEC. The investigator will not deviate from the clinical investigation plan for any reason without prior written approval from Sponsor, except in cases of medical emergencies, when the deviation is necessary to eliminate an apparent immediate hazard to the patient. In that event, the investigator will notify Sponsor and document the deviation in the electronic CRF. The investigator will inform the MEC of all clinical investigation plan deviations according to each hospital's MEC requirements. In the event of any deviation from the clinical investigation plan, a clinical investigation plan deviation eCRF will be completed. The occurrence of clinical investigation plan deviations will be monitored by Sponsor for evaluation of investigator compliance to the clinical investigation plan and regulatory requirements.

11.2.3 Premature Termination of the Clinical Evaluation

Sponsor reserves the right to discontinue the Clinical Evaluation at any stage, with suitable written notice to the investigator. The investigator may also discontinue participation in the Clinical Evaluation with suitable written notice to Sponsor. Should either of these events occur, the investigator shall return all documents to the sponsor; provide a written statement as to why the premature termination has taken place; and notify the Medical Ethics Committee and the regulatory authority (if applicable). All applicable Clinical Investigation documents shall be subject to the same retention policy as detailed in the section of the protocol entitled '*13. DATA HANDLING AND RECORD KEEPING*.'

Follow-up for all enrolled patients will be as per protocol requirements.

11.2.4 Control of Study Devices and Equipment

Only commercially available shelf-stock will be used for the study. In countries where study devices are not commercially available, the study device will be treated as investigational device. Product expiration dates should be checked prior to implantation.

11.3 Training

11.3.1 Site Training

All investigators are required to attend Sponsor training sessions, which may be conducted at an Investigator's meeting, a site initiation visit or other appropriate training sessions. Training will include, but not be limited to, the investigational plan, investigational device usage, good clinical practices, electronic case report form completion, and clinical investigation personnel responsibilities. All investigator/clinical investigation personnel that are trained must sign a training log. No investigator/clinical investigation personnel will perform any clinical investigation-related procedures prior to signing a training log.

Prior to the initiation of the clinical investigation, the clinical investigation monitor or designee will visit each site where the clinical investigation is conducted. The clinical investigation monitor will ensure that clinical investigation site personnel are informed about and understand the clinical investigation requirements.

11.3.2 Compliance to Standards and Regulations

The clinical evaluation will be conducted in compliance with the clinical investigation plan, BS EN ISO 14155 –1/–2, Declaration of Helsinki and applicable regulatory requirements.

11.3.3 Monitoring

Monitoring will be performed during the study to ensure that compliance with the study CIP and applicable regulations is maintained, that data is collected in a timely, accurate and complete manner and that the investigator continues to have sufficient staff and facilities to conduct the study safely and effectively. Monitoring is performed in accordance with a pre defined monitoring plan.

11.3.4 Designated Monitors

Study monitors are individuals who are designated to oversee the progress of a study. These individuals are appropriately trained and qualified to monitor the progress of a clinical study. The study sponsor may designate additional monitors at any time during the study. The Sponsor should be contacted for additional information on the person(s) responsible for monitoring activities at the following address:

External Clinical Operations Manager
Abbott Vascular International BVBA
International Clinical Operations
Park Lane, Culliganlaan 2B
1831 Diegem
Belgium

11.3.5 Visits

Prior to initiating any procedure, the sponsor monitor (or delegate) will visit each investigator to ensure that the following criteria are met:

- The investigator understands and accepts the obligation to conduct the research study according to the CIP and applicable regulations, and has signed the Investigator Agreement.
- The investigator and his staff have sufficient time and facilities to conduct the study and that they have access to an adequate number of appropriate subjects to conduct the study.
- Additional monitoring will be conducted at regular intervals during the study to assess the continued compliance with the CIP and applicable regulations and maintenance of adequate study records. During monitoring visits, the monitor may assess the case report forms submitted by the investigator with respect to timeliness, adequacy, and accuracy by reviewing source documents for a representative number of case reports when necessary. The study administrative binder may also be checked for completeness.
- A final monitoring visit will be conducted before study closure to resolve any pending issue, including missing data, eventual corrections or incomplete study administrative binder documentation if such instances are encountered.
- For effective monitoring to take place, the investigator will allow the monitor designated by the sponsor to review completed case report forms and clinical records. The investigator will agree to dedicate an adequate amount of time to monitoring procedures

11.3.6 Securing Compliance

In the event of repeated non-compliance, as determined by the Sponsor, a Sponsor monitor or company representative will attempt to secure compliance by one or more of the following:

Visiting the investigator

Telephoning the investigator

Corresponding with the investigator

If an investigator is found to be repeatedly non-compliant with the signed agreement, the CIP or any other conditions of the study, Sponsor will either secure compliance or, at its sole discretion, terminate the investigator's participation in the study.

11.3.7 Quality Assurance Audit

A Sponsor representative or designee may request access to all clinical evaluation records, including source documentation, for inspection and duplication during a Quality Assurance audit. In the event that an investigator is contacted by a Regulatory Agency in relation to this clinical evaluation, the Investigator will notify Sponsor immediately. The investigator and research coordinator must be available to respond to reasonable requests and audit queries made during the audit process. The investigator must provide Sponsor with copies of all correspondence that may affect the review of the current clinical evaluation (e.g., Form FDA 483, Inspectional Observations, warning letters). Sponsor may provide any needed assistance in responding to regulatory audits.

11.4 The Steering Committee

The Steering Committee is assigned by the Sponsor and comprises the Principal Investigator, the Co-Principal Investigator and four/five dedicated members from the investigational sites as specified on the cover page of this protocol. The Sponsor will be represented by the Vice-President of International Clinical Operations or his designee, Scientific Advisor, Manager of Post marketing Programs, and the Clinical Project Manager.

The Steering Committee is responsible for overseeing the scientific and operational aspects of the study. This committee will meet regularly to monitor patient enrollment and non-compliance with the investigation plan at individual centers, to review and act upon recommendations of the Data and Safety Monitoring Board, and to determine policy regarding any publications arising from data generated from the performance of the study.

12 RISK ANALYSIS

12.1 Potential Risks (Risks from Cardiac Catheter, Stenting and Percutaneous Transcatheter Coronary Angioplasty)

There is extensive clinical and commercial experience worldwide with cardiac catheterization and interventional procedures and it is expected that the surgical and procedural risks will not be significantly different in this clinical study. The list of adverse events that may result from stent intervention includes:

Abrupt closure, acute myocardial infarction, allergic reaction to contrast, aneurysm, arterial perforation, arterial rupture, arteriovenous fistula, atrial arrhythmias (including bradycardia and tachycardia), bleeding complications which may require transfusions, coronary artery spasm, coronary or stent embolism, coronary artery or stent thrombosis or embolism, death, drug reactions to anti-platelet agents/contrast medium, emergency or non-emergent coronary artery bypass graft surgery, distal emboli (air, tissue or thrombotic), fever, hypersensitivity reactions, hypotension, hypertension, infection and pain at insertion site, injury to the coronary artery, ischemia (myocardial), myelosuppression, nausea and vomiting, palpitations, pseudoaneurysm, restenosis of the stented segment of the artery, stroke/cerebrovascular accident (CVA), total occlusion of the coronary artery, unstable or stable angina pectoris, vascular complications including entry site which may require vessel repair, ventricular arrhythmias including ventricular fibrillation, ventricular tachycardia, and vessel dissection.

A potential risk associated to the implantation of a stent system is an allergic reaction to the stent material.

12.2 Associated Risks of Everolimus

Everolimus is extensively metabolized by the cytochrome P4503A4 (CYP3A4) in the gut wall and liver and is a substrate for the countertransporter P-glycoprotein. Therefore, absorption and subsequent elimination of everolimus may be influenced by drugs that affect these pathways. Everolimus has also been shown to reduce the clearance of some prescription medications when it was administered orally along with cyclosporine (CsA). Formal drug interaction studies have not been performed with XIENCE Everolimus Eluting Coronary Stent System. Therefore, due consideration should be given to the potential for both systemic and local drug interactions in the vessel wall when deciding to place the XIENCE Everolimus Eluting Coronary Stent in a subject taking a drug with known interaction with everolimus.

Everolimus, when prescribed as an oral medication may interact with the following drugs[1]:

- CYP3A4 isozyme inhibitors (ketoconazole, itraconazole, ritonavor, erythromycin, clarithromycin, fluconazole, calcium channel blockers)
- Inducers of CYP3A4 isozyme (rifampin, rifabutin, carbamazepin, Phenobarbital, phenytoin)
- Antibiotics (ciprofloxacin, ofloxacin)
- Glucocorticoids
- HMGCoA reductase inhibitors (simavastatin, lorvastatin)
- Digoxin
- Cisapride (theoretical potential interaction)
- Sildenafil (Viagra®) (theoretical potential interaction)
- Antihistaminics (Terfanadine, astemizole)
- Grapefruit juice

Long term outcome for this permanent implant containing polymers and everolimus is unknown at present. Judicious selection of patients is necessary since the use of this device carries the associated risk of subacute thrombosis, vascular complications and / or bleeding events. Oral administration of everolimus in combination with cyclosporine have been associated with increased serum cholesterol and triglycerides. Therefore, patients should be monitored for changes in lipid profiles. Persons allergic to L-605 cobalt chromium alloy, acrylic or fluoro –polymers, or everolimus may suffer an allergic reaction to this implant.

12.3 Risk Management Procedures

It is recommended that patients will receive 75 mg of clopidogrel bisulfate (Plavix®) orally daily for a minimum of 6 months in the SPIRIT WOMEN Single Arm Study. Patients enrolled in the randomized sub-study will receive 75 mg of clopidogrel bisulfate (Plavix®) orally daily for a minimum of 6 months to reduce the risk of stent thrombosis and to provide extended protection to compensate for potentially delayed endothelialization after stent implant. It is also recommended that patients in the Single Arm Study receive aspirin (≥ 75 mg) orally daily for a minimum of 5 years following the procedure. All patients in the Randomized Sub-study will receive ≥ 75 mg of aspirin daily for a minimum of five years following the procedure.

---

[1] Certican® Investigator's Brochure. Novartis

Patients will be monitored closely throughout the clinical evaluation duration. Patients will be evaluated clinically at pre-determined time points to assess their clinical status and follow-up angiography will be performed in the randomized sub-study.

An independent Data Safety Monitoring Board (DSMB) will monitor safety throughout the clinical evaluation. Stopping rules will be discussed with the DSMB and applied for patient safety through enrollment.

12.4 Potential Benefits

The restenosis rate following stenting can range from 17.5% as seen in the PENTA Registry[xxiv] to 31.6% observed in the STRESS study[xxv]. Despite progress in the developments in coronary stenting, restenosis remains a major limiting factor in symptomatic relief for the patient and medical resource utilization, although it is not a major contributor to cardiac mortality.

Drug eluting stents have shown a reduction in the restenosis rate when compared to metallic stents. For example, in a recent randomized clinical study, in-segment restenosis rates were 8.9% in the drug eluting stent arm vs. 36.3% in the metallic stent control arm. Drug eluting stents have the potential to reduce restenosis and may have a positive effect on both patient outcomes and the costs of medical care. In the FUTURE studies comparing everolimus-eluting stents to metallic stents, the in-stent angiographic binary restenosis rate at 180 days were 0% in the everolimus stent arm and 9.1% in the control arm for FUTURE I and 0% versus 19.4% for FUTURE II[xxvi,xxvii].

In the SPIRIT FIRST study comparing the XIENCE V EECSS to the uncoated ML VISION® stent, the in-stent angiographic restenosis rate at 180 days were 0% and 25.9% (p=0.01), respectively, highlighting the differences between treatment and control arms. The in-segment binary restenosis rates were 4.3% and 33.3% (p=0.01), respectively, for the XIENCE V EECSS and control arms[xxviii].

In the SPIRIT II Trial which enrolled 300 patients, compared the XIENCE V EECSS to the TAXUS® EXPRESS2 PECS. All patients had clinical and angiographic follow-up with a subset of patients undergoing IVUS follow-up. The primary endpoint at six months was met with an in-stent LL of 0.11 ± 0.27 mm for the XIENCE V group compared to 0.36 mm for TAXUS® PECSS at 180 days, which represents a 72% reduction in late loss.

The long term effects and potential benefits of drug eluting stents are not yet known.

13 DATA HANDLING AND RECORD KEEPING

For the clinical evaluation duration, the investigator will maintain complete and accurate documentation including but not limited to:

- Correspondence with the sponsor, Ethics Committee and other investigators
- The signed CIP with any and all amendments
- Signed Informed Consent Forms
- Ethics Committee approval of the CIP and any amendment
- The investigators' agreement and the fee agreement (if applicable)
- The investigator's brochure / Instructions For Use
- The insurance certificate
- Laboratory certificate (or equivalent documentation)
- Complete and adequate source documents
- Financial Disclosure forms of all treating physicians
- Curricula vitae for the investigator and all co-investigators

- Laboratory Normal Reference Ranges
- Case Report Forms
- Adverse Event Reports
- Information regarding patient discontinuation or completion of the clinical evaluation.

13.1 Source Documentation

Regulations require that the Investigator maintain information in the patient's medical records which corroborate data collected on the case report forms. In order to comply with these regulatory requirements, at minimum, the following is a list of information that should be maintained.

- Medical history/physical condition of the patient before involvement in the clinical evaluation sufficient to verify clinical investigation plan entry criteria
- Dated and signed notes on the day of entry into the study including the study name (or abbreviation), treatment assigned and a statement that informed consent was obtained prior to any study-related procedure.
- Dated and signed notes from each patient visit with reference to the eCRFs for further information, if appropriate (for specific results of procedures and exams)
- Study required or endpoint related lab reports and ECGs, signed and dated for review, and annotated for clinically significant of out of range results.
- Adverse events reported and their resolution, including supporting documents such as discharge summaries, cath lab reports, ECGs, lab results
- Notes regarding clinical investigation plan-required and prescription medications taken during the clinical investigation (including start and stop dates)
- Patient's condition upon completion of or withdrawal from the clinical investigation

13.2 Case Report Form Completion

Primary data collection based on source-documented hospital chart reviews will be performed clearly and accurately by research coordinators (RC) at each clinical site trained on the clinical investigation plan and CRF completion. The data will be recorded on the electronic case report forms (eCRF). This data will be monitored by Sponsor appointed Study Monitors in an expedited fashion. Sponsor will provide clinical monitoring to include review of eCRFs and parity checks with the source documentation, including operator worksheets retained with eCRF documentation and hospital charts.

13.3 Record Retention

Study records must be kept for the maximum period of time required by the hospital and any relevant national regulations but no less than 15 years after the study completion date. All data and documents should be made available to the relevant authorities on request.

The sponsor will archive and retain all documents pertaining to the study for the lifetime of the device under evaluation.

14 PUBLICATION POLICY

The data and results from the Clinical investigation are the sole property of Sponsor. Sponsor shall have the right to access and use all data and results generated during the clinical investigation. The Investigator will not use the Clinical Investigation related data without the written consent of Sponsor for any other purpose than for Clinical Investigation completion or for generation of publication material, as referenced in the Clinical Site Agreement. The publication and/or presentation of results from a single Clinical Investigation site are not allowed until publication and/or presentation of the multi-center results. Sponsor acknowledges that the Principal Investigator(s) intend to publish a multi-center publication regarding the clinical investigation results. Sponsor must receive any proposed publication and/or presentation materials at least 60 days prior to the proposed date of the presentation or the initial submission of the proposed publication in order for the materials to be reviewed by Sponsor in compliance with Sponsor's publication policy set forth in the Clinical Site Agreement.

The Sponsor will be responsible for determining whether to register the Clinical Investigation on www.clinicaltrials.gov <<http://www.clinicaltrials.gov>> or any other clinical investigations, in accordance with the International Committee of Medical Journal Editors guidelines, or any other applicable guidelines. In the event Sponsor determines that the Trial should be registered, Sponsor shall be responsible for any such registration. Institution and/or Primary Investigator(s) shall not take any action to register the Trial.

The Steering Committee of the study will have full access to the data and will vouch for the data and the analysis.

15 Appendix 1: Declaration of Helsinki

WORLD MEDICAL ASSOCIATION DECLARATION OF HELSINKI
Ethical Principles for Medical Research Involving Human Subjects

Adopted by the 18th WMA General Assembly, Helsinki, Finland, June 1964, and amended by the:
29th WMA General Assembly, Tokyo, Japan, October 1975
35th WMA General Assembly, Venice, Italy, October 1983
41st WMA General Assembly, Hong Kong, September 1989
48th WMA General Assembly, Somerset West, Republic of South Africa, October 1996
52nd WMA General Assembly, Edinburgh, Scotland, October 2000
53th WMA General Assembly, Washington 2002 (Note of Clarification on paragraph 29 added)
55th WMA General Assembly, Tokyo 2004 (Note of Clarification on Paragraph 30 added)
59th WMA General Assembly, Seoul, October 2008

A. INTRODUCTION

1. The World Medical Association (WMA) has developed the Declaration of Helsinki as a statement of ethical principles for medical research involving human subjects**,** including research on identifiable human material and data.

The Declaration is intended to be read as a whole and each of its constituent paragraphs should not be applied without consideration of all other relevant paragraphs.

2. Although the Declaration is addressed primarily to physicians, the WMA encourages other participants in medical research involving human subjects to adopt these principles.

3. It is the duty of the physician to promote and safeguard the health of patients, including those who are involved in medical research. The physician's knowledge and conscience are dedicated to the fulfilment of this duty.

4. The Declaration of Geneva of the WMA binds the physician with the words, “The health of my patient will be my first consideration,” and the International Code of Medical Ethics declares that, “A physician shall act in the patient's best interest when providing medical care.”

5. Medical progress is based on research that ultimately must include studies involving human subjects. Populations that are underrepresented in medical research should be provided appropriate access to participation in research.

6. In medical research involving human subjects, the well-being of the individual research subject must take precedence over all other interests.

7. The primary purpose of medical research involving human subjects is to understand the causes, development and effects of diseases and improve preventive, diagnostic and therapeutic interventions (methods, procedures and treatments). Even the best current interventions must be evaluated continually through research for their safety, effectiveness, efficiency, accessibility and quality.

8. In medical practice and in medical research, most interventions involve risks and burdens.

9. Medical research is subject to ethical standards that promote respect for all human subjects and protect their health and rights. Some research populations are particularly vulnerable and need special protection. These include those who cannot give or refuse consent for themselves and those who may be vulnerable to coercion or undue influence.

10. Physicians should consider the ethical, legal and regulatory norms and standards for

research involving human subjects in their own countries as well as applicable international norms and standards. No national or international ethical, legal or regulatory requirement should reduce or eliminate any of the protections for research subjects set forth in this Declaration.

B. PRINCIPLES FOR ALL MEDICAL RESEARCH

11. It is the duty of physicians who participate in medical research to protect the life, health, dignity, integrity, right to self-determination, privacy, and confidentiality of personal information of research subjects.

12. Medical research involving human subjects must conform to generally accepted scientific principles, be based on a thorough knowledge of the scientific literature, other relevant sources of information, and adequate laboratory and, as appropriate, animal experimentation. The welfare of animals used for research must be respected.

13. Appropriate caution must be exercised in the conduct of medical research that may harm the environment.

14. The design and performance of each research study involving human subjects must be clearly described in a research protocol. The protocol should contain a statement of the ethical considerations involved and should indicate how the principles in this Declaration have been addressed. The protocol should include information regarding funding, sponsors, institutional affiliations, other potential conflicts of interest, incentives for subjects and provisions for treating and/or compensating subjects who are harmed as a consequence of participation in the research study. The protocol should describe arrangements for post-study access by study subjects to interventions identified as beneficial in the study or access to other appropriate care or benefits.

15. The research protocol must be submitted for consideration, comment, guidance and approval to a research ethics committee before the study begins. This committee must be independent of the researcher, the sponsor and any other undue influence. It must take into consideration the laws and regulations of the country or countries in which the research is to be performed as well as applicable international norms and standards but these must not be allowed to reduce or eliminate any of the protections for research subjects set forth in this Declaration. The committee must have the right to monitor ongoing studies. The researcher must provide monitoring information to the committee, especially information about any serious adverse events. No change to the protocol may be made without consideration and approval by the committee.

16. Medical research involving human subjects must be conducted only by individuals with the appropriate scientific training and qualifications. Research on patients or healthy volunteers requires the supervision of a competent and appropriately qualified physician or other health care professional. The responsibility for the protection of research subjects must always rest with the physician or other health care professional and never the research subjects, even though they have given consent.

17. Medical research involving a disadvantaged or vulnerable population or community is only justified if the research is responsive to the health needs and priorities of this population or community and if there is a reasonable likelihood that this population or community stands to benefit from the results of the research.

18. Every medical research study involving human subjects must be preceded by careful assessment of predictable risks and burdens to the individuals and communities involved in the research in comparison with foreseeable benefits to them and to other individuals or communities affected by the condition under investigation.

19. Every clinical trial must be registered in a publicly accessible database before

recruitment of the first subject.
20. Physicians may not participate in a research study involving human subjects unless they are confident that the risks involved have been adequately assessed and can be satisfactorily managed. Physicians must immediately stop a study when the risks are found to outweigh the potential benefits or when there is conclusive proof of positive and beneficial results.
21. Medical research involving human subjects may only be conducted if the importance of the objective outweighs the inherent risks and burdens to the research subjects.
22. Participation by competent individuals as subjects in medical research must be voluntary. Although it may be appropriate to consult family members or community leaders, no competent individual may be enrolled in a research study unless he or she freely agrees.
23. Every precaution must be taken to protect the privacy of research subjects and the confidentiality of their personal information and to minimize the impact of the study on their physical, mental and social integrity.
24. In medical research involving competent human subjects, each potential subject must be adequately informed of the aims, methods, sources of funding, any possible conflicts of interest, institutional affiliations of the researcher, the anticipated benefits and potential risks of the study and the discomfort it may entail, and any other relevant aspects of the study. The potential subject must be informed of the right to refuse to participate in the study or to withdraw consent to participate at any time without reprisal. Special attention should be given to the specific information needs of individual potential subjects as well as to the methods used to deliver the information. After ensuring that the potential subject has understood the information, the physician or another appropriately qualified individual must then seek the potential subject's freely-given informed consent, preferably in writing. If the consent cannot be expressed in writing, the non-written consent must be formally documented and witnessed.
25. For medical research using identifiable human material or data, physicians must normally seek consent for the collection, analysis, storage and/or reuse. There may be situations where consent would be impossible or impractical to obtain for such research or would pose a threat to the validity of the research. In such situations the research may be done only after consideration and approval of a research ethics committee.
26. When seeking informed consent for participation in a research study the physician should be particularly cautious if the potential subject is in a dependent relationship with the physician or may consent under duress. In such situations the informed consent should be sought by an appropriately qualified individual who is completely independent of this relationship.
27. For a potential research subject who is incompetent, the physician must seek informed consent from the legally authorized representative. These individuals must not be included in a research study that has no likelihood of benefit for them unless it is intended to promote the health of the population represented by the potential subject, the research cannot instead be performed with competent persons, and the research entails only minimal risk and minimal burden.
28. When a potential research subject who is deemed incompetent is able to give assent to decisions about participation in research, the physician must seek that assent in addition to the consent of the legally authorized representative. The potential subject's dissent should be respected.
29. Research involving subjects who are physically or mentally incapable of giving

consent, for example, unconscious patients, may be done only if the physical or mental condition that prevents giving informed consent is a necessary characteristic of the research population. In such circumstances the physician should seek informed consent from the legally authorized representative. If no such representative is available and if the research cannot be delayed, the study may proceed without informed consent provided that the specific reasons for involving subjects with a condition that renders them unable to give informed consent have been stated in the research protocol and the study has been approved by a research ethics committee. Consent to remain in the research should be obtained as soon as possible from the subject or a legally authorized representative.

30. Authors, editors and publishers all have ethical obligations with regard to the publication of the results of research. Authors have a duty to make publicly available the results of their research on human subjects and are accountable for the completeness and accuracy of their reports. They should adhere to accepted guidelines for ethical reporting. Negative and inconclusive as well as positive results should be published or otherwise made publicly available. Sources of funding, institutional affiliations and conflicts of interest should be declared in the publication. Reports of research not in accordance with the principles of this Declaration should not be accepted for publication.

C. ADDITIONAL PRINCIPLES FOR MEDICAL RESEARCH COMBINED WITH MEDICAL CARE

31. The physician may combine medical research with medical care only to the extent that the research is justified by its potential preventive, diagnostic or therapeutic value and if the physician has good reason to believe that participation in the research study will not adversely affect the health of the patients who serve as research subjects.

32. The benefits, risks, burdens and effectiveness of a new intervention must be tested against those of the best current proven intervention, except in the following circumstances:

• The use of placebo, or no treatment, is acceptable in studies where no current proven intervention exists; or

• Where for compelling and scientifically sound methodological reasons the use of placebo is necessary to determine the efficacy or safety of an intervention and the patients who receive placebo or no treatment will not be subject to any risk of serious or irreversible harm. Extreme care must be taken to avoid abuse of this option.

33. At the conclusion of the study, patients entered into the study are entitled to be informed about the outcome of the study and to share any benefits that result from it, for example, access to interventions identified as beneficial in the study or to other appropriate care or benefits.

34. The physician must fully inform the patient which aspects of the care are related to the research. The refusal of a patient to participate in a study or the patient's decision to withdraw from the study must never interfere with the patient-physician relationship.

35. In the treatment of a patient, where proven interventions do not exist or have been ineffective, the physician, after seeking expert advice, with informed consent from the patient or a legally authorized representative, may use an unproven intervention if in the physician's judgement it offers hope of saving life, re-establishing health or alleviating suffering. Where possible, this intervention should be made the object of research,

designed to evaluate its safety and efficacy. In all cases, new information should be recorded and, where appropriate, made publicly available.

16 Appendix II Definitions

Abrupt Closure (Acute Closure)
Occurrence of new severely reduced flow (TIMI grade 0 or 1) within the target vessel during the index procedure that persists and requires rescue by a non-assigned treatment strategy (including emergency surgery), or results in myocardial infarction or death. Abrupt closure requires proven association with a mechanical dissection of the treatment lesion or instrumented vessel, coronary thrombus, or severe spasm. Abrupt closure does not connote "no reflow" (due to microvascular flow limitation), in which the vessel is patent but had reduced flow. Abrupt closure also does not connote transient closure with reduced flow in which the index treatment application reversed the closure.

- **Subabrupt Closure**
  Abrupt closure that occurs after the index procedure is completed (and the patient left the catheterization laboratory) and before the 30-day follow-up endpoint.

- **Threatened Abrupt Closure**
  Defined as a grade B dissection and ≥ 50% diameter stenosis or any dissection of grade C or higher.

ACC/AHA Classification Scheme of Coronary Lesions: Lesion-Specific Characteristics

**Type A Lesions (High Success, >85%; Low Risk)**	
• Discrete (< 10 mm length)	• Little or no calcification
• Concentric	• Less than totally occlusive
• Readily accessible	• Not ostial in location
• Nonangulated segment, < 45°	• No major branch involvement
• Smooth contour	• Absence of thrombus
**Type B Lesions* (Moderate Success, 60-85%; Moderate risk)**	
• Tubular (10-20 mm length)	• Moderate-to-heavy calcification
• Eccentric	• Total occlusions < 3 mo old
• Moderate tortuosity of proximal segment	• Ostial in location
• Moderately angulated segment, > 45°, < 90°	• Bifurcation lesions requiring double guide wires
• Irregular contour	• Some thrombus present
* Type B1 lesions: One adverse characteristic<br>* Type B2 lesions: ≥ two adverse characteristics	
**Type C Lesions (Low Success, <60%; High Risk)**	
• Diffuse (> 2 cm length)	• Total occlusions > 3 mo old
• Excessive tortuosity of proximal segment	• Inability to protect major side branches
• Extremely angulated segments > 90°	• Degenerated vein grafts with friable lesions

Acute Success
Acute Success is classified according to the following definitions:

Clinical Device Success
Successful delivery and deployment of the study stent (in overlapping stent setting a successful delivery

and deployment of the first and second study stent) at the intended target lesion and successful withdrawal of the stent delivery system with attainment of final residual stenosis of less than 50% of the target lesion by visual estimation, without use of a device outside the assigned treatment strategy. Standard pre-dilation catheters and post-dilatation catheters (if applicable) may be used. Bailout patients will be included as clinical device success only if the above criteria for clinical device success are met.

Clinical Procedure Success
Successful delivery and deployment of the study stent or stents at the intended target lesion and successful withdrawal of the stent delivery system with attainment of final residual stenosis of less than 50% by visual estimation, using any adjunctive device without the occurrence of cardiac death, MI attributed to the target vessel and/or CI-TLR during the hospital stay with a maximum of first seven days post index procedure. In multiple lesions setting each lesion must meet clinical procedure success.

Adverse Device Effect
Any untoward and unintended response to a medical device. This definition includes any event resulting from insufficiencies or inadequacies in the instructions for use or the deployment of the device. It also includes any event that is a result of a user error.

Adverse Event
Any untoward medical occurrence in a patient. This definition does not imply that there is a relationship between the adverse event and the device under investigation.

Aneurysm
An abnormal expansion or protrusion of a coronary blood vessel resulting from a disease or weakening of the vessel's wall (all three layers) that exceeds the RVD of the vessel by 1.5 times.

Angina Pectoris
Braunwald Classification of Unstable Angina:

I. New onset of severe or accelerated angina. Patients with new onset (< 2 months in duration) exertional angina pectoris that is severe or frequent (> 3 episodes/day) or patients with chronic stable angina who develop accelerated angina (that is, angina distinctly more frequent, severe, longer in duration, or precipitated by distinctly less exertion than previously) but who have not experienced pain at rest during the preceding 2 months.

II. Angina at rest, subacute. Patients with one or more episodes of angina at rest during the preceding month but not within the preceding 48 hours.

III. Angina at rest, acute. Patients with one or more episodes of angina at rest within the preceding 48 hours.

Canadian Cardiovascular Society (CCS) Classification of Stable Angina:

I. Ordinary physical activity does not cause angina; for example walking or climbing stairs, angina occurs with strenuous or rapid or prolonged exertion at work or recreation.

II. Slight limitation of ordinary activity; for example, angina occurs walking or stair climbing after meals, in cold, in wind, under emotional stress or only during the few hours after awakening, walking more than two blocks on the level or climbing more than one flight of ordinary stairs at a normal pace and in normal conditions.

III. Marked limitation of ordinary activity; for example, angina occurs walking one or two blocks on the level or climbing one flight of stairs in normal conditions and at a normal pace.

IV. Inability to carry on any physical activity without discomfort - angina syndrome may be present at rest.

Angiographic Binary Restenosis Rate

Percent of patients with a follow-up percent diameter stenosis of ≥ 50% per Quantitative Coronary Angiogram.

Bifurcation Lesion

A lesion involving a main vessel and a side branch of a main vessel.

Bleeding (Hemorrhagic) Complications

These may include hematoma requiring transfusion or surgical repair, and any bleeding event associated with hemoglobin drop > 5 g/dl or requiring transfusion or surgical repair (e.g., retroperitoneal bleed, GI bleed, access site bleed).

Bleeding will be classified by the TIMI hemorrhage classification and according to the ACC National Cardiovascular Data Registry scheme as defined below:
A. Major: Clinically significant overt signs of hemorrhage and/or intracranial/intraocular bleeding is present and associated with a drop in hemoglobin of >5g/dL (or when hemoglobin is not available, an absolute drop in hematocrit of >15%.
B**. Minor:** Any bleeding that does not fit the criteria specified above in the 'Major' classification.

Chronic concomitant Medications

Medication that has been:

prescribed or over the counter (OTC) that has been taken or will continue to be taken regularly for at least a period of 6 months, or

is required to be taken indefinitely by the patient, or

prescribed or OTC that has been taken multiple times (each time for at least 6 months)

Chronic Occlusion

An occlusion presumed to have been present for at least one month prior to the procedure.

- Total Occlusion:
- An occlusion with no ante grade filling of contrast to the distal segment (TIMI grade 0)
- Sub-total Occlusion:
- TIMI grade I, and with collateral filling of the distal segment

Clinically-indicated Target Lesion Revascularization (CI-TLR):

In the patient cohorts assigned to clinical follow-up only (such as the SPIRIT WOMEN Single Arm Study), all revascularizations are considered clinically indicated.

A revascularization is clinically indicated if angiography at follow-up shows a percent diameter stenosis ≥ 50% (QCA assessment) **and** if one of the following occurs:

1. A positive history of recurrent angina pectoris presumably related to the target vessel.
2. Objective signs of ischemia at rest (ECG changes) or during exercise test (or equivalent) presumably related to the target vessel.

3. Abnormal results of any invasive functional diagnostic test (e.g. Doppler flow velocity reserve, fractional flow reserve). The results of the test must be documented in the Case Report Form.

A TLR with a diameter stenosis ≥70% (QCA) in the absence of the above mentioned ischemic signs or symptoms is also considered clinically indicated.

Comment: clinically indicated is a documented, provisional decision to re-intervene based on clinical symptoms and/or results of non invasive functional testing, before any coronary imaging. These criteria are intended to determine clinical indications for reintervention in patients undergoing routine angiographic follow-up. In the patient cohorts assigned to clinical follow-up only, or in studies without routine angiographic follow-up, all revascularizations are considered clinically indicated.

Not Clinically indicated TLRs are reinterventions for:

1. all stenoses <50%* in the presence or absence of ischemic signs or symptoms;
2. all stenoses ≥50% but <70% without ischemic signs or symptoms.

Clinically-indicated Target Vessel Revascularization (CI-TVR):

In the patient cohorts assigned to clinical follow-up only (such as the SPIRIT WOMEN Single Arm Study), all revascularizations are considered clinically indicated.

A revascularization is clinically indicated if angiography at follow-up shows a percent diameter stenosis ≥ 50% (QCA assessment) and if one of the following occurs:

1. A positive history of recurrent angina pectoris presumably related to the target vessel.
2. Objective signs of ischemia at rest (ECG changes) or during exercise test (or equivalent) presumably related to the target vessel.
3. Abnormal results of any invasive functional diagnostic test (e.g. Doppler flow velocity reserve, fractional flow reserve). The results of the test must be documented in the Case Report Form.

A TVR with a diameter stenosis ≥70% (QCA) in the absence of the above mentioned ischemic signs or symptoms is also considered clinically indicated.

Not Clinically indicated TVRs are reinterventions for:

1. all stenoses <50% in the presence or absence of ischemic signs or symptoms;
2. all stenoses ≥50% but <70% without ischemic signs or symptoms.

Coronary Artery Bypass Graft Surgery (CABG)

- Acute CABG is defined as immediate transfer from the cath lab to the operative room for emergent bypass surgery during the initial treatment phase.
- CABG during follow-up is only considered as a Clinical-indicated Target Lesion Revascularization if coronary angiography indicates a diameter of stenosis ≥ 50% of the stented coronary segment associated with one of the following conditions:
    1. A positive history of recurrent angina pectoris presumably related to the target vessel.
    2. Objective signs of ischemia at rest (ECG changes) or during exercise test (or equivalent) presumably related to the target vessel.
    3. Abnormal results of any invasive functional diagnostic test (e.g. Doppler flow velocity reserve, fractional flow reserve). The results of the test must be documented in the Case Report Form.

Death

Death defined by the Academic Research Consortium is as follows:

All death is considered to be cardiac death unless an unequivocal noncardiac cause can be established. Specifically, any unexpected death even in patients with coexisting potentially fatal noncardiac disease (eg, cancer, infection) should be classified as cardiac.

Cardiac death

Any death due to proximate cardiac cause (eg, myocardial infarction, low-output failure, fatal arrhythmia), unwitnessed death and death of unknown cause, and all procedure-related deaths, including those related to concomitant treatment, will be classified as cardiac death.

Vascular death

Death caused by noncoronary vascular causes, such as cerebrovascular disease, pulmonary embolism, ruptured aortic aneurysm, dissecting aneurysm, or other vascular diseases.

Non-cardiovascular death

Any death not covered by the above definitions, such as death caused by infection, malignancy, sepsis, pulmonary causes, accident, suicide, or trauma.

Device Malfunction

A malfunction is a failure of a device to meet its performance specifications or otherwise perform as intended. Performance specifications include all claims made in the labeling of the device. The intended performance of a device refers to the intended use for which the device is labeled or marketed.

Dissection

National Heart, Lung, and Blood Institute (NHLBI) Dissection Classification System:

A. Minor radiolucencies within the lumen during contrast injection with no persistence after dye clearance.

B. Parallel tracts or double lumen separated by a radiolucent area during contrast injection with no persistence after dye clearance.

C. Extraluminal cap with persistence of contrast after dye clearance from the lumen.

D. Spiral luminal filling defects.

E. New persistent filling defects.

F. Non-A-E types that lead to impaired flow or total occlusion.

Note: Type E and F dissections may represent thrombus.

Hematoma

Hemorrhagic event requiring transfusion, prolongation of hospitalization or an intervention.

In-Stent

Within the margins of the stent.

In-Segment

Within the margins of the stent and 5 mm proximal and 5 mm distal to the stent.

Intent To Treat (ITT) Population

The intent-to-treat population for the randomized sub-study will consist of all patients (all lesions) randomized, regardless of the treatment actually received. Subjects will be analyzed in the treatment arm to which they were randomized.

Analysis for the Single Arm Study will be performed on the intent-to-treat population which will include all patients:

1. who have signed the Patient Informed Consent
2. who have been registered in the ICON IVRS system
AND
3. who comply to at least one of the following:
   a. at least one XIENCE V EECS has been implanted
   b. a non-XIENCE V EECS has been implanted after an intention to or an attempt made to implant a XIENCE V EECS

   c. no stent was implanted after an intention to or an attempt made to implant a XIENCE V EECS

Lesions for which there was no attempt to implant a XIENCE V EECS and lesions located in a bypass graft will not be considered in the analysis for the Single Arm Study.

For the pooled analysis the intent-to-treat population will consist of the intent-to-treat population in the SPIRIT WOMEN Single Arm Study and patients from the intent-to-treat population in the randomized sub-study who were randomized to XIENCE EECSS.

Late Loss (LL)
General definition: Calculated as MLD post-procedure – MLD at follow-up

- **In-segment Late Loss:** in-segment MLD post-procedure – in segment MLD at follow-up
- **In-stent Late Loss:** in-stent MLD post-procedure – in-stent MLD at follow-up
- **Proximal Late Loss:** proximal MLD post-procedure – proximal MLD at follow-up (proximal defined as within 5 mm proximal to stent placement)
- **Distal Late Loss:** distal MLD post-procedure – distal MLD at follow-up (distal defined as within 5 mm distal to stent placement)

Major Epicardial Vessels
Left anterior descending artery (LAD) with septal and diagonal branches;

Left circumflex artery (LCX) with obtuse marginal and/or ramus intermedius branches;

Right coronary artery (RCA) and any of its branches.

Metabolic Syndrome
Metabolic syndrome, (also, syndrome x, insulin resistance syndrome) is a combination of medical disorders that affect a large number of people. If you have three or more of the following five conditions, you are diagnosed as metabolic syndrome:

- Increased waist circumference (≥102 cm in men and ≥88 cm in women), indicating central obesity
- Elevated triglycerides (≥150 mg/dL or 1.7 mmol/l)
- Decreased HDL cholesterol (<40 mg/dL for men, <50 mg/dL for women)
- Blood pressure above 130/85 or active treatment for hypertension
- Glucose levels above 100 mg/dL (a symptom of diabetes)

Minimum Lumen Diameter (MLD)
The average of two orthogonal views (when possible) of the narrowest point within the area of assessment – in lesion, in stent or in segment. MLD is visually estimated during angiography by the Investigator; it is measured during QCA by the Angiographic Core Lab.

Myocardial Infarction (MI)

Myocardial Infarction Classification and Criteria for Diagnosis is defined by the Academic Research Consortium as follows:

**Classification**	**Biomarker Criteria***	**Additional Criteria**
Periprocedural PCI	Troponin > 3 x URL or CK-MB > 3 x URL	Baseline value <URL
Periprocedural CABG	Troponin > 5 x URL or CK-MB > 5 x URL	Baseline value <URL and any of the following: new pathologic Q waves or LBBB, new native or graft vessel occlusion, imaging evidence of loss of viable myocardium
Spontaneous	Troponin > URL or CK-MB > URL	
Sudden death	Death before biomarkers obtained or before expected to be elevated	Symptoms suggestive of ischemia and any of the following: new ST elevation or LBBB, documented thrombus by angiography or autopsy
Reinfarction	Stable or decreasing values on 2 samples with a 20% increase 3 to 6 hours after second sample	If biomarkers increasing or peak not reached then insufficient data to diagnose recurrent MI

URL = Upper Reference Limit (defined 99th percentile of normal reference range);
LBBB = Left Bundle-branch Block
* Baseline biomarker value requiring before study procedure and presumes a typical rise and fall

- **Periprocedural MI After PCI**

  The periprocedural period includes the first 48 hours after percutaneous coronary intervention.

- **Periprocedural MI After CABG**

  The periprocedural period includes the first 72 hours after coronary artery bypass CABG grafting.

- **Spontaneous MI**

  MI after the periprocedural period may be secondary to late stent complications or progression of native disease.

  Performance of ECG and angiography supports adjudication to either a target or non-target vessel in most cases.

  With the unique issues and pathophysiological mechanisms associated with these later events as well as the documented adverse impact on short-and long-term prognosis, a more sensitive definition than for periprocedural MI of any elevation of troponin above the upper range limit is used. All late events that are not associated with a revascularization procedure simply as spontaneous.

- **Electrocardiographic Classification**

  Within this category Q-wave MI and Non Q-wave MI are distinguished as follows:

  – Q-wave MI: Development of new pathologicals in 2 or more contiguous leads (according to the Minnesota code or Novacode) with or without post-procedure CK or CK-MB levels elevated above normal.

– Non Q-wave MI: All MIs not classified as Q-wave.

- **Relation to the Target Vessel**

  All infarcts that cannot be clearly attributed to a vessel other than the target vessel will be considered related to the target vessel.

No-reflow
An acute reduction in coronary flow (TIMI grade 0-1) in the absence of dissection, thrombus, spasm, or high-grade residual stenosis at the original target lesion. Also see 'Acute Closure'.

Percent Diameter Stenosis
The value calculated as 100 * (1 - MLD/RVD) using the mean values from two orthogonal views (when possible) by QCA.

Percutaneous Coronary Intervention (PCI)
Refers to all interventional cardiology methods for treatment of coronary artery disease.

Persisting Dissection
Dissection at follow-up that was present post-procedure.

Planned Staged Procedure

A planned staged procedure is defined as the treatment of baseline lesions during additional cardiac catheterization procedures following the index procedure. The definition is limited to procedures that are planned during/prior to index procedure. If worsening/exacerbation of patient condition occurs at the time of staged procedure or leads to rescheduling of the staged procedure, or a new clinical condition occurs during the staged procedure, the condition should be reported as an adverse event. The treatment of the baseline lesions at staged procedure will not be considered as a revascularization.

Q-wave Myocardial Infarction (QMI)
Development of new pathological Q waves in 2 or more contiguous leads (according to the Minnesota Code as assessed by the ECG core laboratory) with or without post-procedure CK or CK-MB levels elevated above normal.

Reference Vessel Diameter (RVD)
An approximation of the diameter of the vessel at the location of the target lesion. RVD is visually estimated during angiography by the Investigator and it is measured during QCA by the Angiographic Core Laboratory

Restenosis
Re-narrowing of the artery following the removal or reduction of a previous narrowing.

Revascularization:

Revascularization is defined by the Academic Research Consortium [28] as follows:

- **Target Lesion Revascularization (TLR)**

  Target lesion revascularization is defined as any repeat percutaneous intervention of the target lesion or bypass surgery of the target vessel performed for restenosis or other complication of the target lesion. All target lesion revascularizations should be classified prospectively as clinically indicated* or not clinically indicated by the investigator prior to repeat angiography. An independent angiographic core laboratory should verify that the severity of percent diameter stenosis meets requirements for clinical indication and will overrule in cases where investigator reports are not in agreement. The target lesion is defined as the treated segment from 5 mm proximal to the stent and to 5 mm distal to the stent.

- **Target Vessel Revascularization (TVR)**

Target vessel revascularization is defined as any repeat percutaneous intervention or surgical bypass of any segment of the target vessel. The target vessel is defined as the entire major coronary vessel proximal and distal to the target lesion, which includes upstream and downstream branches and the target lesion itself.

- **Non Target Lesion Revascularization (non-TLR)**

Any revascularization in a lesion other than the target lesion is considered a non target lesion revascularization.

- **Non Target Vessel Revascularization (non-TVR)**

Any revascularization in a vessel other than the target vessel is considered a non-target vessel revascularization.

***Clinically indicated revascularization**
A revascularization is considered clinically indicated if angiography at follow-up shows a percent diameter stenosis ≥ 50% (core laboratory quantitative coronary angiography assessment) and if one of the following occurs:
(1) A positive history of recurrent angina pectoris, presumably related to the target vessel;
(2) Objective signs of ischemia at rest (ECG changes) or during exercise test (or equivalent), presumably related to the target vessel;
(3) Abnormal results of any invasive functional diagnostic test (eg, Doppler flow velocity reserve, fractional flow reserve);
(4) A TLR or TVR with a diameter stenosis ≥ 70% even in the absence of the above-mentioned ischemic signs or symptoms.

Serious Adverse Device Effect
Adverse device effect that has resulted in any of the consequences characteristic of a serious adverse event or that might have led to any of these consequences if suitable action had not been taken or intervention had not been made or if circumstances had been less opportune.

Serious Adverse Event (SAE)

Adverse event that:

- ➢ led to death,
- ➢ led to a serious deterioration in the health of the patient that:
  - resulted in a life-threatening illness or injury,
  - resulted in a permanent impairment of a body structure or a body function,
  - required in-patient hospitalization or prolongation of existing hospitalization
  - resulted in medical or surgical intervention to prevent permanent impairment to body structure or a body function
- ➢ led to fetal distress, fetal death or a congenital abnormality or birth defect

Stent Thrombosis

Stent thrombosis is defined and discussed by the Academic Research Consortium as follows:

Stent thrombosis should be reported as a cumulative value at the different time points and with the different separate time points.

Time 0 is defined as the time point after the guiding catheter has been removed and the subject left the catheterization laboratory.

Timing

Acute stent thrombosis*	0-24 hours post stent implantation
Subacute stent thrombosis*	> 24 hours-30 days post stent implantation
Late stent thrombosis†	> 30 days-1 year post stent implantation
Very late stent thrombosis†	> 1 year post stent implantation

* Acute/subacute can also be replaced by early stent thrombosis. Early stent thrombosis (0-30 days) –*this definition is currently used in the community*.

† Including "primary" as well as "secondary" late stent thrombosis; "secondary" late stent thrombosis is a stent thrombosis after a target segment revascularization.

Stent Thrombosis Categories: a) Definite b) Probable, and c) Possible

a) Definite stent thrombosis

Definite stent thrombosis is considered to have occurred by either angiographic or pathologic confirmation.

Angiographic confirmation of stent thrombosis*

The presence of a thrombus† that originates in the stent or in the segment 5 mm proximal or distal to the stent and presence of at least 1 of the following criteria within a 48-hour time window:

- Acute onset of ischemic symptoms at rest
- New ischemic ECG changes that suggest acute ischemia
- Typical rise and fall in cardiac biomarkers (refer to definition of spontaneous MI)
- Nonocclusive thrombus
  -Intracoronary thrombus is defined as a (spheric, ovoid, or irregular) noncalcified filling defect or lucency surrounded by contrast material (on 3 sides or within a coronary stenosis) seen in multiple projections, or persistence of contrast material within the lumen, or a visible embolization of intraluminal material downstream.
- Occlusive thrombus
  -TIMI 0 or TIMI 1 intrastent or proximal to a stent up to the most adjacent proximal side branch or main branch (if originates from the side branch).

*****The incidental angiographic documentation of stent occlusion in the absence of clinical signs or symptoms is not considered a confirmed stent thrombosis (silent occlusion).

†Intracoronary thrombus.

Pathological confirmation of stent thrombosis

Evidence of recent thrombus within the stent determined at autopsy or via examination of tissue retrieved following thrombectomy.

b) Probable stent thrombosis

Clinical definition of probable stent thrombosis is considered to have occurred after intracoronary stenting in the following cases:

- Any unexplained death within the first 30 days‡
- Irrespective of the time after the index procedure, any MI that is related to documented acute ischemia in the territory of the implanted stent without angiographic confirmation of stent thrombosis and in the absence of any other obvious cause

‡ For studies with ST-elevation MI population, one may consider the exclusion of unexplained death within 30 days as evidence of probable stent thrombosis.

c) Possible stent thrombosis
Clinical definition of possible stent thrombosis is considered to have occurred with any unexplained death from 30 days after intracoronary stenting until end of trial follow-up.

Successful pre-dilation
Pre-dilation has been successfully completed without complications if all of the following apply:

- diameter stenosis < 50%
- TIMI Grade-3 flow
- lesion length still within the requirements of the CIP
- no angiographic complications or prolonged chest pain

Stroke or Cerebrovascular Accident (CVA)
The occurrence of cerebral infarction (ischemic stroke) and intracerebral hemorrhage and subarachnoid hemorrhage (hemorrhagic stroke).

Target Lesion
The target lesion is the treated lesion starting 5mm proximal to the stent and is ending 5mm distal to the stent. Any lesion planned to be treated is considered as target lesion.

TIMI (Thrombosis In Myocardial Infarction) Flow Grades[xxix]

0. No contrast flow through the stenosis.
1. A small amount of contrast flows through the stenosis but fails to fully opacify the artery beyond.
2. Contrast material flows through the stenosis to opacify the terminal artery segment. However, contrast enters the terminal segment perceptibly more slowly than more proximal segments. Alternatively, contrast material clears from a segment distal to a stenosis noticeably more slowly than from a comparable segment not preceded by a significant stenosis.
3. Anterograde flow into the terminal coronary artery segment through a stenosis is as prompt as anterograde flow into a comparable segment proximal to the stenosis. Contrast material clears as rapidly from the distal segment as from an uninvolved, more proximal segment.

Transient Ischemic Attack (TIA)
A brief episode of neurologic dysfunction caused by focal brain or retinal ischemia, with clinical symptoms typically lasting less than 1 hour, and without evidence of acute infarction.

Vascular Complications
These may include pseudoaneurysm, arteriovenous fistula, CVA or TIA, and peripheral ischemia or nerve injury.

17 Appendix III Acronyms and Abbreviation

Acronym/ Abbreviation	Term
%DS	Percent Diameter Stenosis
%VO	Percent Volume Obstruction (In-Stent)
ABR	Angiographic Binary Restenosis Rate
ACS	Abbott Cardiovascular Systems
AE	Adverse Event
AMI	Acute Myocardial Infarction
BMS	Bare Metal Stent
CABG	Coronary Artery Bypass Graft
CBC	Complete Blood Count
CCS	Canadian Cardiovascular Society
CEC	Clinical Events Committee
CIP	Clinical Investigation Plan
CI-TLR	Clinically-Indicated Target Lesion Revascularization
CI-TVR	Clinically-Indicated Target Vessel Revascularization
CK	Creatine Kinase
CK-MB	Creatine Kinase Myocardial-Band Isoenzyme
cm	Centimeter
CRA	Clinical Research Associate
CRF	Case Report Form
CsA	Cyclosporin
CSS	Coronary Stent System
CVA	Cerebrovascular Accident or Stroke
DES	Drug Eluting Stent
dL	Deciliter
DLPLA	poly (D-lactide)
DS	Diameter Stenosis
DSMB	Data Safety Monitoring Board
EC	Ethics Committee
ECG	Electrocardiogram
eCRF	Electronic Case Resport Form
FDA	Food and Drug Administration
F/U	Follow-up
GCP	Good Clinical Practice
GI	Gastrointestinal
HbA_{1C}	Glycosylated Hemoglobin
hs-CRP	High-sensitivity C-reactive protein (hs-CRP)
IFU	Instructions for Use
IRB	Institutional Review Board
IVUS	Intravascular Ultrasound
LAD	Left Anterior Descending Coronary Artery
LCX	Left Circumflex Coronary Artery
LL	Late Loss

Acronym/ Abbreviation	Term
LVEF	Left Ventricular Ejection Fraction
MACE	Ischemia-Driven Major Adverse Cardiac Events
MEC	Medical Ethics Committee
μg	Microgram
mg	Milligram
MI	Myocardial Infarction
ML	MULTI-LINK
mL	Milliliter
MLD	Minimum Luminal Diameter
mm	Millimeter
NHLBI	National Heart, Lung, and Blood Institute
OUS	Outside of United States
PCI	Percutaneous Coronary Intervention
PLLA	poly (L-lactide)
PTCA	Percutaneous Transluminal Coronary Angioplasty
PLA	Poly Lactic Acid
QCA	Quantitative Coronary Angiography
RC	Research Coordinator
RCA	Right Coronary Artery
RCT	Randomized Clinical Trial
RX	Rapid eXchange
RVD	Reference Vessel Diameter
SAE	Serious Adverse Event
SAP	Statistical Analysis Plan
TIA	Transient Ischemic Attack
TIMI	Thrombolysis in Myocardial Infarction
TLR	Target Lesion Revascularization
TVF	Target Vessel Failure
TVR	Target Vessel Revascularization
UK	United Kingdom
US	United States
VO	Volume Obstruction
WBC	White Blood Cell Count

18 Appendix IV Study Flow / Follow-up Schedule

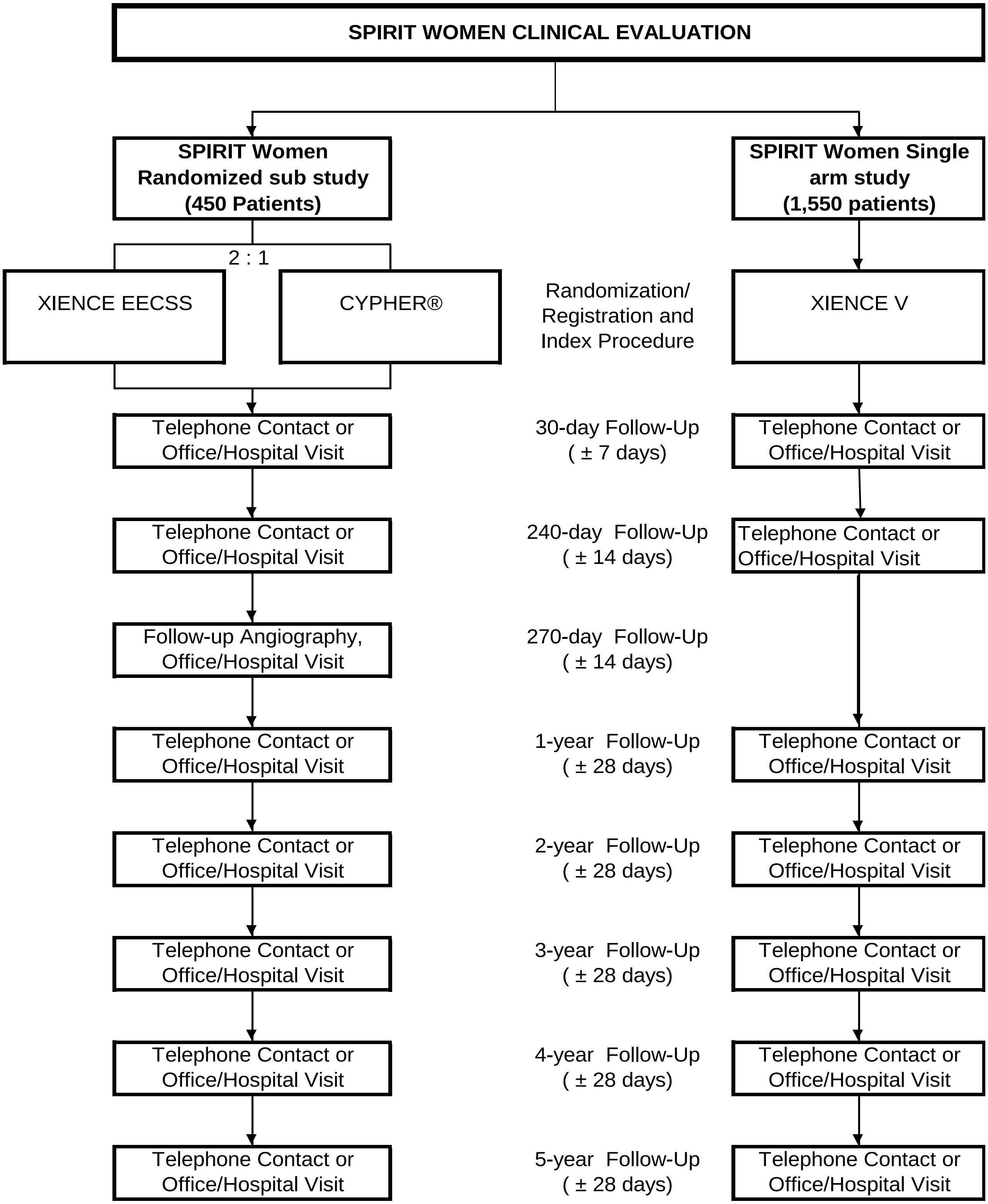

19 Appendix V Schedule of Events

PROCEDURE/TEST	Prior to Procedure	Pre-Procedure	Procedure	Post-Procedure	30 days (± 7 days) Hospital visit/Telephone call	240 days (± 14 days) Hospital visit/Telephone call	270 days (± 14 days) Hospital visit/	1 year (± 28 days) Hospital visit/Telephone call	2 year (± 28 days) Hospital Visit/Telephone Call	3 years (± 28 days) Hospital Visit/Telephone call	4 years (± 28 days) Hospital Visit/Telephone call	5 years (± 28 days) Hospital Visit/Telephone call	Additional visits
Patient Medical History	X												
Patient Informed Consent*	X												
Patient eligibility according to IFU	X												
Baseline Laboratory Work	X												
CK-MB/Troponin I/T	X[1]			X[2, 3]									
Serum Creatinine				X									
Patient demographics/characteristics		X											
ECG		X		X									
Coronary angiogram			X				X[4]						
PTCA/stent			X										
Per-CIP medications		X	X	X	X	X	X[4]	X	X	X	X	X	X
Anti-platelet therapy compliance					X	X	X[4]	X	X	X	X	X	X
Concomitant medications		X	X	X	X	X	X[4]	X	X	X	X	X	X
Adverse events			X	X	X	X	X[4]	X	X	X	X	X	X

1 = Cardiac enzymes, CKBM or Troponin I/T should be taken within 7 days prior to the index procedure.
2 = Single Arm Study: one sample should be taken in the post-procedure hospitalization period, but no later than 24 hours after the procedure.
3 = Randomized Sub-Study: First sample in the post-procedure hospitalization period. A second sample within 3 to 6 hours after the first post-procedure sample, but no later than 24 hours after the procedure.
* Written informed consent must be obtained before the pre-procedure angiography and prior to any study-related procedure.
4 = Only for patients enrolled in randomized sub-study

Patient demographics and characteristics will include the following assessments:

- Smoking history
- Diabetic status
- Menopausal status/years since menopause
- Hysterectomy and oophorectomy status
- Use of weak estrogens (black cohosh, soy, red clover)
- Past and current use of cardiac medication/statins
- Past and current use of hormonal contraceptives, hormonal replacement therapy
- Educational/professional status
- Body surface area
- Symptoms at presentation and referral path

20 Appendix VI Screening Process

Randomized Sub-study

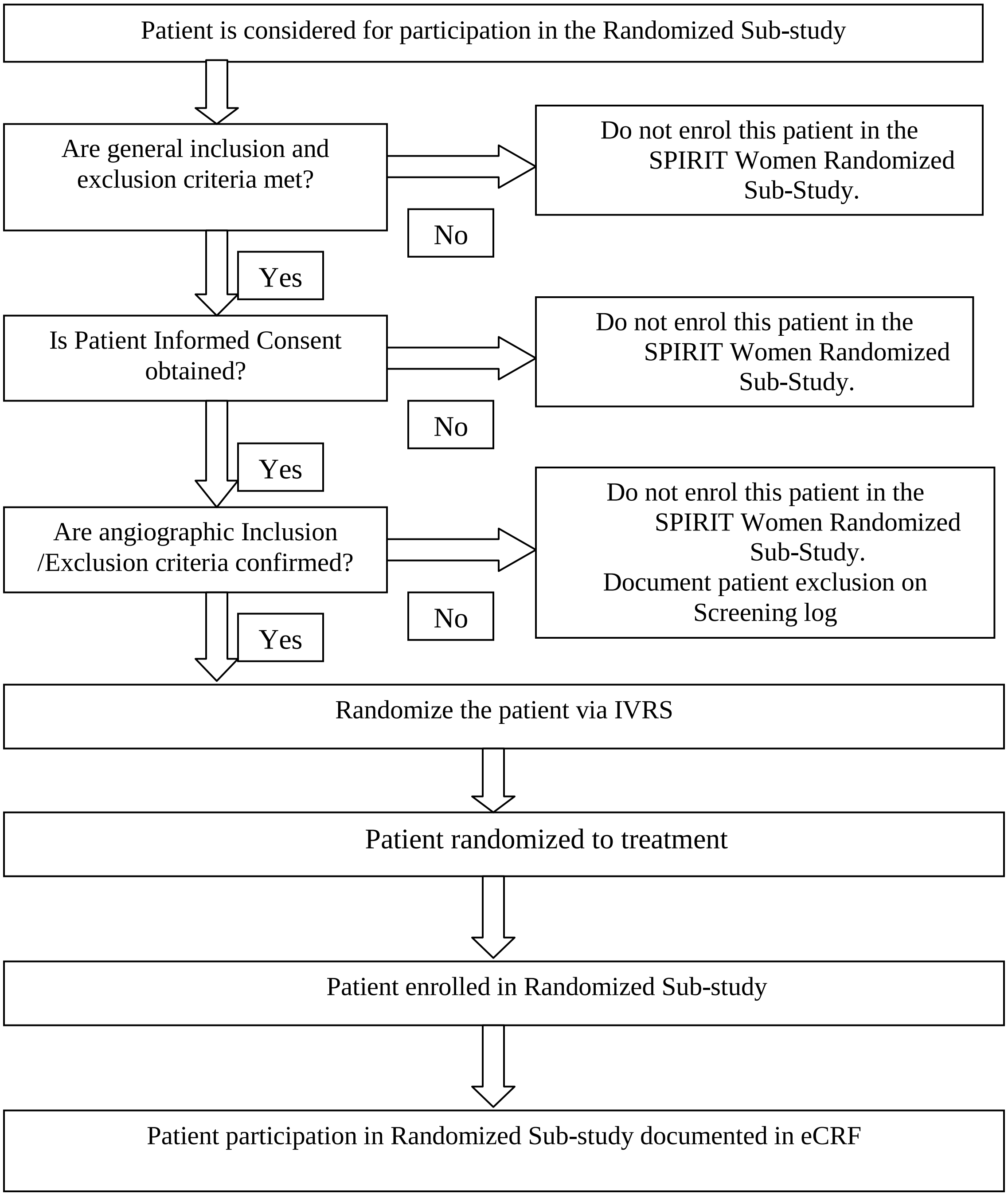

SPIRIT WOMEN Single Arm Study

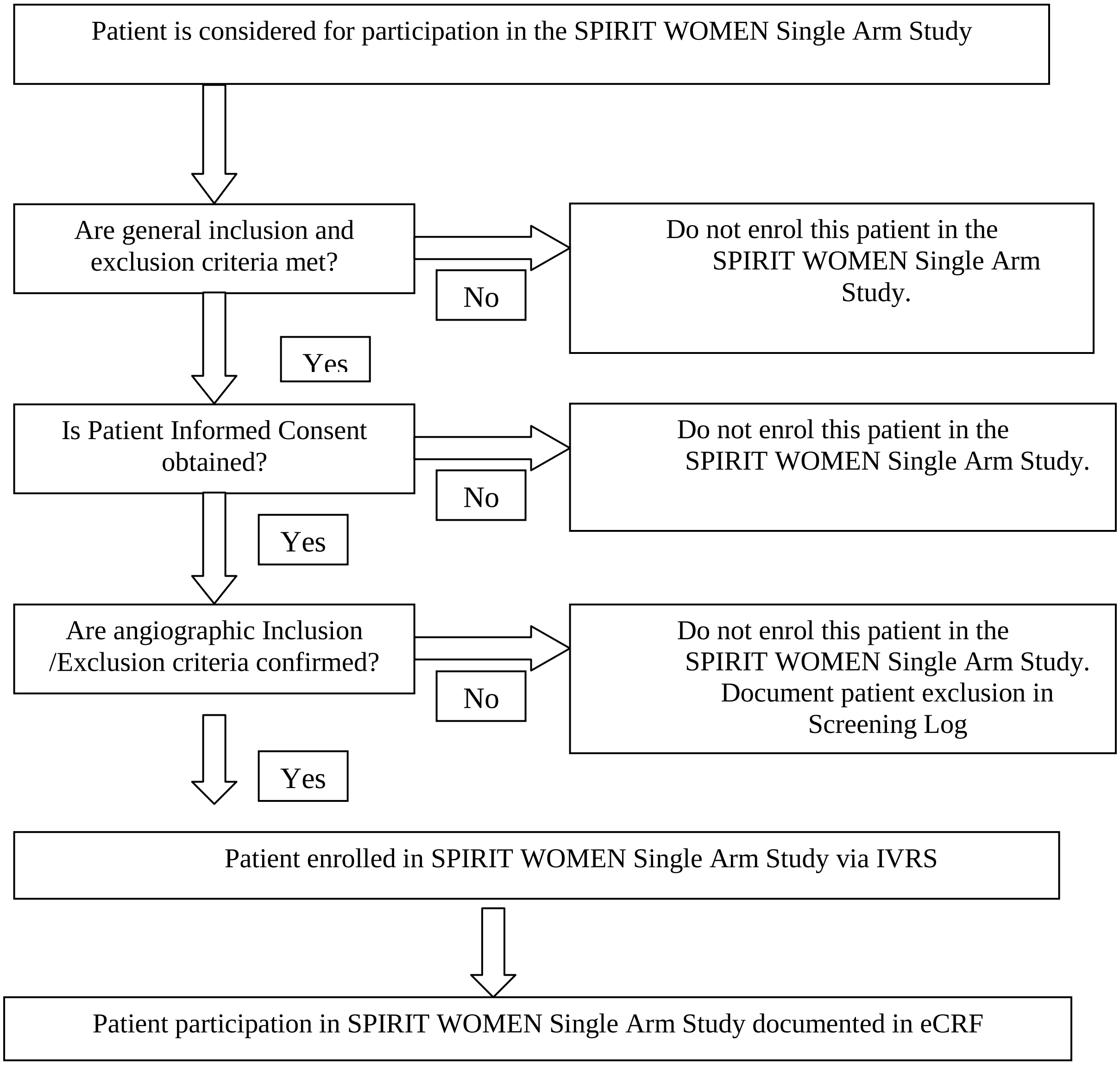

21 Appendix VII Clinical Investigation Contacts

**Principal Investigator:**	Dr. Marie-Claude Morice
**Co-Principal Investigator:**	Prof. Stephan Windecker
**Clinical Management:**	**Abbott Vascular International BVBA**<br>Park Lane<br>2B Culliganlaan<br>1831 Diegem (Belgium)<br>BE 0886.537.933<br><br>Susan Veldhof<br>Senior Manager Clinical Science<br>Mobile: +31 6534 28610<br>Email: susan.veldhof@av.abbott.com<br><br>Bridget Hurley<br>Director, Clinical Operations<br>Abbott Vascular International BVBA<br>Park Lane - Culliganlaan 2B<br>1831 Diegem, Belgium<br>Tel: +32 2 416 9223<br><br>Fax:+ 32 2 416 9101<br>Email: bridget.hurley@av.abbott.com
**List of Investigational Sites**	A list of site coordinates can be obtained upon request from the Clinical Project Manager

22 Appendix VIII REVISION HISTORY SPIRIT Women Protocol

V 1.0 Dated 19 March 2007 to V 2.0 Dated 19 August 2008
V 2.0 Dated 19 August 2008 to V 3.0 Dated 10 December 2008
V 3.0 Dated 10 December 2009 to V 4.0 Dated 25 August 2009

Section/Pg in Version 1.0	Version 1.0	Version 2.0
Throughout document	SPIRIT Women	XIENCE V SPIRIT WOMEN
Throughout document		General formatting
Throughout document		Add 'approximately' to all patient numbers
Front Cover		Add: **Study Monitoring**<br>Quintiles Limited,<br>Station House, Market Street, Bracknell, Berkshire<br>RG12 1HX United Kingdom
Front Cover,	**CONTACT:** Koen Mol<br>Clinical Project Manager<br>Abbott Vascular International BVBA<br>International Clinical Operations<br>Park Lane<br>Culliganlaan 2B<br>1831 Diegem<br>Belgium<br>Tel: +32-2-41-69992<br>Fax: +32-2-71-41615	**CONTACT:** Bridget Hurley<br>Director, International Clinical Operations<br>Abbott Vascular International BVBA<br>Park Lane - Culliganlaan 2B<br>1831 Diegem, Belgium<br>Tel: +32 2 416 9223<br>Fax:+ 32 2 416 9101
Summary<br>2.1.1, p. 11<br>2.2, p. 11<br>4.0, p. 14	The XIENCE V EECSS has received CE mark approval on 30 January 2006.	The XIENCE V EECSS has received CE mark approval on 30 January 2006 and FDA approval on 02 July 2008.
Summary<br><br>Treatment Strategy p. 7	**All Patients:**<br>Patients may receive up to 4 planned study stents depending on the number of vessels treated and their respective lesion lengths<br>• Treatment of target lesion(s) measuring ≤ 28 mm in length by visual estimation<br>**Note:** Overlap of a minimum of 1 mm and a maximum of 4 mm<br>• Pre-dilatation of target lesion(s) is mandatory.	**All Patients:**<br>The treatment strategy will be determined by the Investigator. It is recommended that each enrolling Investigator review the most recent IFU to assess contraindications, warnings, and precautions for treating potential patients.<br><br>No non-study percutaneous procedures can be performed at the time of the study procedure

Section/Pg in Version 1.0	Version 1.0	Version 2.0
	Recommendation to use a pre-dilatation catheter that is 0.5 mm smaller than the reference vessel<br>• Pre-dilated area needs to be covered completely with the stent(s) such that a minimum of 3 mm of healthy vessel on either side of the lesion is covered by the stent<br>• It is highly recommended not to perform atherectomy in conjunction with this procedure. If it is used, the area treated with atherectomy should be restricted to the lesion area and be completely covered by the study stent<br>• Brachytherapy in any lesion or vessel must not be performed in conjunction with this procedure<br>• Rated burst pressure as indicated in the product labeling of the study stent should not be exceeded<br>• Post-dilatation is left to the discretion of the investigator, however if performed, should only be done with balloons sized to fit within the boundaries of the stent<br>• No non-study percutaneous procedures can be performed at the time of the study procedure<br>**SPIRIT Women Single Arm Study:**<br>After meeting the general and angiographic inclusion and exclusion criteria, the target lesion(s) should be treated by stenting with the XIENCE V EECSS according to the IFU<br>Registration should only be done after confirmation of the angiographic inclusion criteria and before implantation of first stent<br>All lesions should be planned to be treated with the XIENCE V	**SPIRIT Women Single Arm Study:**<br>After meeting the general and angiographic inclusion and exclusion criteria, the target lesion(s) should be treated by stenting with the XIENCE V EECSS according to the IFU<br>Registration should only be done after confirmation of the angiographic inclusion criteria and before implantation of first stent<br>All lesions should be planned to be treated with the XIENCE V<br><br>If any staged procedures are planned at baseline, it is necessary that each investigator treats the enrolled patients with only XIENCE V EECSS during the planned staged procedure.<br>If bailout stenting is necessary, a XIENCE V EECSS of appropriate length should be used<br>It is recommended that treatment with clopidogrel should be continued at 75 mg daily for a period of at least 6 months. Ticlopidine at a dose standard with hospital practice may be substituted for clopidogrel, as required by hospital practice or if the patient develops hypersensitivity to clopidogrel.<br>It is recommended that all patients should receive ≥ 75 mg of aspirin daily for a minimum of five years following the procedure<br><br>**Randomized Sub-study:**<br>After meeting the general and angiographic inclusion and exclusion criteria, the target lesion(s) should be treated

Section/Pg in Version 1.0	Version 1.0	Version 2.0
	If bailout stenting is necessary, a XIENCE V EECSS of appropriate length should be used<br><br>It is recommended that treatment with clopidogrel should be continued at 75 mg daily for a period of at least 6 months. If a patient develops hypersensitivity to clopidogrel, they may be switched to ticlopidine at a dose in accordance with standard hospital practice<br><br>It is recommended that all patients should receive ≥ 75 mg of aspirin daily for a minimum of five years following the procedure<br><br>**Randomized Sub-study:**<br><br>After meeting the general and angiographic inclusion and exclusion criteria, the target lesion(s) should be treated according to the same randomization schedule. If two or more target lesions are to be treated, all lesions must receive the treatment that has been assigned as per the randomization<br><br>Randomization should be done only after confirmation of the angiographic inclusion criteria and before implantation of the first stent. Once randomization is completed and a treatment is assigned, crossover is not permitted.<br><br>If bailout stenting is necessary, the stent used should be the same as allocated by randomization for the index procedure, and should be used according to the Instructions for Use (IFU)<br><br>All patients will receive clopidogrel at 75 mg daily for a period of at least 6 months. If a patient develops hypersensitivity to clopidogrel, they may be switched to ticlopidine at a dose in accordance with standard hospital practice.	according to the same randomization schedule. If two or more target lesions are to be treated, all lesions must receive the treatment that has been assigned as per the randomization<br><br>Randomization should be done only after confirmation of the angiographic inclusion criteria and before implantation of the first stent. Once randomization is completed and a treatment is assigned, crossover is not permitted.<br><br>If bailout stenting is necessary, the stent used should be the same as allocated by randomization for the index procedure, and should be used according to the Instructions for Use (IFU)<br><br>If any staged procedures are planned at baseline, it is necessary that each investigator treats the enrolled patients with the same study stent during the planned staged procedure as was used at baseline.All patients will receive clopidogrel at 75 mg daily for a period of at least 6 months. Ticlopidine at a dose standard with hospital practice may be substituted for clopidogrel, as required by hospital practice or if the patient develops hypersensitivity to clopidogrel.<br><br>All patients will receive ≥ 75 mg of aspirin daily for a minimum of five years following the procedure

Section/Pg in Version 1.0	Version 1.0	Version 2.0
	All patients will receive ≥ 75 mg of aspirin daily for a minimum of five years following the procedure	
2.1.1, p. 10	The SPIRIT II clinical trial includes clinical follow up at 30, 180, 270 days and 1- 2 years.	The SPIRIT II clinical trial includes clinical follow up at 30, 180, 270 days and 1- 5 years.
2.1.1, p. 11	The SPIRIT III trial is a pivotal clinical trial conducted in the United States (US) and Japan. It consists of five parts, a US randomized clinical trial (RCT) (N=1002), a 2.25 mm non randomized arm (N=105), a 4.0mm non randomized arm (N=80), a 38 mm non randomized arm (N=105), and one non-randomized arm in Japan (N=88),…..	SPIRIT III, a pivotal clinical trial conducted in the USA and Japan, was designed to compare XIENCE V EECSS performance to the TAXUS PECSS. The SPIRIT III USA randomized clinical trial indicates that XIENCE V EECSS efficacy is statistically non-inferior to the TAXUS PECSS in single and multiple de novo native coronary artery lesions.[18] Moreover, the primary endpoint was met with an in-segment late loss of 0.14 ± 0.41 mm for the XIENCE V EECSS arm and 0.28 ± 0.48 mm for the TAXUS PECSS arm at 240 days (non-inferiority $p < 0.0001$, delta = 0.195 mm).
2.2, p. 11		Add picture of XV Stent 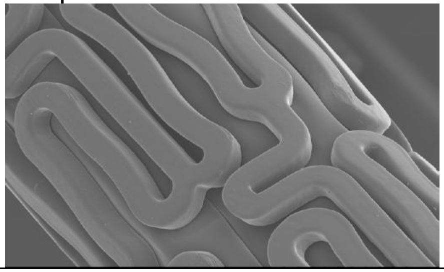
Section 4.2<br>Treatment Strategy p. 14-15	**The treatment strategy for all patients enrolled in the clinical evaluation is as follows:**<br>• Loading dose of clopidogrel of at least 300 mg prior to the start of the procedure. This dose may be adjusted if the patient is already taking clopidogrel. Recommended between 12 and 6 hours before procedure, however no later than 1 hour post procedure.	**The treatment strategy for all patients enrolled in the clinical evaluation is as follows:**<br>The treatment strategy will be determined by the investigator. It is recommended that each enrolling investigator review the most recent IFU to assess contraindications, warnings, and precautions for treating potential patients.

Section/Pg in Version 1.0	Version 1.0	Version 2.0
	• Treatment of *de novo* native coronary artery lesions with up to 4 planned study stents depending on the number of vessels treated and their respective lesion lengths.<br>• Treatment of target lesion(s) measuring ≤ 28 mm in length by visual estimation.<br>**Note**: Overlap of a minimum of 1 mm and a maximum of 4 mm<br>• Pre-dilatation of target lesion(s) is mandatory. Recommendation to use a pre-dilatation catheter that is 0.5 mm smaller than the reference vessel.<br>• Pre-dilated area needs to be covered completely with the stent(s) such that a minimum of 3 mm of healthy vessel on either side of the lesion is covered by the stent.<br>• Brachytherapy in any lesion or vessel must not be performed in conjunction with this procedure.<br>• Rated burst pressure as indicated in the product labeling of the study stent should not be exceeded.<br>• Post-dilatation is left to the discretion of the investigator, however if performed, should only be done with balloons sized to fit within the boundaries of the stent.<br>• No non-study percutaneous procedures can be performed at the time of the study procedure<br>**Treatment strategy for the CYPHER SELECT™ Plus Sirolimus-eluting Coronary Stent**<br>Use of the CYPHER SELECT™ Plus Sirolimus-eluting Coronary Stent should be according to its Instructions for Use.<br>**Additional treatment strategy for patients enrolled in the SPIRIT Women Single Arm Study is as follows:**	Loading dose of clopidogrel of at least 300 mg prior to the start of the procedure. This dose may be adjusted if the patient is already taking clopidogrel. Recommended prior to the start of the procedure, however no later than 1 hour post procedure.<br>No non-study percutaneous procedures can be performed at the time of the study procedure<br>**Additional treatment strategy for patients enrolled in the SPIRIT Women Single Arm Study is as follows:**<br>After meeting the general and angiographic inclusion and exclusion criteria, the target lesions should be treated by stenting with the XIENCE V EECSS according to the IFU.<br>Registration should only be done after confirmation of the angiographic inclusion criteria and before implantation of the first stent.<br>All lesions should be planned to be treated with the XIENCE V.<br>If any staged procedures are planned at baseline, it is necessary that each investigator treats the enrolled patients with only XIENCE V EECSS during the planned staged procedure.<br>Bailout stenting with XIENCE V EECSS of appropriate length.<br>It is recommended that all patients should receive ≥ 75 mg of aspirin daily for a minimum of five years following the procedure.<br>It is recommended that treatment with clopidogrel should be continued at 75 mg daily for a period of at least 6 months.

Section/Pg in Version 1.0	Version 1.0	Version 2.0
	After meeting the general and angiographic inclusion and exclusion criteria, the target lesions should be treated by stenting with the XIENCE V EECSS according to the IFU.<br><br>Registration should only be done after confirmation of the angiographic inclusion criteria and before implantation of the first stent.<br><br>All lesions should be planned to be treated with the XIENCE V.<br><br>Bailout stenting with XIENCE V EECSS of appropriate length.<br><br>It is recommended that all patients should receive ≥ 75 mg of aspirin daily for a minimum of five years following the procedure.<br><br>It is recommended that treatment with clopidogrel should be continued at 75 mg daily for a period of at least 6 months. If a patient develops hypersensitivity to clopidogrel, they may be switched to ticlopidine at a dose in accordance with standard hospital practice.<br><br>**Additional treatment strategy for patients enrolled in the randomized sub-study is as follows:**<br>After meeting the general and angiographic inclusion and exclusion criteria, the target lesion(s) should be treated according to the same randomization schedule. If two or more target lesions are to be treated, all lesions must receive the treatment that has been assigned as per the randomization.<br><br>Randomization should be done only after confirmation of the angiographic inclusion criteria and before implantation of the first stent. Once randomization is completed and a treatment is	Ticlopidine at a dose standard with hospital practice may be substituted for clopidogrel, as required by hospital practice or if the patient develops hypersensitivity to clopidogrel.<br><br>**Additional treatment strategy for patients enrolled in the randomized sub-study is as follows:**<br>After meeting the general and angiographic inclusion and exclusion criteria, the target lesion(s) should be treated according to the same randomization schedule. If two or more target lesions are to be treated, all lesions must receive the treatment that has been assigned as per the randomization.<br><br>Randomization should be done only after confirmation of the angiographic inclusion criteria and before implantation of the first stent. Once randomization is completed and a treatment is assigned, crossover is not permitted.<br><br>If it is not possible to implant the stent allocated by randomization, it is recommended that any other commercially available stent, either BMS or DES be implanted, rather than the XIENCE V or the CYPHER SELECT™ Plus Sirolimus-eluting Coronary Stent.<br><br>If bailout stenting is necessary, the stent used should be the same as allocated by randomization for the index procedure, and should be used according to the Instructions for Use (IFU).<br><br>If any staged procedures are planned at baseline, it is necessary that each investigator treats the enrolled patients with the same study stent during the planned staged procedure as was used at baseline.<br>All patients should receive ≥ 75 mg of aspirin daily for a

Section/Pg in Version 1.0	Version 1.0	Version 2.0
	assigned, crossover is not permitted.<br><br>If it is not possible to implant the stent allocated by randomization, it is recommended that any other commercially available stent, either BMS or DES be implanted, rather than the XIENCE V or the CYPHER SELECT™ Plus Sirolimus-eluting Coronary Stent.<br><br>If bailout stenting is necessary, the stent used should be the same as allocated by randomization for the index procedure, and should be used according to the Instructions for Use (IFU).<br><br>All patients should receive ≥ 75 mg of aspirin daily for a minimum of five years following the procedure.<br><br>All patients should receive clopidogrel 75 mg daily for a period of at least 6 months. If a patient develops hypersensitivity to clopidogrel, they may be switched to ticlopidine at a dose in accordance with standard hospital practice.	minimum of five years following the procedure.<br><br>All patients should receive clopidogrel 75 mg daily for a period of at least 6 months Ticlopidine at a dose standard with hospital practice may be substituted for clopidogrel, as required by hospital practice or if the patient develops hypersensitivity to clopidogrel.<br><br>**Treatment strategy for the CYPHER SELECT™ Plus Sirolimus-eluting Coronary Stent**<br>Use of the CYPHER SELECT™ Plus Sirolimus-eluting Coronary Stent should be according to its Instructions for Use.
4.3 p. 15	Once randomized/registered the patient is considered enrolled in the study and part of the intent-to-treat population.	Once randomized/registered the patient is considered enrolled in the study.
6.2.1, p. 17		**Add**: The obtaining of the consent, provision of a copy to the subject, along with the date and time should be documented in the subject's medical records and signed by the investigator.
6.2.2, p. 17	…BS EN ISO 14155-1:2003 and BS EN ISO 14155-2:2003 standards and ICH/GCP guidelines where relevant and appropriate and provided these do not oppose the applicable law.	…BS EN ISO 14155-1:2003 and BS EN ISO 14155-2:2003 standards provided these do not oppose the applicable law.
6.5, p. 19	If these attempts are unsuccessful, a certified letter should be	If these attempts are unsuccessful, a certified letter should

Section/Pg in Version 1.0	Version 1.0	Version 2.0
	sent to the patient and/or the patient's General Practitioner/Family Doctor.	be sent to the patient and/or the patient's General Practitioner/Family Doctor (this should be done only when the patient has consented to direct contact with General Practitioner/Family Doctor)
7.1.1, p.20	Past and present use of hormonal contraceptives/hormone replacement therapy (including mode of administration), smoking history, educational/professional status, body surface area and chronic concomitant medications	Past and present use of hormonal contraceptives/hormone replacement therapy (including mode of administration), smoking history, educational/professional/social status such as number of children, cohabitation, employment and educational degree,
7.1.2 p. 20	For Local lab<br><br>For Central lab<br><br>Note:* If CK is greater than the upper limit of normal, CK-MB or Troponin must be done.<br>** Troponin may be assessed if this is normal hospital practice. If the Troponin is elevated, CK or CK-MB must be assessed.	Delete requirement for CK and Creatinine pre procedure.<br><br>Indicate that CKMB or Troponin I/T must be taken<br><br>Delete CK and add Creatinine<br><br>Deleted
7.1.3, p. 21	The following parameters should be measured within 7 days prior to the index procedure:	The following parameters must be measured within 7 days prior to the index procedure:
7.1.4 Medications p. 21	Patients selected for coronary stenting should receive a loading dose of antiplatelet medications (unless patient is under chronic antiplatelet medications) prior to the start of the procedure. Medications administered will include loading dose of clopidogrel bisulfate (≥ 300 mg) and aspirin (≥ 75 mg). Ticlopidine hydrochloride at a dose according to standard hospital practice may be given if patient is allergic to clopidogrel bisulfate. If patient is already receiving clopidogrel bisulfate (or ticlopidine hydrochloride), loading dose can be	Patients selected for coronary stenting should receive a loading dose of antiplatelet medications (unless patient is under chronic antiplatelet medications) prior to the start of the procedure, but no later than one hour post procedure. Medications administered will include loading dose of clopidogrel bisulfate (≥ 300 mg) and aspirin (≥ 75 mg). Ticlopidine at a dose standard with hospital practice may be substituted for clopidogrel, as required by hospital practice or if the patient develops hypersensitivity to clopidogrel. If

Section/Pg in Version 1.0	Version 1.0	Version 2.0
	adjusted per physician's discretion, but not omitted.	patient is already receiving clopidogrel bisulfate (or ticlopidine hydrochloride), loading dose can be adjusted per physician's discretion, but not omitted.
7.2, p. 21	Written informed consent can be obtained before or after the pre-procedure angiography.	After meeting the general inclusion and exclusion criteria, written informed consent can be obtained before or after the pre-procedure angiography.
7.3.3.1, p. 22	Once the general and angiographic inclusion and exclusion criteria have been confirmed, the patient should be registered via the IVRS registration system before the placement of the first stent.<br>All target lesion(s) must be pre-dilated.<br>Treatment of target lesion(s) should be with either a single stent or planned overlapping stents.<br>Prior to use, the XIENCE V EECSS will be inspected and prepared according to the Instructions for Use (IFU). All XIENCE V EECSS will be delivered and deployed per the IFU.<br><br>All the target lesion(s) should be treated by stenting with the XIENCE V EECSS.<br>Patients may receive up to 4 planned XIENCE V EECSS stents depending on the number of vessels treated and their respective lesion lengths.	Once the general and angiographic inclusion and exclusion criteria have been confirmed, the patient should be registered via the IVRS registration system before the placement of the first stent.<br>Treatment of target lesion(s) should be with either a single stent or planned overlapping stents.<br>Prior to use, the XIENCE V EECSS should be inspected and prepared according to the Instructions for Use (IFU). All XIENCE V EECSS should be delivered and deployed per the IFU.<br><br>All the target lesion(s) should be treated by stenting with the XIENCE V EECSS.
7.3.3.2, p. 22	Baseline angiography of the target vessel will be completed as per the Angiographic Core Laboratory Guidelines.<br>Once the general and angiographic inclusion and exclusion criteria have been confirmed, the patient should be randomized via IVRS randomization service prior to the placement of the first stent.<br>All target lesion(s) must be pre-dilated.<br>Prior to use, the XIENCE V EECSS will be inspected and prepared according to the Instructions for Use (IFU). All XIENCE V EECSS will be delivered and deployed per the IFU.	Baseline angiography of the target vessel will be completed as per the Angiographic Core Laboratory Guidelines.<br>Once the general and angiographic inclusion and exclusion criteria have been confirmed, the patient should be randomized via IVRS randomization service prior to the placement of the first stent.<br>Prior to use, the XIENCE V EECSS should be inspected and prepared according to the Instructions for Use (IFU). All XIENCE V EECSS should be delivered and deployed per the IFU.

Section/Pg in Version 1.0	Version 1.0	Version 2.0
	All CYPHER SELECT™ Plus Sirolimus-eluting Coronary Stent use should be according to its IFU.<br>All target lesion(s) should be treated by stenting with the stent assigned per randomization, either XIENCE V EECSS or CYPHER SELECT™ Plus Sirolimus-eluting Coronary Stent.<br>Treatment of target lesion(s) should be with either a single stent or planned overlapping stents.<br>Patients may receive up to 4 planned stents depending on the number of vessels treated and their respective lesion lengths.	All CYPHER SELECT™ Plus Sirolimus-eluting Coronary Stent use should be according to its IFU.<br>All target lesion(s) should be treated by stenting with the stent assigned per randomization, either XIENCE V EECSS or CYPHER SELECT™ Plus Sirolimus-eluting Coronary Stent.<br>Treatment of target lesion(s) should be with either a single stent or planned overlapping stents.
7.3.4, p. 22	In order to eliminate or mitigate peri-stent effects, special care needs to be taken to minimize injury to areas adjacent to the stent boundaries. To minimize injury to unstented areas, the following guidelines need to be followed:<br>Pre-dilatation of the target lesion must be performed prior to stent deployment with an angioplasty balloon. It is recommended to use a pre-dilatation catheter that is 0.5 mm smaller in diameter than the reference vessel<br>Limit pre-dilatation injury within the area to be stented. The balloon used for pre-dilatation must be shorter than the length of the stent(s)<br>Pre-dilated area needs to be covered completely with the stent such that a minimum of 3 mm of healthy vessel on either side of the lesion is covered by the stent<br>It is highly recommended not to perform atherectomy or cutting balloon in conjunction with this procedure. If it is used, the area treated with atherectomy or cutting balloon should be restricted to the lesion area and be completely covered by the study stent<br>Do not exceed the rated burst pressure as indicated in the	The treatment strategy will be determined by the investigator. It is recommended that each enrolling investigator review the most recent XIENCE V IFU to assess contraindications, warnings, and precautions for treating potential patients.

Section/Pg in Version 1.0	Version 1.0	Version 2.0
	product labeling.<br><br>Post-dilatations may or may not be performed at operator discretion, but when performed must only be done with balloons sized to fit within the boundaries of the stent<br><br>Brachytherapy in any lesion or vessel must not be performed in conjunction with this procedure.<br><br>Overlapping stents should be placed with a minimum of 1 mm and a maximum of 4 mm overlap.<br><br>In case of planned overlapping of stents, the distal stent should be deployed first, followed by deployment of proximal stent. Stenting in this order alleviates the need to cross the proximal stent in placement of the distal stent and reduces the chances for dislodging the proximal stent.	
7.3.6, p. 23	**IMPORTANT:** For the XIENCE V it is recommended that bailout stent should be placed so that there is no visible gap between the stents. In such a case, at least 1 mm (minimum) to 4 mm (maximum) overlap is required.	**IMPORTANT:** For the XIENCE V it is recommended that bailout stent should be placed so that there is no visible gap between the stents. In such a case, at least 1 mm (minimum) to 4 mm (maximum) overlap is recommended.
7.4 Post procedure, p. 24	Following the procedure, all patients will be treated in accordance with the hospital standard of care. An ECG will be taken between 12 hours following the index procedure and hospital discharge.<br><br>Routine hospital blood-work (including CK, CK-MB and/or Troponin and Creatinine) should be taken per hospital standards for patient management*.<br><br>Samples for CK* (and CK-MB or Troponin, if CK is elevated) for local (all patients) and central laboratory measurements (for patients enrolled in the randomized sub-study) must be taken between 8 and 24 hours post-procedure at least twice,	**Post-Procedural Testing and Medications**<br>Following the procedure, all patients will be treated in accordance with the hospital standard of care.<br><br>**ECG**<br>An ECG must be taken within the post-procedure hospitalization period for all patients.<br><br>***Cardiac Enzymes and Creatinine***<br><br>Routine hospital blood-work and testing should be taken/performed per hospital standards for patient management and must include at least the following:

Section/Pg in Version 1.0	Version 1.0	Version 2.0
	with the second sample taken at least 4 hours apart from the first sample. This should be repeated more often in case of serial measurements per routine hospital blood work due to elevation.<br><br>*If CK is greater than the upper limit of normal, CK-MB or Troponin must be done. Troponin may be assessed if this is normal hospital practice. If the Troponin is elevated, CK or CKMB must be assessed.	*XIENCE V SPIRIT WOMEN Single Arm Study (Local Laboratory)*<br>One sample for CKMB or Troponin I/T must be taken in the post-procedural hospitalization period, but no later than 24 hours after the procedure.<br><br>*Randomized Sub-study (Central Laboratory)*<br>One sample for CKMB or Troponin I/T must be taken in the post-procedural hospitalization period. A second sample must be taken within 3 to 6 hours after the first post-procedure sample, but no later than 24 hours after the procedure.<br><br>One sample for Creatinine must be taken in the post-procedural hospitalization period.
7.4.1.1<br>7.4.1.2, p. 24	If a patient develops sensitivity to clopidogrel bisulfate, they may be switched to ticlopidine hydrochloride at a dose according to standard hospital practice.	Ticlopidine at a dose standard with hospital practice may be substituted for clopidogrel, as required by hospital practice or if the patient develops hypersensitivity to clopidogrel.
8.1, p. 25	Data regarding adverse events including related laboratory tests, medications and/or ECGs.	Data regarding adverse events including related laboratory tests, medications and/or ECGs. Following the 2 year follow-up it is only required to record and collect information on adverse cardiac events and serious adverse events.
8.3.2, p. 28	Starting with the device implant, any new event/experience that was not present at baseline, or worsening of an event present at baseline, is considered an adverse event.<br><br>An adverse event may be expected. Expected AEs may include:	Beginning with the start of the index procedure, which is defined as the time of the arterial puncture, any new event/experience that was not present at baseline, or worsening of an event present at baseline, is considered an adverse event and should be reported as such.<br><br>An adverse event may be expected (however should still be reported through 2 year follow-up). Expected AEs may include:

**Section/Pg in Version 1.0**	**Version 1.0**	**Version 2.0**
8.3.2, p. 28	Definitions for determination of the relationship include:<br><br>**Not Related:** Exposure to the investigational product has not occurred, or the occurrence of the adverse event is not reasonably related in time, or the adverse event is considered unlikely to be related to the use of the investigational product (biologically implausible).<br><br>**Related:** Exposure to the investigational product and the adverse event are reasonably related in time and the investigational product is more likely than other causes to be responsible for the adverse event, or is the most likely cause of the adverse event.<br><br>**Unknown:** Exposure to the device and the occurrence of adverse event cannot be reasonably determined to be unrelated to the device. If the relationship is identified as unknown it will be treated as related to the investigational product.<br><br>If any adverse event is considered related to the administration of the investigational product, that event will be followed until resolution or the Investigator judges the event to be chronic or stable.	Definitions for determination of the relationship include:<br><br>**Not Related:** Exposure to device has not occurred, or the occurrence of the adverse event is not reasonably related in time, or the adverse event is considered unlikely to be related to the use of the device (biologically implausible).<br><br>**Related:** Exposure to the device and the adverse event are reasonably related in time and the device is more likely than other causes to be responsible for the adverse event, or is the most likely cause of the adverse event.<br><br>**Unknown:** Exposure to the device and the occurrence of adverse event cannot be reasonably determined to be unrelated to the device. If the relationship is identified as unknown it will be treated as related to the device.<br><br>If any adverse event is considered related to the administration of the device, that event will be followed until resolution or the Investigator judges the event to be chronic or stable.
8.4, p. 29	**Serious/Severe Adverse Events**	**Serious Adverse Events**<br>Note: All device malfunctions should be reported to the Sponsor within 24 hours of knowledge.
8.5, p. 29		This section deleted as not applicable for non FDA studies Definition deleted from Appendix II.
8.7.1, p. 29	The Clinical Events Committee (CEC) is an independent adjudication body comprised of interventional and/or non-interventional cardiologists who are not participants in the clinical evaluation. The CEC will review and adjudicate all	The Clinical Events Committee (CEC) is an independent adjudication body comprised of interventional and/or non-interventional cardiologists who are not participants in the clinical evaluation.

Section/Pg in Version 1.0	Version 1.0	Version 2.0
	endpoint related and major bleeding and vascular events according to protocol definitions provided in Appendix II. These definitions are based on the final draft of the Dublin definitions intended for publication in Circulation xxx. Differences between the definitions as published and as found in Appendix II will be taken into account when adjudicating clinical events.	The CEC will review and adjudicate all endpoint related and major bleeding and vascular events according to the definitions provided in Appendix II. These definitions are based on the definitions developed by the Academic Research Consortium (ARC), as published in Circulation (Cutlip, D.E., et al., Clinical End Points in Coronary Stent Trials: A Case for Standardized Definitions. Circulation, 2007. 115: p. 2344-2351.)
9.2, p. 31		Added: A drop-out rate of 3% is expected at 1-year.
9.3.1, p. 31	The intent-to-treat population will consist of all patients (all target lesions) enrolled in the study. For the randomized sub-study, patients will be analyzed in the treatment group to which they were randomized.	The intent-to-treat population for the randomized sub-study will consist of all patients (all lesions) randomized, regardless of the treatment actually received. Subjects will be analyzed in the treatment arm to which they were randomized.<br><br>Analysis for the Single Arm Study will be performed on the intent-to-treat population which will include all patients<br>1. who have signed the Patient Informed Consent<br>2. who have been registered in the ICON IVRS system<br>AND<br>3. who comply to at least one of the following:<br>a. at least one XIENCE V stent has been implanted<br>b. a non-XIENCE V stent has been implanted after an intention to or an attempt made to implant a XIENCE V<br>c. no stent was implanted after an intention to or an attempt made to implant a XIENCE V<br><br>Patients not meeting the above criteria will not be required to undergo follow up as mandated by the CIP<br><br>Adverse event reported for patients who comply to criteria numbers 1 and 2 as mentioned above will be adjudicated but not included in the analysis even if criterion number 3 is not met.

Section/Pg in Version 1.0	Version 1.0	Version 2.0
		Lesions for which there was no attempt to implant a XIENCE V EECS and lesions located in a bypass graft will not be considered in the analysis for the Single Arm Study.
11.2.2, p. 34	The investigator will not deviate from the clinical investigation plan for any reason without prior written approval from Sponsor, except in cases of medical emergencies, when the deviation is necessary to eliminate an apparent immediate hazard to the patient. In that event, the investigator will notify Sponsor immediately by phone or in writing.	The investigator will not deviate from the clinical investigation plan for any reason without prior written approval from Sponsor, except in cases of medical emergencies, when the deviation is necessary to eliminate an apparent immediate hazard to the patient. In that event, the investigator will notify Sponsor and document the deviation in the electronic CRF.
11.2.3 p. 34		Added: Follow-up for all enrolled patients will be as per protocol requirements.
11.2.4, p. 34		Added: Product expiration dates should be checked prior to implantation**.**
11.3.1, p. 34	Training will include, but not be limited to, the investigational plan, investigational device usage, clinical investigation plan requirements, electronic case report form completion, and clinical investigation personnel responsibilities.	Training will include, but not be limited to, the investigational plan, investigational device usage, good clinical practices, electronic case report form completion, and clinical investigation personnel responsibilities.
13, p. 39	For the clinical evaluation duration, the investigator will maintain complete and accurate documentation including but not limited to medical records, clinical evaluation progress records, laboratory reports, case report forms, signed informed consent forms, correspondence with the MEC and clinical evaluation monitor/Sponsor, adverse event reports, and information regarding patient discontinuation or completion of the clinical evaluation.	For the clinical evaluation duration, the investigator will maintain complete and accurate documentation including but not limited to:<br><br>Correspondence with the sponsor, Ethics Committee and other investigators<br>The signed CIP with any and all amendments<br>Signed Informed Consent Forms<br>Ethics Committee approval of the CIP and any amendment<br>The investigators' agreement and the fee agreement (if applicable)<br>The investigator's brochure / Instructions For Use<br>The insurance certificate

Section/Pg in Version 1.0	Version 1.0	Version 2.0
		Curricula vitae for the investigator and all co-investigators<br>Laboratory Normal Reference Ranges<br>Laboratory Certificate (or equivalent documentation)<br>Complete and adequate source documents<br>Financial Disclosure forms of all treating physicians<br>Case Report Forms<br>Adverse Event Reports<br>Information regarding patient discontinuation or completion of the clinical evaluation.
13.1 p. 39	Dated and signed notes on the day of entry into the study including the study sponsor (ACS), CIP number, Clinical Site, patient number assigned and a statement that informed consent was obtained.<br><br>Notations on abnormal lab results	Dated and signed notes on the day of entry into the study including the study name (or abbreviation), treatment assigned and a statement that informed consent was obtained prior to any study-related procedure.<br><br>Study required or endpoint related lab reports and ECGs, signed and dated for review, and annotated for clinically significant of out of range results.
Appendix V	1 = If CK is greater than the upper limit of normal, CK-MB or Troponin must be done. Troponin may be assessed if this is normal hospital practice. If the Troponin is elevated, CK or CKMB must be assessed.<br>2 = Two times between 8 and 24hours post-procedure and at least 4 hours apart<br>3 = If elevated, serial CK, CK-MB or Troponin measurements to be done until decline is noted<br>* Written informed consent can be obtained before or after the pre-procedure angiography	Added: Serum Creatinine post procedure (for patients in RSS).<br><br>1 = Cardiac enzymes: CKMB or Troponin I/T must be taken within 7 days prior to the index procedure.<br>2 = Single Arm Study: one sample should be taken in the post-procedure hospitalization period, but no later than 24 hours after the procedure.<br>3 = Randomized Sub-Study: First sample in the post-procedure hospitalization period. A second sample within 3 to 6 hours after the first post-procedure sample, but no later than 24 hours after the procedure.<br>* Written informed consent can be obtained before or after the pre-procedure angiography but prior to any study-related procedure.

Section/Pg in Version 1.0	Version 1.0	Version 2.0
Appendix II		Added: Staged procedure: A staged procedure is defined as the planned treatment of baseline lesions during one or more cardiac catheterization procedure following the index procedure. Revascularization of the baseline lesions treated at the index procedure is not considered a staged procedure. Baseline lesions include all lesions detected during the baseline/index angio.
Appendix II	The intent-to-treat population will consist of all patients (all target lesions) enrolled in the study. For the angiographic sub-study, patients will be analyzed in the treatment group to which they were randomized.<br><br>For the pooled analysis the intent-to-treat population will consist of the intent-to-treat population in the SPIRIT Women Single Arm Study and patients from the intent-to-treat population in the randomized sub-study who were randomized to XIENCE V.	The intent-to-treat population for the angiographic sub-study will consist of all patients (all lesions) randomized, regardless of the treatment actually received. Subjects will be analyzed in the treatment arm to which they were randomized.<br><br>Analysis for the Single Arm Study will be performed on the intent-to-treat population which will include all patients:<br>1. who have signed the Patient Informed Consent<br>2. who have been registered in the ICON IVRS system<br>AND<br>3. who comply to at least one of the following:<br>a. at least one XIENCE V EECS has been implanted<br>b. a non-XIENCE V EECS has been implanted after an intention to or an attempt made to implant a XIENCE V EECS<br>c. no stent was implanted after an intention to or an attempt made to implant a XIENCE V EECS<br><br>Lesions for which there was no attempt to implant a XIENCE V EECS and lesions located in a bypass graft will not be considered in the analysis for the Single Arm Study.<br><br>For the pooled analysis the intent-to-treat population will consist of the intent-to-treat population in the SPIRIT Women Single Arm Study and patients from the intent-to-treat population in the randomized sub-study who were randomized to XIENCE V.

Section/Pg in Version 1.0	Version 1.0	Version 2.0
Appendix II<br>Death	**Death**<br>All deaths are considered cardiac unless an unequivocal non-cardiac cause can be established. Specifically, any unexpected death even in patients with coexisting potentially fatal non-cardiac disease (e.g. cancer, infection) should be classified as cardiac.<br>**Cardiac death:** Any death due to immediate cardiac cause (e.g. MI, low-output failure, fatal arrhythmia). Unwitnessed death and death of unknown cause will be classified as cardiac death. This includes all procedure-related deaths including those related to concomitant treatment.<br>**Vascular death:** Death due to cerebrovascular disease, pulmonary embolism, ruptured aortic aneurysm, dissecting aneurysm, or other vascular cause.<br>**Non-cardiovascular death:** Any death not covered by the above definitions, including death due to infection, sepsis, pulmonary causes, accident, suicide or trauma.<br>2	**Death**<br>Death defined by the Academic Research Consortium is as follows:<br>All death is considered to be cardiac death unless an unequivocal noncardiac cause can be established. Specifically, any unexpected death even in patients with coexisting potentially fatal noncardiac disease (eg, cancer, infection) should be classified as cardiac.<br>**Cardiac death**<br>Any death due to proximate cardiac cause (eg, myocardial infarction, low-output failure, fatal arrhythmia), unwitnessed death and death of unknown cause, and all procedure-related deaths, including those related to concomitant treatment, will be classified as cardiac death.<br>**Vascular death**<br>Death caused by noncoronary vascular causes, such as cerebrovascular disease, pulmonary embolism, ruptured aortic aneurysm, dissecting aneurysm, or other vascular diseases.<br>**Non-cardiovascular death**<br>Any death not covered by the above definitions, such as death caused by infection, malignancy, sepsis, pulmonary causes, accident, suicide, or trauma.

<table>
<tr><th>Section/Pg in Version 1.0</th><th>Version 1.0</th><th>Version 2.0</th></tr>
<tr>
<td>Appendix II Myocardial Infarction</td>
<td>Myocardial Infarction (MI)
In percutaneous coronary intervention trials, initial blood sampling of CK (and CKMB or Troponin if CK is elevated) must be performed prior to the index procedure. Repeat samples should be performed at least twice between 8 and 24 hours following the procedure and at least 4 hours apart.
Peri-Procedural MI:
Is defined as within 48 hours after PCI and within 7 days after CABG.
Peri-Procedural
Total CK >2 times ULN in the presence of a confirming cardiac specific biomarker (a positive value of CK-MB, Troponin I/T) on any one sample obtained after the procedure.
If total CK is not available then CK-MB >3 times ULN is considered evidence of peri-procedural MI. If neither CK nor CK-MB are available then a Troponin
elevation that is >5 times the 99th percentile or diagnostic value for the specific institution is considered evidence of peri-procedural MI.

<u>Peri-procedural MI in the setting of PCI for evolving MI:</u>
1. If the peak total CK (or CK-MB) from the index infarction has not yet been reached: recurrent chest pain lasting >20 minutes (or new ECG changes consistent with acute MI) AND the peak CK (or CK-MB in absence of CK) level measured within 24 hours after the event is elevated by at</td>
<td>Myocardial Infarction (MI)
Myocardial Infarction Classification and Criteria for Diagnosis is defined by the Academic Research Consortium as follows:
<table>
<tr><th><u>CLASSIFICATION</u></th><th><u>BIOMARKER CRITERIA*</u></th><th><u>ADDITIONAL CRITERIA</u></th></tr>
<tr><td>PERIPROCEDURAL PCI</td><td>TROPONIN > 3 X URL OR CK-MB > 3 X URL</td><td>BASELINE VALUE <URL</td></tr>
<tr><td>PERIPROCEDURAL CABG</td><td>TROPONIN > 5 X URL OR CK-MB > 5 X URL</td><td>BASELINE VALUE <URL AND ANY OF THE FOLLOWING: NEW PATHOLOGIC Q WAVES OR LBBB, NEW NATIVE OR GRAFT VESSEL OCCLUSION, IMAGING EVIDENCE OF LOSS OF VIABLE MYOCARDIUM</td></tr>
</table>
</td>
</tr>
</table>

<table>
<tr><th>Section/Pg in Version 1.0</th><th>Version 1.0</th><th>Version 2.0</th></tr>
<tr><td></td><td>least 50% above the previous level.
2. If the elevated CK (or CK-MB) levels from the index infarction are falling or have returned to normal within 24 hours post index PCI: EITHER a new elevation of CK >2 x ULN (or CK-MB >3 x ULN) within 24 hours post index PCI if the CK level has returned to <ULN OR a rise by >50% above the previous nadir level if the CK level has not returned to <ULN.
Post-Intervention MI:
Non-procedural related MI (spontaneous MI) (adapted from ESC/ACC guidelines JACC2000)
Either one of the following criteria satisfies the diagnosis for an acute, evolving or recent MI:
1) Typical rise and gradual fall (Troponin) or more rapid rise and fall (CK-MB) of biochemical markers of myocardial necrosis with at least one of the following:
a) ischemic symptoms;
b) development of pathologic (defined by Minnesota Code) Q waves on the ECG;
c) ECG changes indicative of ischemia (ST segment elevation or depression);
2) Pathologic findings of an acute MI.
3) Development of new pathologic Q-waves on follow-up ECG in the absence of cardiac biomarker assessment during the acute event.
MI after CABG (during Follow-up):</td><td><table>
<tr><td>SPONTANEOUS</td><td>TROPONIN > URL OR CK-MB > URL</td><td></td></tr>
<tr><td>SUDDEN DEATH</td><td>DEATH BEFORE BIOMARKERS OBTAINED OR BEFORE EXPECTED TO BE ELEVATED</td><td>SYMPTOMS SUGGESTIVE OF ISCHEMIA AND ANY OF THE FOLLOWING: NEW ST ELEVATION OR LBBB, DOCUMENTED THROMBUS BY ANGIOGRAPHY OR AUTOPSY</td></tr>
<tr><td>REINFARCTION</td><td>STABLE OR DECREASING VALUES ON 2 SAMPLES WITH A 20% INCREASE 3 TO 6 HOURS AFTER SECOND SAMPLE</td><td>IF BIOMARKERS INCREASING OR PEAK NOT REACHED THEN INSUFFICIENT DATA TO DIAGNOSE RECURRENT</td></tr>
</table></td></tr>
</table>

<table>
<tr><th>Section/Pg in Version 1.0</th><th>Version 1.0</th><th>Version 2.0</th></tr>
<tr><td></td><td>A definite diagnosis of myocardial infarction is made:
1) Within the first 7 days post-intervention (CABG or Stent) when the two following criteria both are positive:
a) Development of new abnormal Q-waves not present on the patient's baseline (i.e. before allocation) ECG. The Minnesota Code for pathologic Q-waves will be used.
b) ➢ Enzyme changes defined as more than 10% of the ratio of [peak CK-MB/ peak total CK] on 3 consecutive samples
OR
➢ Enzyme changes defined as one plasma level CK-MB >5x ULN.
2) Beyond 7 days after any intervention procedure (CABG or Stent) the standard definition for non-procedural related MI immediately above applies.
Non Q-wave Myocardial Infarction (NQMI)
All MIs not classified as Q-wave MI.
Q-wave Myocardial Infarction (QMI)
Development of new pathological Q waves in 2 or more contiguous leads (according to the Minnesota Code as assessed by the CEC) with or without post-procedure CK or CK-MB levels elevated above normal.</td><td><table><tr><td></td><td></td><td>MI</td></tr></table>
URL = UPPER REFERENCE LIMIT (DEFINED 99TH PERCENTILE OF NORMAL REFERENCE RANGE);

LBBB = LEFT BUNDLE-BRANCH BLOCK

* BASELINE BIOMARKER VALUE REQUIRING BEFORE STUDY PROCEDURE AND PRESUMES A TYPICAL RISE AND FALL

• Periprocedural MI After PCI
The periprocedural period includes the first 48 hours after percutaneous coronary intervention.
• Periprocedural MI After CABG
The periprocedural period includes the first 72 hours after coronary artery bypass CABG grafting.
• Spontaneous MI
MI after the periprocedural period may be secondary to late stent complications or progression of native disease. Performance of ECG and angiography supports adjudication to either a target or non-target vessel in most cases. With the unique</td></tr>
</table>

Section/Pg in Version 1.0	Version 1.0	Version 2.0
		issues and pathophysiological mechanisms associated with these later events as well as the documented adverse impact on short-and long-term prognosis, a more sensitive definition than for periprocedural MI of any elevation of troponin above the upper range limit is used. All late events that are not associated with a revascularization procedure simply as spontaneous.<br>• **Electrocardiographic Classification**<br>Within this category Q-wave MI and Non Q-wave MI are distinguished as follows:<br>– Q-wave MI: Development of new pathologicals in 2 or more contiguous leads<br>(according to the Minnesota code or Novacode) with or without post-procedure CK or CK-MB levels elevated above normal.<br>– Non Q-wave MI: All MIs not classified as Q-wave.<br>• **Relation to the Target Vessel**<br>All infarcts that cannot be clearly attributed to a vessel other than the target vessel will be considered related to the target vessel.

Section/Pg in Version 1.0	Version 1.0	Version 2.0
Appendix II Revascularization	**Target Lesion Revascularization (TLR)**<br>TLR is defined as any repeat percutaneous intervention of the target lesion or bypass surgery of the target vessel performed for restenosis or other complication of the target lesion. All TLRs should be classified prospectively as clinically indicated or not clinically indicated by the investigator prior to repeat angiography. An independent angiographic core laboratory should verify that the severity of percent diameter stenosis meets requirements for clinical indication and will overrule in cases where investigator reports are not in agreement.<br>**Target Vessel**<br>The target vessel is defined as the entire major coronary vessel proximal and distal to the target lesion including upstream and downstream branches and the target lesion itself.<br><br>(For example: if the original lesion is the first obtuse marginal branch, the target vessel includes the left main coronary artery, the circumflex coronary artery and its branches).<br><br>Note: in three-vessel treatment every repeat revascularization becomes TVR.<br>**Target Vessel Revascularization (TVR)**<br><br>TVR is defined as any repeat percutaneous intervention or surgical bypass of any segment of the target vessel (as defined above).<br>**Non Target Lesion Revascularization (nonTLR)**<br><br>Any revascularization in a lesion other than the target lesion is considered a	**Revascularization:**<br>Revascularization is defined by the Academic Research<br>Consortium as follows:<br>• **Target Lesion Revascularization (TLR)**<br>Target lesion revascularization is defined as any repeat percutaneous intervention of the target lesion or bypass surgery of the target vessel performed for restenosis or other<br>complication of the target lesion. All target lesion revascularizations should be classified<br>prospectively as clinically indicated* or not clinically indicated by the investigator prior to repeat angiography. An independent angiographic core laboratory should verify that the severity of percent diameter stenosis meets requirements for clinical indication and will overrule in cases where investigator reports are not in agreement.<br>The target lesion is defined as the treated segment from 5 mm proximal to the stent and to 5 mm distal to the stent.<br>• **Target Vessel Revascularization (TVR)**<br>Target vessel revascularization is defined as any repeat percutaneous intervention or surgical bypass of any segment of the target vessel. The target vessel is defined as the entire major coronary vessel proximal and distal to the target lesion, which includes upstream and downstream branches and the target lesion itself.<br>• **Non Target Lesion Revascularization (non-TLR)**<br>Any revascularization in a lesion other than the target lesion is considered a non target lesion revascularization.<br>• **Non Target Vessel Revascularization (non-TVR)**<br>Any revascularization in a vessel other than the target vessel is considered a non-target<br>vessel revascularization.<br>***Clinically indicated revascularization**<br>A revascularization is considered clinically indicated if angiography at follow-up shows a percent diameter stenosis ≥ 50% (core laboratory quantitative coronary angiography assessment) and if one of the following occurs:<br>(1) A positive history of recurrent angina pectoris, presumably related to the target vessel;<br>(2) Objective signs of ischemia at rest (ECG changes) or during exercise test (or equivalent), presumably related to the target vessel;

Section/Pg in Version 1.0	Version 1.0	Version 2.0
	non-TLR.<br><br>**Non Target Vessel Revascularization (non TVR)**<br><br>Any revascularization in a vessel other than the target vessel is considered a non-TVR.	(3) Abnormal results of any invasive functional diagnostic test (eg, Doppler flow velocity reserve, fractional flow reserve);<br>(4) A TLR or TVR with a diameter stenosis ≥ 70% even in the absence of the above-mentioned ischemic signs or symptoms.
Appendix II Stent Thrombosis	**Stent Thrombosis**<br>Stent Thrombosis should be reported as a cumulative value over time and at the various individual time points as specified below. Time 0 is defined as the time point after the guiding catheter has been removed and the patient has left the Cath lab.<br><br>**Timing:**<br><br>Acute stent thrombosis(*): 0 - 24 hours post stent implantation<br>Subacute stent thrombosis(*): >24 hours . 30 days post stent implantation<br>Late stent thrombosis: >30 days - 1 year post stent implantation<br>Very late stent thrombosis: >1 year post stent implantation<br><br>(*) acute/subacute can also be replaced by early stent thrombosis. Early stent thrombosis (0 - 30 days) - this definition is currently used in the community.<br><br>Tree categories of evidence in defining stent thrombosis:	**Stent Thrombosis**<br>Stent thrombosis is defined and discussed by the Academic Research Consortium as follows:<br>Stent thrombosis should be reported as a cumulative value at the different time points and<br>with the different separate time points. Time 0 is defined as the time point after the guiding<br>catheter has been removed and the subject left the catheterization laboratory.<br>**Timing**<br>Acute stent thrombosis* 0-24 hours post stent implantation<br>Subacute stent thrombosis* > 24 hours-30 days post stent implantation<br>Late stent thrombosis† > 30 days-1 year post stent implantation<br>Very late stent thrombosis† > 1 year post stent implantation<br>* Acute/subacute can also be replaced by early stent thrombosis. Early stent thrombos (0-30 days) –*this definition is currently used in the community*.<br>† Including "primary" as well as "secondary" late stent thrombosis; "secondary" late stent thrombosis is a stent thrombosis after a target segment revascularization.<br><br>**Stent Thrombosis Categories: a) Definite b) Probable, and c) Possible**<br><br>**a) Definite stent thrombosis**<br>Definite stent thrombosis is considered to have occurred by either angiographic or pathologic confirmation.

Section/Pg in Version 1.0	Version 1.0	Version 2.0
	1. Definite<br>2. Probable<br>3. Possible<br><br>**<u>1. Definite</u>**<br><br>Definite stent thrombosis is considered to have occurred by either angiographic or pathologic confirmation.<br><br><u>Angiographic confirmation of stent thrombosis</u><br>Thrombolysis In Myocardial Infarction (TIMI) flow is:<br>a. TIMI flow grade 0 with occlusion originating in the stent or in the segment 5mm proximal or distal to the stent region in the presence of a thrombus(*).<br>TIMI flow grade 1, 2, or 3 originating in the stent or in the segment 5mm proximal or distal to the stent region in the presence of a thrombus(*).<br><br>**AND** at least one of the following criteria has been fulfilled within a 48 hours time window:<br><br>1. new onset of ischemic symptoms at rest (typical chest pain with duration >20 minutes)<br>2. new ischemic ECG changes suggestive of acute ischemia<br>3. typical rise and fall in cardiac biomarkers (refer to definition of non-procedural related MI).<br><br>The incidental angiographic documentation of stent occlusion in the absence of clinical syndromes **is not** considered a confirmed stent thrombosis (silent occlusion).	**Angiographic confirmation of stent thrombosis***<br>The presence of a thrombus† that originates in the stent or in the segment 5 mm<br>proximal or distal to the stent and presence of at least 1 of the following criteria<br>within a 48-hour time window:<br>• Acute onset of ischemic symptoms at rest<br>• New ischemic ECG changes that suggest acute ischemia<br>• Typical rise and fall in cardiac biomarkers (refer to definition of<br>• spontaneous MI) Nonocclusive thrombus<br>-Intracoronary thrombus is defined as a (spheric, ovoid, or irregular)<br>noncalcified filling defect or lucency surrounded by contrast material<br>(on 3 sides or within a coronary stenosis) seen in multiple projections,<br>or persistence of contrast material within the lumen, or a visible embolization of intraluminal material downstream.<br>• Occlusive thrombus<br>-TIMI 0 or TIMI 1 intrastent or proximal to a stent up to the most adjacent proximal side branch or main branch (if originates from the<br>side branch).<br>***The incidental angiographic documentation of stent occlusion in the absence of<br>clinical signs or symptoms is not considered a confirmed stent thrombosis (silent occlusion).<br>†Intracoronary thrombus.<br><br>**Pathological confirmation of stent thrombosis**<br>Evidence of recent thrombus within the stent determined at autopsy or via examination of tissue retrieved following thrombectomy.<br><br>**b) Probable stent thrombosis**<br>Clinical definition of probable stent thrombosis is considered to have occurred after

Section/Pg in Version 1.0	Version 1.0	Version 2.0
	(*) Intracoronary thrombus [Ellis et al., Mabin et al., Capone et al.]<br>*Non-occlusive thrombus:*<br>Intracoronary thrombus is defined as a (spheric, ovoid or irregular) non-calcified filling defect or lucency surrounded by contrast material (on three sides or within a coronary stenosis) seen in multiple projections, or persistence of contrast material within the lumen, or a visible embolization of intraluminal material downstream.<br>*Occlusive thrombus:*<br>A TIMI 0 or TIMI 1 intra-stent or proximal to a stent up to the most adjacent proximal side branch or main branch (if originating from the side branch).<br><br>Pathologic confirmation of stent thrombosis<br>Evidence of recent thrombus within the stent determined at autopsy or via examination of tissue retrieved following thrombectomy.<br>**2. Probable:**<br><br>Clinical definition of probable stent thrombosis is considered to have occurred after intracoronary stenting in the following cases:<br>1. Any unexplained death within the first 30 days.<br>2. Irrespective of the time after the index procedure any myocardial infarction (MI), which is related to documented acute ischemia in the territory of the implanted stent without angiographic confirmation of stent thrombosis and in the absence of any other obvious cause.<br>**3. Possible:**	intracoronary stenting in the following cases:<br>• Any unexplained death within the first 30 days‡<br>• Irrespective of the time after the index procedure, any MI that is related to documented acute ischemia in the territory of the implanted stent without angiographic confirmation of stent thrombosis and in the absence of any other obvious cause<br>‡ For studies with ST-elevation MI population, one may consider the exclusion of unexplained death within 30 days as evidence of probable stent thrombosis.<br><br>**c) Possible stent thrombosis**<br>Clinical definition of possible stent thrombosis is considered to have occurred with any unexplained death from 30 days after intracoronary stenting until end of trial follow-up.

Section/Pg in Version 1.0	Version 1.0	Version 2.0
	Clinical definition of possible stent thrombosis is considered to have occurred with any unexplained death from 30 days following intracoronary stenting until end of trial follow-up.	
Appendix VII	Koen Mol<br>Clinical Project Manager<br>Tel: + 32 2 416 99 92<br>Fax: + 32 2 714 16 15<br>Mobile: + 32 476 86 99 40<br>e-mail: koen.mol@av.abbott.com	Bridget Hurley<br>Director, International Clinical Operations<br>International Clinical Operations<br>Abbott Vascular International BVBA<br>Park Lane - Culliganlaan 2B<br>1831 Diegem, Belgium<br>Tel: +32 2 416 9223<br>Fax:+ 32 2 416 9101<br>Email : bridget.hurley@av.abbott.com
	A list of monitors can be obtained upon request from the Clinical Project Manager<br><br>Copies of training records and CVs for all Sponsor study monitors are held on file in the Abbott S.A. International Clinical Operations office	Deleted

Section/Pg in Version 2.0	Version 2.0	Version 3.0
**Page 18 – Paragraph 6.2.1.**	Clinical evaluation-specific procedures or alterations of patient care cannot be started until a signed informed consent has been obtained. The investigator or a person designated by the investigator who has been trained on the clinical investigation plan, will explain the nature and scope of the clinical evaluation, potential risks and benefits of participation, and answer the patient's questions. If the patient agrees to participate, the informed consent form must be signed and personally dated by the patient or legally authorized representative. The investigator must also sign and date the informed consent, prior to enrollment of the patient. A copy of the completed informed consent form must be provided to the patient. The obtaining of the consent, provision of a copy to the subject, along with the date and time should be documented in the subject's medical records and signed by the investigator.<br>All patients must provide informed consent in accordance with the local Medical Ethics Committee (MEC) requirements, using an MEC-approved informed consent form. The final eligibility for the clinical evaluation will be confirmed based on the pre-intervention angiography. Appendix VI outlines the screening process and illustrates the point where informed consent should be obtained.<br>22.1.1.1.5	Informed Consent needs to be signed and dated by the investigator and the patient or by her legal representative before any study-related procedure and before the pre-procedure angiographic screening procedure. The investigator or a person designated by the investigator who has been trained on the clinical investigation plan, will explain the nature and scope of the clinical evaluation, potential risks and benefits of participation, and answer the patient's questions. If the patient agrees to participate, the informed consent form must be completed, signed and personally dated by the patient or legally authorized representative. A copy of the completed informed consent form must be provided to the patient. The obtaining of the consent, provision of a copy to the subject, along with the date and time should be documented in the subject's medical records and signed by the investigator.<br>All patients must provide informed consent in accordance with the local Medical Ethics Committee (MEC) requirements, using an MEC-approved informed consent form. Appendix VI outlines the screening process and illustrates the point where informed consent should be obtained.
**Page 22 – Paragraph 7.2.**	After meeting the general inclusion and exclusion criteria, written informed consent can be obtained before or after the pre-procedure angiography. The angiograms acquired just before the intended procedure (i.e. the baseline angiography) will be used to assure by visual assessment that the reference diameter(s) is ≥ 2.5* mm and ≤ 4.0 mm and the lesion length <28mm.<br>*expanded to 2.25 in diameter when 2.25 mm stent available<br>Once the angiographic inclusion criteria have been confirmed, the patient should be registered via the IVRS system.<br>Due to phased start-up of the clinical sites and to allow all sites to have the opportunity to enroll patients in the study, sites may	After meeting the general inclusion and exclusion criteria, written informed consent must be obtained before the pre-procedure angiography. The angiograms acquired just before the intended procedure (i.e. the pre-procedure angiography) will be used to assure by visual assessment that the reference diameter(s) is ≥ 2.5* mm and ≤ 4.0 mm and the lesion length <28mm.<br>*expanded to 2.25 in diameter when 2.25 mm stent available<br>Once the angiographic inclusion criteria have been confirmed, the patient should be registered via the IVRS system.<br>Due to phased start-up of the clinical sites and to allow all sites to have the opportunity to enroll patients in the study, sites may be asked to

Section/Pg in Version 2.0	Version 2.0	Version 3.0
	be asked to delay further recruitment once they have enrolled 50 patients in the XIENCE V SPIRIT WOMEN Single Arm Study.	delay further recruitment once they have enrolled 50 patients in the XIENCE V SPIRIT WOMEN Single Arm Study.
**Page 25 – Paragraph 8.1.**	*Following the 2 year follow-up, it is only mandatory to report and collect information on cardiac adverse events and serious adverse events.	*Following the 2 year follow-up, it is only mandatory to report and collect information on serious adverse events, cardiac adverse events and device related events.
**Page 28 – Paragraph 8.3.2.**	An adverse event may be expected, (however should still be reported through 2 year follow-up).	An adverse event may be expected, (however should still be reported).
**Page 42 – Appendix I – Declaration of Helsinki**<br><br>**Treatment Strategy p. 6**	Declaration of Helsinki, dated October 9, 2004	Declaration of Helsinki, dated October 23, 2008
**Page 61 – Appendix V – Schedule of Events**	* Written informed consent can be obtained before or after the pre-procedure angiography but prior to any study-related procedure.	* Written informed consent must be obtained before the pre-procedure angiography and prior to any study-related procedure.
**Page 62 – Appendix VI – Screening Process**	The patient must be reported as screen-failure as from the moment the patient does not fulfill the general in – and exclusion criteria.	The patient must be reported as screen-failure as from the moment the patient has signed and dated the informed consent.
**Page 65 – Appendix VIII - REVISION HISTORY XIENCE V SPIRIT Women Protocol**	V 1.0 Dated 19 March 2007 to V 2.0 Dated 19 August 2008	V 1.0 Dated 19 March 2007 to V 2.0 Dated 19 August 2008 &<br>V 2.0 Dated 19 August 2008 to V 3.0 Dated 19 November 2008

Section/Pg in Version 4.0	Version 3.0	Version 4.0
Throughout document	XIENCE V SPIRIT Women	SPIRIT Women
Throughout document	XIENCE V EECSS	XIENCE EECSS (XIENCE V EECSS and XIENCE PRIME EECSS)
Throughout document		General formatting
Front Cover	Version Number: Version 3.0<br>Date: 10 December 2008	Version Number: Version 4.0<br>Date: 21 August 2009
Revision History<br>P. 2		**Amendment to Version 3.0 Dated 24 August 2009**<br>The XIENCE PRIME Everolimus Eluting Coronary Stent System (EECSS) received CE Mark in June 2009. The CE Mark for the XIENCE PRIME EECSS was obtained based upon the consideration that the clinical data of the SPIRIT trials are also applicable to the XIENCE PRIME EECSS. The SPIRIT trials have established the safety and performance of the XIENCE V EECSS and therefore no additional clinical study data were required for the XIENCE PRIME EECSS.
Clinical Investigation Plan Summary<br>p. 6	**Objectives**<br>• Continued assessment of the XIENCE V Everolimus Eluting Coronary Stent System (XIENCE V EECSS) with the primary focus on clinical outcomes in the treatment of female patients with de novo coronary artery lesions<br>• Characterization of the female population undergoing stent implantation with XIENCE V	**Objectives**<br>• Continued assessment of the XIENCE Everolimus Eluting Coronary Stent System (XIENCE V EECSS and XIENCE PRIME EECSS) with the primary focus on clinical outcomes in the treatment of female patients with de novo coronary artery lesions<br>• Characterization of the female population undergoing stent implantation with XIENCE EECSS

Section/Pg in Version 4.0	Version 3.0	Version 4.0
	**Clinical Investigation Design** — The XIENCE V SPIRIT WOMEN Clinical Evaluation is a prospective, open label, single arm, multi-center study evaluating performance of the XIENCE V EECSS in the treatment of female patients with coronary artery lesions, per its Instructions for Use (IFU).<br>…<br>The test device used is XIENCE V Everolimus Eluting Coronary Stent System (XIENCE V EECSS). The XIENCE V EECSS has received CE mark approval on 30 January 2006 and FDA approval on 02 July 2008.	**Clinical Investigation Design** — The SPIRIT WOMEN Clinical Evaluation is a prospective, open label, single arm, multi-center study evaluating performance of the XIENCE EECSS in the treatment of female patients with coronary artery lesions, per the Instructions for Use (IFU).<br>…<br>The test devices used are the XIENCE V Everolimus Eluting Coronary Stent System (XIENCE V EECSS) and the XIENCE PRIME Everolimus Eluting Coronary Stent System (XIENCE PRIME EECSS (RSS only)). The XIENCE V EECSS has received CE mark approval on 30 January 2006 and FDA approval on 02 July 2008. The XIENCE PRIME EECSS has received CE mark on 23 June 2009.
Clinical Investigation Plan Summary<br>p. 10		**Randomized Sub-study:**<br>The test devices used are the XIENCE V Everolimus Eluting Coronary Stent System (XIENCE V EECSS and the XIENCE PRIME Everolimus Eluting Coronary Stent System (XIENCE PRIME EECSS.<br>…<br>If two or more target lesions are to be treated and the patient is randomized to the XIENCE EECSS, all lesions must receive either the XIENCE V EECSS or the XIENCE PRIME EECSS.
2.1.1<br>p. 12	The XIENCE V EECSS has received CE mark approval on 30 January 2006 and FDA approval 02 July 2008. In light of that, the SPIRIT V study has begun enrollment in approximately 100 sites outside the United States, and will enroll around 3000 patients. It consists of two concurrent studies: a prospective, randomized, active-controlled, single blind, parallel two-arm multicenter study comparing XIENCE V to the Taxus Liberté™ in the treatment of diabetic patients with coronary artery lesions	The XIENCE V EECSS has received CE mark approval on 30 January 2006 and FDA approval 02 July 2008. In light of that, the SPIRIT V study has completed enrollment in approximately 100 sites outside the United States, and enrolled 3025 patients. It consists of two concurrent studies: a prospective, randomized, active-controlled, single blind, parallel two-arm multicenter study comparing XIENCE V to the Taxus Liberté™ in the treatment of diabetic patients with coronary artery lesions and a prospective, single arm, multi-center registry evaluating

Section/Pg in Version 4.0	Version 3.0	Version 4.0
	and a prospective, single arm, multi-center registry evaluating performance of XIENCE V in real-world use.	performance of XIENCE V in real-world use. In the registry, 30 day rates of in-hospital non-Q wave MI (1.9%), acute stent thrombosis (0.15%) and sub-acute stent thrombosis (0.3%) supported the acute safety of the XIENCE V EECSS. At 1 year the composite of Cardiac Death, MI attributed to the target vessel and TLR was 5.1% and the late definite and probable stent thrombosis was 0.23% with a cumulative rate of definite and probable stent thrombosis of 0.66% thus demonstrating the continued safety and efficacy of the XIENCE V EECSS.
2.2<br>p. 12	The test device used in the XIENCE V SPIRIT WOMEN Clinical Evaluation is XIENCE V Everolimus Eluting Coronary Stent System (XIENCE V EECSS). The XIENCE V EECSS has received CE mark approval on 30 January 2006 and FDA approval 02 July 2008. In this Clinical Investigation Plan (CIP), the following abbreviations are used with regards to the XIENCE V.	One of the test devices used in the SPIRIT WOMEN Clinical Evaluation is XIENCE V Everolimus Eluting Coronary Stent System (XIENCE V EECSS). The XIENCE V EECSS has received CE mark approval on 30 January 2006 and FDA approval 02 July 2008. In this Clinical Investigation Plan (CIP), the following abbreviations are used with regards to the XIENCE V.
2.3<br>p. 14	***2.3 Description of the CYPHER SELECT™ Plus***	***2.3 Description of XIENCE PRIME EECSS***<br>The second test device used in the SPIRIT WOMEN Clinical Evaluation (RSS only) is XIENCE PRIME Everolimus Eluting Coronary Stent System (XIENCE PRIME EECSS). The XIENCE PRIME EECSS has received CE mark approval on 23 June 2009. The XIENCE PRIME EECSS is manufactured by Abbott Cardiovascular Systems, Inc., California, an affiliate of Abbott Vascular Inc.<br>The XIENCE PRIME stent system is similar to that of the Abbott Vascular XIENCE V Everolimus Eluting Coronary Stent System (EECSS) (CE Mark, January 2006). The XIENCE PRIME stent uses the identical stent, balloon material, and drug coating formulation as the XIENCE V stent. However, the XIENCE PRIME stent design has been slightly modified from that of the XIENCE V stent in order to accommodate design improvements while not affecting the overall structural integrity of the design. These modifications include longer cell length for optimized stent retention, taller non-linear links for improved flexibility, straighter bar arms to reduce strut interference and to better maintain

Section/Pg in Version 4.0	Version 3.0	Version 4.0
		coating integrity during the crimping process, and a modified proximal end ring to reduce strut lifting. The delivery system utilizes the same principle of operation as other Abbott Vascular Rapid Exchange (RX) coronary stent systems and coronary dilatation catheters.<br>There are two stent designs for the XIENCE PRIME stent. The small XIENCE PRIME stent design is intended for the 2.25, 2.5 and 3.0 mm expansion diameters while the medium XIENCE PRIME stent design is intended for the 3.5 and 4.0 mm expansion diameters.<br>***2.4 Description of the CYPHER SELECT™ Plus***
4<br>p. 14	The test device used is XIENCE V Everolimus Eluting Coronary Stent System (XIENCE V EECSS). The XIENCE V EECSS has received CE mark approval on 30 January 2006 and FDA approval 02 July 2008.	The test device used is XIENCE Everolimus Eluting Coronary Stent System (XIENCE V EECSS and XIENCE PRIME EECSS (randomized sub-study only)). The XIENCE V EECSS has received CE mark approval on 30 January 2006 and FDA approval 02 July 2008. The XIENCE PRIME EECSS has received CE mark approval on 23 June 2009.
4.2<br>p.16		The test devices used are the XIENCE V Everolimus Eluting Coronary Stent System (XIENCE V EECSS and the XIENCE PRIME Everolimus Eluting Coronary Stent System (XIENCE PRIME EECSS.<br>…<br>If two or more target lesions are to be treated and the patient is randomized to the XIENCE EECSS, all lesions must receive either the XIENCE V EECSS or the XIENCE PRIME EECSS.
8.1<br>p. 25	• * Following the 2 year follow-up, it is only mandatory to report and collect information on serious adverse events, cardiac adverse events and device related events	* Following the 2 year follow-up, it is mandatory only to report and collect information on serious adverse events, cardiac adverse events and device related events.
9.1<br>p.31	H_0: In-stent $LL_{XIENCE\ V}$ - In-stent $LL_{CYPHER} \geq \delta$<br>H_A: In-stent $LL_{XIENCE\ V}$ - In-stent $LL_{CYPHER} < \delta$	H_0: In-stent LL_{XIENCE} - In-stent $LL_{CYPHER} \geq \delta$<br>H_A: In-stent LL_{XIENCE} - In-stent $LL_{CYPHER} < \delta$

**Section/Pg in Version 4.0**	**Version 3.0**	**Version 4.0**
21<br>p. 64	Bridget Hurley<br>Director, International Clinical Operations<br>Abbott Vascular International BVBA<br>Park Lane - Culliganlaan 2B<br>1831 Diegem, Belgium<br>Tel: +32 2 416 9223<br>Fax:+ 32 2 416 9101<br>Email: bridget.hurley@av.abbott.com	Bridget Hurley<br>Director, Clinical Operations<br>Abbott Vascular International BVBA<br>Park Lane - Culliganlaan 2B<br>1831 Diegem, Belgium<br>Tel: +32 2 416 9223<br>Fax:+ 32 2 416 9101<br>Email: bridget.hurley@av.abbott.com
22<br>p. 65	V 1.0 Dated 19 March 2007 to V 2.0 Dated 19 August 2008 &<br>V 2.0 Dated 19 August 2008 to V 3.0 Dated 10 December 2008	V 1.0 Dated 19 March 2007 to V 2.0 Dated 19 August 2008 &<br>V 2.0 Dated 19 August 2008 to V 3.0 Dated 10 December 2008<br>V 3.0 Dated 10 December 2009 to V 4.0 Dated 25 August 2009

23 Appendix IX References

[i] Stramba-Badiale M, Fox KM, Priori SG, Collins P, Daly C, Graham I, Jonsson B, Schenck-Gustafsson K, Tendera M. Cardiovascular diseases in women: a statement from the policy conference of the European Society of Cardiology. Eur. Heart J. 2006: 27: 994-1005.

[ii] Milan E. Coronary Artery Disease: the other half of heaven. Q. J. Nucl. Med. Mol. Imaging 2005 49: 72-80.

[iii] Mikhail GW. Coronary heart disease in Women is underdiagnosed, undertreated and underresearched. BMJ 2005: 331:467-468

[iv] CIP 00-337, "PENTA Registry Final Report" (P970020, May 7, 2001)

[v] CIP 95-004, "ASCENT Trial Final Report" (P970020, October 2, 1997)

[vi] CIP 96-003, "LONGS RX Trial Final Report" (P970020, June 4, 1997)

[vii] CIP 96-006, "HP Registry Final Report (P970020, June 4, 1997)

[viii] CIP 97-313, "RECREATE Trial Final Six Months Report" (P970020, August 12, 1998)

[ix] CIP 97-315, "DUET Registry Final Report" (P970020, May 17, 1999)

[x] CIP 99-328, "TETRA Registry Final Report" (P970020/S023, October 3, 2000)

[xi] Baim DS, Cutlip DE, Midei M, et al. Final Results of a Randomized Trial Comparing the MULTI-LINK Stent With the Palmaz-Schatz Stent for Narrowings in Native Coronary Arteries

[xii] Kereiakes DJ, Midei M, Hermiller J, et al. Procedural and Late Outcomes Following MULTI-LINK DUET Coronary Stent Deployment. *Am. J Cardiol.* 1999;84:1385-1390

[xiii] Sousa JE, Costa MA, Abizaid AC, Rensing BJ, Abizaid AS, Tanajura LF, Kozuma K, Van Langenhove G, Sousa AG, Falotico R, Jaeger J, Popma JJ, Serruys PW. Sustained Suppression of Neointimal Proliferation by Sirolimus-Eluting Stents: One-Year Angiographic and Intravascular Ultrasound Follow-Up. *Circulation*. 2001;104:2007.

[xiv] Stone GW, Ellis SG, Cox DA, Hermiller J, O'Shaughnessy C Mann JT, Turco M, Caputo R, Bergin P, Greenberg J, Popma J, Koglin J, Russel ME. A polymer based paclitaxel-eluting stent in patients with coronary artery disease. N Engl J Med 2004;350:221-231.

[xv] Colombo A. Taxus II International Study: Cohort I Slow Release Formulation; Six-Month Results Intent to Treat Analysis. Taxus II International Study: Cohort II Moderate Release Formulation; Six-Month Results Intent to Treat Analysis. TCT 2002 Scientific Session.

[xvi] Moses, J. W., M. B. Leon, et al. Sirolimus eluting stents versus standard stents in patients with stenosis in a native coronary artery. *New England Journal of Medicine*. 2003, 349: 1315-1323.

[xvii] Farb, A., M. John, et al. Oral everolimus inhibits in-stent neointimal growth. *Circulation*, 2002, 106: 2379-2384.

[xviii] Clappers N, Verheugt FWA Hotline sessions of the 28th European Congress of Cardiology / World Congress of Cardiology 2006. EHJ 2006; 27: 2896-2899

[xix] Reality Trial, Presentation, ACC, Orlando, March 2005. MC Morice

[xx] Sirtax Trial, Presentation, ACC, Orlando, March 2005. S Windecker

[xxi] ISAR Diabetes Trial, Presentation, ACC, Orlando, March 2005. A Kastrati

[xxii] Petronio AS, De Carlo M, Branchitta G, Papini B, Ciabatti N, Gistri R, Cortese B, Gherarducci G, Barsotti A. Randomized comparison of Sirolimus and Paclitaxel drug-eluting stents for long lesions in the left anterior descending artery. An intravascular ultrasound study. J. Am. Coll. Cardiol. 2007; 49:539-546.

[xxiii] Silber, S Cypher versus Taxus: Are there differences? J. Interven. Cardiol. 2005, 18: 441-446.

[xxiv] CIP 00-337, "PENTA Registry Final Report" (P970020/May 7, 2001).

[xxv] Fischman DL, Leon MB, Baim DS, Schatz RA, Penn I, Detre K, Veltri L, Ricci D, Nobuyoshi M, Cleman M, Heuser R, Almond D, Teirstein PS, Fish RD, Colombo A, Brinker J, Moses J, Shaknovich A, Hirshfeld J, Bailer S, Ellis S, Rake R, Goldberg S. A Randomized Comparison of Coronary Stent Placement and Balloon Angioplasty in the Treatment of Coronary Artery Disease. N Engl J Med 1994;331:496:501

[xxvi] Grube, E., U. Gerckens, et al. First human experience using a new everolimus stent coating: Early finding of the FUTURE Trial. *American Journal of Cardiology*, 2002, 90(Suppl 6A): 71H.

[xxvii] RAD-01-02-KM., P. (November 7, 2003). "FUTURE II Clinical Trial 6-month Report."

[xxviii] Serruys PW, Ong ATL, Piek JJ, Neumann FJ, van der Giessen WJ, Wiemer M, Zeiher A, Grube E, Haase J, Thuesen L, Hamm C, Otto-Terlouw PC. A Randomized Comparison of a Durable Polymer Everolimus-Eluting Stent with a Bare Metal Coronary Stent: The SPIRIT First Trial. *EuroIntervention* 2005;1: 266-272.
